# Substance use and suicidal ideation and behaviour in low- and middle-income countries: a systematic review

**DOI:** 10.1186/s12889-018-5425-6

**Published:** 2018-04-24

**Authors:** Elsie Breet, Daniel Goldstone, Jason Bantjes

**Affiliations:** Department of Psychology, Stellenbosch University, Private Bag X1 Matieland, Stellenbosch, 7602 South Africa

**Keywords:** Substance use, Suicidal ideation and behaviour, Suicide prevention, Low- and middle-income countries, Systematic review

## Abstract

**Background:**

Understanding relationships between substance use and suicidal ideation and behaviour (SIB) has important public health implications for suicide prevention in low- and middle-income countries (LMICs), where 75% of suicides occur. This systematic review explored the associations between substance use and SIB in LMICs.

**Methods:**

We searched five databases using a combination of keywords for substance use, SIB and LMICs to identify English-written quantitative studies published between January 2006 and February 2016. Data were extracted to provide an overview of what is known about the topic, highlight gaps in the literature, and explore the implications of current knowledge for suicide prevention. Studies included in the review were assessed for methodological quality using the Scottish Intercollegiate Guidelines Network checklist.

**Results:**

Analysis of included studies (*N* = 108) demonstrated a consistent positive association between substance use and SIB across all substances (i.e. alcohol, tobacco, cannabis, illicit drugs, non-medical use of prescription drugs), all substance use dimensions (i.e. intoxication, use, and pathological use) and all SIB dimensions (i.e. suicidal ideation, non-fatal suicidal behaviour, and suicide). Most of the available research evidence comes from upper-middle-income countries, only 22% comes from lower-middle-income and low-income countries. Most studies focused on alcohol and tobacco, while neglecting substances such as cannabis, opioids, sedatives, stimulants, misuse of prescription medication, inhalants, and hallucinogens. Most of the studies employed a cross-sectional design, were conducted within a risk-factor paradigm, and provided little information about the potential interaction between variables.

**Conclusions:**

Public health suicide prevention policy and research in LMICs should take account of the fact that: substance use is a potentially modifiable risk factor; assessment and management of substance use is integral to the care of at-risk patients; reducing consumption and hazardous use of substances in LMICs is important for suicide prevention; and research needs to be expanded to include more theory driven research that focuses on all substance use dimensions and SIB dimensions, while employing more sophisticated statistical methods.

**Electronic supplementary material:**

The online version of this article (10.1186/s12889-018-5425-6) contains supplementary material, which is available to authorized users.

## Background

Suicide prevention, particularly in low- and middle-income countries (LMICs), is a serious public health challenge. Suicide is the 15th leading cause of death worldwide, with more than 800,000 people dying by suicide each year [1]. Seventy-five percent of suicides occur in LMICs, where the estimated age-standardized suicide rate is 11·2 per 100,000 people and the male-to-female ratio is 1·6:1 [1]. Research from high-income countries (HICs) suggests that substance use is a potentially modifiable risk factor for suicide [1]. Global action plans and strategies endorsed by the World Health Assembly consider substance use a priority area for global action in preventing suicide. The World Health Organization (WHO) Mental Health Action Plan 2013 to 2020 aims to reduce suicide rates by 10% across countries [2]. It has been proposed that suicide prevention efforts should focus on the full range of suicidal phenomena, including suicidal ideation and non-fatal suicidal behaviour [1]. It is within this context that we set out to conduct a systematic review of literature published in English reporting on the relationships between substance use and suicidal ideation and behaviour in LMICs. We provide a synthesis of the research in this area and highlight gaps in the literature. The findings of this systematic review will be of interest to public health policy makers and researchers who are concerned about suicide prevention in LMICs.

For the purpose of this review, suicidal ideation refers to any thoughts of death, intention to kill oneself, or plan to end one’s life. Non-fatal suicidal behaviour is understood as intentional self-injurious behaviour that is non-habitual and with a non-fatal outcome [1, 3]. Suicide refers to the act of deliberately killing oneself [1], and is synonymous with fatal suicidal behaviour. The term suicidal ideation and behaviour (SIB) is used to denote the full spectrum of suicidal phenomena, including suicidal ideation, non-fatal suicidal behaviour and suicide.

Globally, harmful alcohol use is implicated in an estimated 3·3 million deaths annually (5·9% of all deaths) and contributes to approximately 4·6% of disability-adjusted life years [4, 5]. Rates of deaths attributable to alcohol are almost double among males (7·6%) when compared with females (4%) worldwide [5]. Illicit drug use also constitutes a significant risk to the global burden of disease and disability. Approximately one out of every 20 people aged 15 to 64 years old reported illicit drug use at least once during 2013, where the substances used were commonly cannabis, opioids, cocaine and amphetamines [6]. A mortality rate of 40·8 drug-related deaths per million people aged 15 to 64 years old was reported in 2013, with drug overdose being the most common cause of death [6]. Across all drug types, it is estimated that roughly two thirds of the years of life lost and lived with disability are attributed to men [6]. Harmful alcohol use and illicit drug use estimates in LMICs vary greatly across settings and the available information is limited by inadequate national registries in these countries [5]. For example, 40% of the alcohol consumed in low-income countries (LICs) is unrecorded [1].

It is well established that SIB is associated with psychopathology, principally depressive disorders, bipolar mood disorders, personality disorders and psychotic illnesses [7, 8]. However, substance use and substance use disorders are also clearly associated with increased risk of SIB. For example, alcohol plays a role in every fifth suicide [5], while tobacco and illicit drug use (e.g. cannabis and heroin) have been positively associated with SIB [9]. The current evidence base for the association between substance use and SIB comes predominantly from research conducted in HICs [10]. In recent years, the association between substance use and SIB in LMICs has received increasing attention [1].

A number of hypotheses and theories have been advanced in an effort to explain the links between substance use and SIB. Biological theories postulate that substance use (e.g. acute intoxication that increases impulsivity or disinhibition and impairs judgement and problem-solving abilities) represents a vulnerability or predisposition (diathesis) to SIB. Within this model, stressful events (e.g. a depressive episode or relationship conflict) act as triggers which can lead to SIB [11]. The sociological theory of SIB suggests that the risk for SIB is inversely associated with the degree of social integration or regulation [12]. Problematic substance use may hold destructive consequences for social integration and disturb social regulation, which in turn leads to SIB [11]. Interpersonal theory builds on this premise by postulating that suicide risk results from the simultaneous experience of a low sense of belongingness or connectedness, perceived burdensomeness and the capability to engage in SIB [13, 14]. Social epidemiological theories posit that an individual’s risk for suicide depends not only on their personal experiences, but also on the interplay between cultural, economic, social and environmental factors [15, 16].

While research on the association between substance use and SIB is receiving increased attention in many HICs, the topic has been rather absent from research conducted in LMICs. What is true in one context cannot simply be accepted to be true in another setting. As such, suicide prevention in LMICs may not be appropriate if it is based on models employed in HICs that are not challenged by poverty and have greater government involvement in issues related to substance use and SIB. Therefore, context specific research is imperative if we aim to develop and implement effective suicide prevention interventions in LMICs. No systematic review has explored the association between substance use and SIB in LMICs. Previous reviews have established an association between alcohol [11, 17–23], tobacco [24], or illicit drug [17, 21, 22, 25] use and SIB dimensions, yet none have focused on all substance types and a full range of SIB across all LMICs. It is within this context that we investigate: (a) what is known about the ways in which substance use is associated with SIB in LMICs; (b) what remains unknown about the ways in which substance use is associated with SIB in LMICs; and (c) where researchers should focus their attention with regards to the ways in which substance use is implicated in SIB in LMICs.

## Methods

### Search strategies

A comprehensive search strategy was developed in accordance with the Preferred Reporting Items for Systematic Reviews and Meta-Analyses checklist (PRISMA). We searched: PubMed/MEDLINE, CINHAL Plus (EBSCO), DARE (Database of Abstracts of Reviews of Effectiveness), Web of Science and PsycINFO (OvidSP) databases. A search strategy was designed for PubMed that combined keywords for SIB, substances, and LMICs. This strategy was then adapted for each subsequent database (Additional file 1: Appendix A).

We searched for studies with titles and abstracts published in English between 1 January 2006 and 10 February 2016. We limited our search to this period because an initial mapping exercise demonstrated that prior to 2006 there were few studies in LMICs that included robust methodologies investigating the association between substances and SIB. Reference lists of all included review articles were searched for relevant publications that had not been included after searching the databases.

### Search terms

We included all substances and all substance use dimensions (i.e. use, misuse, intoxication, withdrawal) identified in the substance-related and addictive disorders chapter of the Diagnostic and Statistical Manual of Mental Disorders (DSM) 4th edition [26] and 5th edition [27]. We excluded studies that did not distinguish between illicit and prescribed drugs. Considering the classification of SIB used by the WHO in 2014 [1], this review included a broad range of search terms for SIB, including suicidal thoughts/ideation, suicide plan, self-harm, attempted suicide, and suicide. Studies related to violence, terrorism and assisted suicide were not included in this review. Search terms used to capture studies from LMICs included all the individual countries on the list of LMICs from the World Bank in 2016 (Additional file 1: Appendix B) [28]. LMICs can be divided into LICs and middle-income countries (MICs), while MICs can be further divided into lower-middle-income countries (LMCs) and upper-middle-income countries (UMCs). All search terms included MeSH terms/subject headings.

### Types of studies and participants

Studies reporting data on measures of the association or relationship between substances and SIB in LMICs were included in this review. We included cross-sectional studies, cohort studies, case-control studies, interrupted-time series studies, before-and-after studies, ecological studies and economic studies. Case report and case series studies were excluded. All included studies had to report quantitative data for bivariate or multivariate analyses that tested the association or relationship between substance use and SIB. All studies that reported descriptive statistics only were not included in this review. In cases where a study reported both qualitative and quantitative findings, only the quantitative findings were reported.

### Identification of studies

**Fig. 1:**
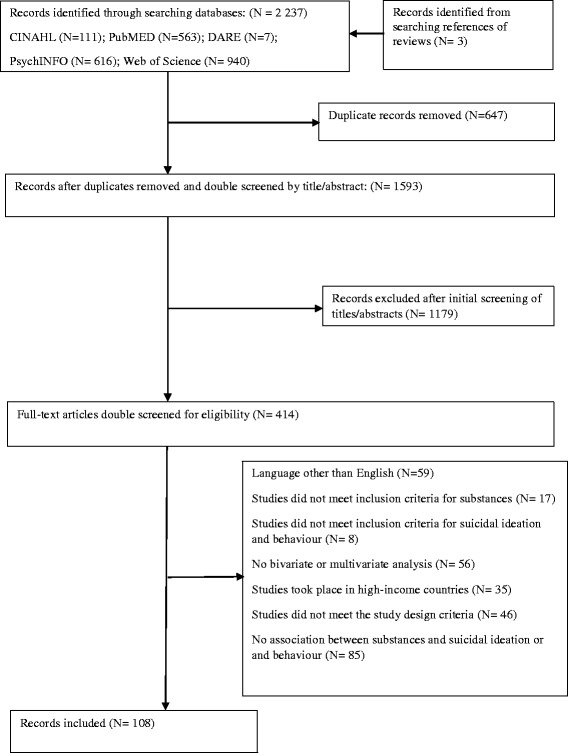
Flow chart of study search and selection process

EB and DG conducted the literature search. A total of 2237 articles were identified of which 647 were removed as duplicates (Fig. 1). After testing agreement on 30 articles, EB and DG independently reviewed the titles and abstracts of 1593 articles to identify those articles that reported findings for an association or relationship between substance use and SIB. The two authors did not discuss any of the articles during this screening process. Once compared, discrepancies were discussed until agreement was reached. If agreement could not be reached, JB was consulted. This process yielded a total of 414 articles that met the initial screening criteria. Two authors independently screened the full-text of 414 articles against the inclusion and exclusion criteria, leaving 108 studies included in the systematic review. The literature search and screening process was managed using Zotero.

### Data extraction and management

Data were extracted from the 108 included studies. The data that were extracted included: (a) study characteristics (author; year; country; region; data collection start and end dates; rural/urban; study setting, aim and design; sample size; population age, gender and ethnicity); (b) substance use dimensions; (c) SIB dimensions; and (d) association/relationship between substances and SIB (odds ratios and 95% confidence intervals). The data extraction was checked by EB for potential missing data, errors and statistical accuracy. We contacted authors in cases where data was not reported or information was unclear. Data extraction and presentation of the tables included in this systematic review was guided by other systematic reviews that focused on poverty in LMICs [29, 30].

### Assessment of methodological quality

Quality and appropriateness of the included studies were assessed by DG and EB. A set of predetermined criteria derived from the Scottish Intercollegiate Guidelines Network checklist was used to assess the methodological quality and appropriateness of the studies included in this review (Additional file 1: Table S1 and Table S2) [31]. Due to the large number of articles, time constraints, and limited resources, it was not possible for quality checking of all articles to be done independently by two authors. Quality checking of included studies was completed in three phases to ensure a high level of agreement between the authors. In the first phase, a baseline of 20 articles was independently quality checked by DG and EB. During quality checking, the authors did not discuss any of the articles. Once quality checking of the 20 articles was completed and compared, discrepancies were discussed until agreement was reached. If agreement could not be reached, JB was consulted. In the second phase, a further baseline of 20 articles was independently checked and compared after quality checking was completed. Discrepancies were discussed. In the third phase, the remaining articles were independently quality checked by DG (*n* = 34) and EB (*n* = 34).

### Data analysis

In order to avoid bias related to the results of a single study that may be published in multiple publications, the unit of analysis was the study rather than the publication. The included studies were stratified according to substance type, substance use dimension (i.e. intoxication, use, pathological use), SIB dimension, and bivariate or multivariate method of statistical analysis.

In organising the data, we differentiated between studies that specified a particular substance (e.g. alcohol) and studies where the substance investigated was unspecified. We arranged the studies according to the different classes of substances identified in DSM-5 [27]. It is important to note that there have been significant changes between the DSM-IV and DSM-5 with regard to the terminology and classification used for substance related problems. The DSM-5 does not distinguish the diagnoses of substance abuse and dependence as in the DSM-IV. Instead, the DSM-5 offers diagnostic criteria for substance use disorder, including criteria for intoxication, withdrawal, substance/medication-induced disorders, and unspecified substance-induced disorders. A key consideration is that a proportion of studies included in this systematic review were based on the terminology used in the DSM-IV and therefore used terms such as dependence and abuse. This also makes it difficult to integrate or compare studies that use DSM-IV conceptualisations with those that employ DSM-5 concepts.

In this systematic review, substance use dimensions were organised according to intoxication (i.e. drunkenness or use at the time of SIB), use (i.e. any past or present use) and pathological use. The term ‘pathological’ is used here to collectively refer to substance misuse, abuse, addiction, dependence and substance use disorder. The collective term ‘pathological substance use’ was necessary, as the inconsistency in measures of substance use and lack of description used between studies made it difficult to meaningfully distinguish between misuse, abuse, addiction, dependence, and substance use disorder.

We included data for positive, negative, null and unclear associations between substance use dimensions and SIB. The results for bivariate and multivariate statistical analysis were reported separately in the tables and supplementary material so that we could highlight other influencing factors that were controlled for in multivariate studies. Given the heterogeneity of (a) study designs, (b) measures used to measure independent and dependent variables and (c) analysis strategies, it was not possible to conduct a meta-analysis.

## Results

### Overview of the studies

The characteristics of the 108 included articles are described in Table 1, while detail is reported in Additional file 1: Table S3 and Appendix C. With regards to study region, 28 studies were from East Asia and Pacific (EAP), 20 from Europe and Central Asia (ECA), 18 from Latin America and the Caribbean (LAC), 18 from Sub-Saharan Africa (SSA), 12 from Middle East and North Africa (MNA), ten from South Asia (SAS) and two studies from multiple regions.

**Table 1:** Sample characteristics

Characteristic	Number of studies	Citations	%
Study region^a^			
EAP	28	[33, 34, 36, 37, 41, 42, 45–54, 74, 87, 96, 98, 101, 103, 108, 109, 118, 119, 136, 137]	25.9
ECA	20	[60–62, 73, 75, 76, 80, 88, 89, 104, 106, 107, 111, 120, 121, 130, 138–141]	18.5
LAC	18	[38, 43, 44, 56–59, 72, 90, 93, 94, 102, 124–126, 142–144]	16.7
MNA	12	[39, 70, 71, 81, 95, 99, 105, 110, 112, 113, 122, 123]	11.1
SAS	10	[35, 63, 64, 77, 82, 91, 92, 100, 145, 146]	9.25
SSA	18	[32, 40, 65–69, 79, 83–86, 97, 114–117, 147]	16.7
Multiple study regions	2	[55, 78]	1.85
World Bank income group^b^			
LIC	5	[32, 79, 83, 92, 147]	4.63
LMC	19	[35, 40, 50, 52, 60, 63–65, 68, 69, 77, 81, 82, 88, 91, 100, 117, 130, 145]	17.6
UMC	80	[33, 34, 37–39, 41–45, 47–49, 51, 53, 54, 56–59, 61, 62, 66, 70–76, 79, 80, 84–87, 89, 90, 93–99, 101–116, 118–126, 136–144, 146]	74.1
Multiple income groups	4	[36, 46, 55, 78]	3.70
Location			
Urban	18	[32, 34, 38, 52, 56, 58, 61, 63, 66, 88, 93, 96, 102, 115, 124, 130, 136, 145]	16.7
Peri-urban	1	[85]	0.93
Rural	4	[65, 68, 89, 109]	3.70
Both urban and rural	21	[36, 37, 42, 43, 46–48, 53, 54, 64, 77, 81, 87, 99, 105, 108, 114, 119, 137, 141, 147]	19.4
Multiple	3	[78, 86, 110]	2.78
Not available	61	[33, 35, 39–41, 44, 45, 49–51, 55, 57, 59, 60, 62, 67, 69–76, 79, 80, 82–84, 90–92, 94, 95, 97, 98, 100, 101, 103, 104, 106, 107, 111–113, 116–118, 120–123, 125, 126, 138–140, 142–144, 146]	56.5
Study setting			
Clinic based	7	[49, 72, 74, 86, 101, 106, 126]	6.48
Community based	18	[37, 42, 46, 52, 56, 64, 70, 71, 78, 85, 87, 100, 109, 114, 117, 119, 143, 147]	16.7
Hospital based	34	[34, 35, 41, 43, 44, 47, 51, 57, 62, 65, 77, 84, 90, 94–96, 99, 104, 105, 110–113, 120, 121, 123, 125, 136, 138–142, 145]	31.5
School based	27	[33, 36, 39, 40, 45, 48, 50, 53–55, 58–61, 63, 66–69, 80–83, 98, 103, 115, 116]	25
Others	22	[32, 38, 47, 73, 75, 76, 79, 88, 89, 91–93, 97, 102, 107, 108, 122, 124, 130, 137, 144, 145]	20.4
Substance (dimension)			
Intoxication	7	[32, 33, 35, 36, 38–40]	6.5
Use	52	[32, 33, 36, 38–71, 79–86, 98, 101–103, 105–107]	48.2
Pathological use (i.e., Misuse, Abuse, Addiction, Dependence, Disorder)	68	[34, 37, 41, 43–45, 50, 56, 60, 64–66, 70, 72–78, 83, 84, 86–97, 99–101, 104, 108–126, 130, 136–140, 142–147]	63
Suicide (dimension group)			
Suicidal ideation	43	[32, 33, 36–40, 45–48, 50, 52, 55, 56, 58–61, 63, 67–69, 74, 78, 79, 81, 83, 86–91, 95, 100, 105, 117, 122, 126, 143]	39.8
Nonfatal suicidal behaviour	73	[33, 34, 42, 44, 46, 47, 50, 51, 53, 54, 56–60, 62–66, 70–72, 77, 78, 80–89, 93–99, 101–104, 106, 107, 111–125, 136, 138, 139, 141–147]	67.6
Fatal suicide	11	[35, 41, 73, 75, 76, 108–110, 130, 137, 140]	10.2

^a^Study regions: *EAP* East Asia and Pacific, *ECA* Europe and Central Asia, *LAC* Latin America and the Caribbean, *MNA* Middle East and North Africa, *SAS* South Asia, *SSA* Sub-Saharan Africa. ^b^World bank income group: *LIC* Low income country, *LMC* Low-middle-income country, and *UMC* Upper-middle-income country

Of the 108 included studies, 68 (63%) were assessed as high-quality and 23 (21%) were acceptable-quality (Additional file 1: Table S2). Seventeen (16%) were considered low-quality due to issues related to selection bias, attrition, the validity and reliability of exposure measures and whether or not confounding factors were accounted for.

The following substances were investigated: alcohol (*n* = 70), tobacco (*n* = 40), cannabis (*n* = 10), cannabis and mandrax (consumed together) (n = 1), opioids (*n* = 5), sedatives (*n* = 6), stimulants (n = 4), unspecified prescription medication (*n* = 3), inhalants (*n* = 2), and hallucinogens (n = 2). Fifty-two studies did not specify the type of substance investigated.

Table 2 presents a summary of all studies by substance type, substance use dimension, SIB dimension, and assessment for study quality. Tables 3 and 4 summarise all associations between substance use and SIB. The majority of the associations were positive when using bivariate (*n* = 136) and multivariate (*n* = 105) analysis, indicating that substance use was consistently associated with SIB. Fewer associations were null when using bivariate (*n* = 56) or multivariate (*n* = 49) analysis, indicating no significant association. Almost half (*n* = 22) of the null multivariate results were positive in bivariate analysis. Two studies reported negative associations (between non-fatal suicidal behaviour, and alcohol use and cannabis use, respectively) using multivariate analysis. Thirteen of the associations were unclear. Separate consideration of each substance use dimension demonstrated substantial variation among associations.

**Table 2:** Number of Studies by Substance type Specified Dimension, Suicide Dimension, and low-quality status

Substance	Substance use dimension	Suicidal Ideation	NFSB	Fatal suicide
Alcohol	Intoxication	7	2	2
	Use	18 (4 low-quality)	23 (4 low-quality)	0
	Pathological use	11	28 (2 low-quality)	7 (2 low-quality)
Tobacco	Intoxication	0	0	0
	Use	18 (4 low-quality)	22 (3 low-quality)	0
	Pathological use	8 (2 low-quality)	10 (1 low-quality)	0
Cannabis	Intoxication	0	0	0
	Use	5 (2 low-quality)	6 (1 low-quality)	0
	Pathological use	1	2	0
Cannabis and Mandrax	Intoxication	0	0	0
	Use	0	1	0
	Pathological use	0	0	0
Opioid	Intoxication	0	0	0
	Use	1	4	0
	Pathological use	0	1	0
Sedatives	Intoxication	0	0	0
	Use	3 (1 low-quality)	4 (1 low-quality)	0
	Pathological use	1	1	0
Stimulants	Intoxication	0	0	0
	Use	0	4	0
	Pathological use	0	0	0
Unspecified prescription medication	Intoxication	0	0	0
	Use	2	2	0
	Pathological use	0	0	0
Stimulants	Intoxication	0	0	0
	Use	0	4	0
	Pathological use	0	0	0
Inhalants	Intoxication	0	0	0
	Use	0	1	0
	Pathological use	1	1	0
Hallucinogens	Intoxication	0	0	0
	Intoxication	0	2	0
	Use	0	0	0
Unspecified substance	Intoxication	0	1	0
	Use	15 (2 low-quality)	11 (3 low-quality)	0
	Pathological use	10 (1 low-quality)	30 (8 low-quality)	4

**Table 3:** Associations by Substance type Specified Dimension, Suicide Dimension, and Method of Statistical Analysis

Substance use dimension	Suicide dimension	Analysis	Association between substances and suicide				
			Positive	Negative	Null	Unclear	Total
Alcohol							
Alcohol intoxication (*n* =10)	Suicidal ideation	Bivariate	4 [32, 33, 39, 40]	0	0	0	4
		Multivariate	4 [36, 37, 39, 40]	0	2 [32, 38]	0	6
	Non-fatal suicidal behaviour	Bivariate	1 [33]	0	1 [34]	0	2
	Fatal suicide	Bivariate	1 [35]	0	0	0	1
		Multivariate	1 [35]	0	2 [35, 41]	1 [35]	4
Alcohol use (*n* = 33)	Suicidal ideation	Bivariate	11 [33, 46, 49, 50, 55, 56, 60, 61, 67, 69, 143]	0	2 [56, 68]	0	13
		Multivariate	9 [36, 38, 45, 48, 50, 52, 56, 63, 67]	0	2 [56, 68]	0	11
	Non-fatal suicidal behaviour	Bivariate	12 [33, 42, 46, 50, 51, 53, 54, 56, 59, 60, 62, 70]	0	3 [44, 46, 71]	0	15
		Multivariate	8 [43, 47, 50, 57, 58, 63–65]	1 [54]	3 [56, 57, 66]	0	12
Pathological alcohol use (*n* = 37)	Suicidal ideation	Bivariate	5 [56, 83, 87, 90, 143]	0	2 [89, 92]	0	7
		Multivariate	7 [74, 78, 86, 88, 124, 143]	0	3 [56, 83, 100]	0	10
	Non-fatal suicidal behaviour	Bivariate	18 [56, 72, 78, 83, 87, 89, 94–97, 118, 136, 139, 141–145]	0	9 [72, 84, 89, 94, 96, 118, 141, 145, 147]	0	27
Pathological alcohol use (*n* = 37)	Non-fatal suicidal behaviour	Multivariate	11 [66, 78, 86, 88, 89, 93, 94, 124, 136, 138, 143]	0	7 [56, 78, 83, 94, 97, 100, 124]	0	18
	Fatal suicide	Bivariate	5 [37, 73, 75, 76, 140]	0	0	0	5
		Multivariate	2 [130, 137]	0	0	0	2
Tobacco							
Tobacco use (*n* = 29)	Suicidal ideation	Bivariate	10 [46, 49, 50, 55, 56, 59, 67, 69, 81, 83]	0	3 [46, 61, 68]	0	13
		Multivariate	4 [36, 45, 56, 58]	0	5 [38, 50, 68, 83, 86]	2 [50, 79]	13
	Non-fatal suicidal behaviour	Bivariate	13 [33, 36, 46, 50, 53, 54, 56, 59, 67, 70, 80, 81, 83]	0	5 [46, 51, 53, 71, 84]	0	18
		Multivariate	3 [56, 64, 82]	0	5 [58, 64, 83, 85, 86]	1 [50]	9
Pathological tobacco use (*n* = 13)	Suicidal ideation	Bivariate	4 [56, 87, 89, 90]	0	1 [92]	1 [60]	6
		Multivariate	3 [56, 88, 91]	0	1 [88]	0	4
	Non-fatal suicidal behaviour	Bivariate	6 [56, 70, 87, 89, 94, 95]	0	3 [60, 89, 96]	0	9
		Multivariate	4 [56, 88, 89, 93]	0	1 [94]	0	5
Cannabis							
Cannabis use (*n* = 8)	Suicidal ideation	Bivariate	3 [40, 50, 60]		1 [59]	1 [69]	5
	Non-fatal suicidal behaviour	Bivariate	2 [50, 60]	0	2 [59, 70]	0	4
		Multivariate	1 [66]	1 [57]	1 [57]	0	3
Cannabis							
Pathological cannabis use (*n* = 2)	Suicidal ideation	Bivariate	0	0	1 [89]	0	1
	Non-fatal suicidal behaviour	Bivariate	2 [89, 97]	0	1 [89]	0	3
		Multivariate	1 [97]	0	0	0	1
Cannabis and Mandrax (smoked together)							
Cannabis and Mandrax (smoked together) use (*n* = 1)	Non-fatal suicidal behaviour	Multivariate	1 [66]	0	0	0	1
Opioid							
Opioid use (*n* = 4)	Suicidal ideation	Multivariate	1 [98]	0	0	0	1
	Non-fatal suicidal behaviour	Bivariate	0	0	2 [70, 71]	0	2
		Multivariate	2 [66, 98]	0	0	0	2
Pathological opioid use (*n* = 1)	Non-fatal suicidal behaviour	Multivariate	1 [99]	0	0	0	1
Sedatives							
Sedative use (*n* = 5)	Suicidal ideation	Bivariate	0	0	0	1 [60]	1
		Multivariate	2 [52, 98]	0	0	0	2
	Non-fatal suicidal behaviour	Bivariate	1 [60]	0	1 [100]	0	2
		Multivariate	2 [66, 98]	0	0	0	2
Pathological sedative use (*n* = 1)	Suicidal ideation	Bivariate	1 [89]	0	0	0	1
		Multivariate	1 [89]	0	0	0	1
	Non-fatal suicidal behaviour	Bivariate	1 [89]	0	0	0	1
		Multivariate	1 [89]	0	0	0	1
Stimulants							
Stimulant use (*n* = 4)	Non-fatal suicidal behaviour	Bivariate	0	0	1 [70]	0	1
		Multivariate	3 [66, 101, 102]	0	0	0	3
Unspecified prescription medication							
Use of unspecified prescription medication (*n* = 3)	Suicidal ideation	Multivariate	2 [52, 103]	0	0	0	2
	Non-fatal suicidal behaviour	Multivariate	2 [66, 103]	0	0	0	2
Inhalants							
Inhalants use (*n* = 1)	Non-fatal suicidal behaviour	Multivariate	1 [66]	0	0	0	1
Pathological inhalant use (*n* = 1)	Suicidal ideation	Bivariate	0	0	1 [89]	0	1
	Non-fatal suicidal behaviour	Bivariate	1 [89]	0	1 [89]	0	2
		Multivariate	1 [89]	0	0	0	0
Hallucinogens							
Hallucinogens use (*n* = 2)	Non-fatal suicidal behaviour	Bivariate	1 [66]	0	1 [70]	0	2
Total	Suicidal ideation	Bivariate	38	0	11	3	52
		Multivariate	33	0	13	2	48
	Non-fatal suicidal behaviour	Bivariate	58	0	30	0	88
		Multivariate	42	2	17	1	62
	Fatal suicide	Bivariate	6	0	0	0	6
		Multivariate	3	0	2	1	6

**Table 4:** Associations by Unspecified Substance Use Dimension, Suicide Dimension, and Method of Statistical Analysis

Substance use dimension	Suicide dimension	Analysis	Association between substances and suicide				
			Positive	Negative	Null	Unclear	Total
Substance intoxication (*n* = 1)	Non-fatal suicidal behaviour	Bivariate	1 [104]	0	0	0	1
Substance use (*n* = 19)	Suicidal ideation	Bivariate	8 [39, 50, 55, 56, 61, 81, 83, 105]	0	1 [105]	3 [50, 55, 60]	12
		Multivariate	7 [36, 38, 39, 47, 50, 67, 83]	0	4 [32, 56, 58, 67]	1 [50]	12
	Non-fatal suicidal behaviour	Bivariate	7 [33, 50, 56, 81, 83, 106]	0	2 [44, 60]	1 [50]	10
		Multivariate	4 [47, 50, 58, 83]	0	2 [56, 83]	1 [51]	7
Pathological substance use (*n* = 37)	Suicidal ideation	Bivariate	2 [56, 115]	0	1 [92]	0	3
		Multivariate	7 [78, 91, 117, 122, 124, 126, 143]	0	2 [56, 124]	0	9
	Non-fatal suicidal behaviour	Bivariate	15 [34, 56, 72, 77, 78, 94, 96, 104, 111–113, 116, 117, 120, 121]	0	9 [44, 51, 72, 84, 96, 118, 123, 125, 141]	0	24
		Multivariate	8 [56, 65, 78, 96, 117, 119, 122, 126]	0	9 [43, 45, 56, 94, 124, 143]	0	17
	Fatal suicide	Bivariate	1 [109]	0	2 [41, 110]	0	3
		Multivariate	1 [108]	0	0	0	1
Total	Suicidal ideation	Bivariate	10	0	2	3	15
		Multivariate	14	0	6	1	21
	Non-fatal suicidal behaviour	Bivariate	23	0	11	1	35
		Multivariate	12	0	11	1	24
	Fatal suicide	Bivariate	1	0	2	0	3
		Multivariate	1	0	0	0	1

We present a full account of the associations between SIB and substance use below by presenting the data for each type of substance and substance use dimension. In cases where only one or two studies investigated a substance use dimension, the results are elaborated. In cases where three or more studies investigated a substance use dimension, only negative, unclear, or null results that are of interest for the discussion are highlighted.

### Alcohol intoxication

Ten studies investigated the association between alcohol intoxication and SIB. Six studies found a positive association between ever having been drunk and suicidal ideation, while two reported a null association. In Uganda, any drunkenness was associated with suicidal ideation in bivariate but not in multivariate analysis [32]. Of the two studies exploring non-fatal suicidal behaviour, one study [33] found a positive association and one study [34] found a null association. A study from Sri Lanka showed a positive association between drinking alcohol and suicide, only amongst the men in the sample [35].

Half of the studies reporting on alcohol intoxication and SIB focused on adolescents as their target population [36–40] and yielded mixed results based on whether or not the study controlled for comorbid illicit drug use. For example, a population-based study among adolescents between 11 and 15 years old reported a null association between getting drunk in the last month and suicidal ideation after controlling for confounding factors that included unspecified illicit drug use [38]. In contrast, a school-based study among adolescents between 14 and 16 years old reported a positive association between having ever been drunk and suicidal ideation even while controlling for age, sex, worry, loneliness, ever smoked cannabis, and feelings of sadness or hopelessness [40]. The three studies that did not control for illicit drug use found a positive association between alcohol intoxication and suicidal ideation [36, 37, 39].

The studies are predominantly based in the EAP region (i.e. Thailand, China, Philippines, Vietnam, and Taiwan) and are widely spread across study regions for EAP [33, 34, 36, 37, 41] and the SSA region (i.e. Zambia and Uganda) [32, 40]. Individual studies were conducted in the MNA (i.e. Lebanon) [39] and LAC (i.e. Brazil) [38] regions, while no studies were from the ECA region.

### Alcohol use

Thirty-three studies investigated the association between alcohol use and SIB. Seventeen studies found a positive association between ever having consumed or used alcohol and suicidal ideation, and two studies reported a null association. Among the 23 studies that focused on non-fatal suicidal behaviour, 20 reported a positive association and six reported a null association. A Chinese study reported that drinking alcohol before self-harm was positively associated with severity of self-harm in bivariate analysis, yet was inversely related to the severity of the self-harm when using multivariate analysis [42]. Thirty-one of the studies included in this sub-section also included adolescents in their sample, while only two studies focused solely on adults in their sample [43, 44].

The thirty-three studies that form part of this section represent all of the study regions within LMICs but are polarized to only some countries within these regions; EAP (i.e. Philippines, China, Malaysia, Vietnam, Taiwan, Thailand, Kiribati, Samoa, Solomon Islands, Vanuatu) [33, 36, 42, 45–55], LAC (i.e. Mexico, Peru, Brazil) [38, 43, 44, 56–59], ECA (i.e. Kosovo, Turkey) [60–62], SAS (i.e. India) [63, 64], SSA (i.e. Kenya, Namibia, South Africa, Swaziland, Uganda, Zambia, Zimbabwe) [55, 65–69], and MNA (i.e. Iran) [70, 71]. Only two of the studies reporting on the association between alcohol use and SIB made use of a case-control study design [43, 51]. Most of the thirty-three studies were assessed to be of high or acceptable-quality. Eight studies were assessed to be of low-quality [44, 47, 49, 51, 60, 62, 68, 69]. The studies assessed as low-quality were from the ECA, EAP, LAC, and SSA study regions.

### Pathological alcohol use

Thirty-seven studies investigated the association between pathological alcohol use and SIB. Of the studies focusing on suicidal ideation, 10 reported a positive association and five reported a null association. Twenty-three studies found a positive association with non-fatal suicidal behaviour and 15 studies reported a null association. All seven studies focusing on suicide showed positive associations.

Of the thirty-seven studies included in this sub-section, three studies made use of a case-control study design of which one study was assessed to be of low-quality [72], one acceptable-quality [73], and one high-quality [74]. A further two studies made use of an interrupted time-series design where both were assessed to be of low-quality [75, 76]. One other study using a cross-sectional study design was also assessed to be of low-quality [77].

The studies that investigated the association between pathological alcohol use and SIB varied with regard to study region and country. Only one study [78] compared study settings across all study regions by including at least one country from each study region; Brazil (LAC), Bulgaria (ECA), Colombia (LAC), India (SAS), Lebanon (MNA), Mexico LAC, Nigeria (SSA), China (EAP), Romania (ECA), South Africa (SSA), and Ukraine (ECA). Specifically, this study included only adults aged 18 years and older and made use of a cross-sectional study design that was assessed to be of acceptable-quality.

### Tobacco use

Twenty-nine studies explored the association between tobacco use and SIB. Thirteen studies reported a positive association between tobacco use and suicidal ideation, seven reported a null association, and two reported unclear associations. In Botswana, tobacco smoking initiation at younger than 14 years old was associated with suicidal ideation in the past 12 months among girls but not boys [79]. In Kiribati, Samoa, Solomon Islands, and Vanuatu, tobacco smoking initiation younger than 12 years old was associated with both suicidal ideation and non-fatal suicidal behaviour, while tobacco smoking initiation at 12 years and older was not [50]. Thirteen studies reported a positive association between tobacco use and non-fatal suicidal behaviour, and ten studies reported a null association. Two studies [50, 79] reported an unclear association.

Eighteen studies investigating the association between tobacco use and SIB included only adolescents (i.e. 11 to 18 years old) in their sample [36, 38, 45, 50, 54–56, 58, 59, 61, 67–69, 79–83]. The studies in this sub-section predominantly made use of a cross-sectional research design with the exception of one case-control study that was assessed to be of low-quality [51]. A further five studies were assessed to be of low-quality [49, 51, 68, 69, 82, 84]. The twenty-eight studies were mostly conducted in the EAP [33, 36, 45, 46, 49–51, 53–55] and SSA [67–69, 79, 83–86] study regions, while smaller groups of studies were from the ECA [61, 80], LAC [38, 56, 58, 59], MNA [70, 71, 81], and SAS [64, 82] study regions.

### Pathological tobacco use

Thirteen studies explored associations between pathological tobacco use and SIB. Six studies [56, 87–91] reported a positive association with suicidal ideation and two studies [88, 92] reported a null association. One study from Kosovo reported unclear findings, as daily smoking was associated with suicidal ideation among males but not females [60]. Eight studies [56, 70, 87–89, 93–95] reported a positive association with non-fatal suicidal behaviour, while four studies [60, 89, 94, 96] reported a null association.

Most studies were cross-sectional studies [56, 60, 70, 87–93, 95], two were cohort studies [94, 96], and none were case-control or interrupted-time series studies. Only two studies were assessed to be of low-quality [60, 91]. The study regions include EAP [87, 96], ECA [60, 88, 89], LAC [56, 90, 93, 94], MNA [70, 95], and SAS [91, 92], while no studies were from the SSA study region.

### Cannabis use

Eight studies explored the association between cannabis use and SIB. Two studies [50, 60] reported a positive association between cannabis use and suicidal ideation, and two [59, 70] reported a null association. The results of a Zimbabwean study were unclear: cannabis smoking in the past 12 months was associated with suicidal ideation among the total sample and males, but not females [69].

Of the six studies focusing on non-fatal suicidal behaviour, two [50, 60] reported a positive association and two [59, 70] reported a null association when using bivariate analysis. When using multivariate analysis, results were mixed. In South Africa, past month frequency of cannabis use was associated with a higher composite measure of suicide risk [66]. In Mexico, intake of cannabis prior to a suicide attempt was inversely associated with an impulsive or premeditated suicide attempt, while past cannabis use was not associated with any suicide attempt [57].

Seven of the studies on the association between cannabis use and SIB included only adolescents between the ages of 13 and 18 years old, while the remaining two studies included both adolescents and adults in their sample [57, 70]. The studies were predominantly cross-sectional in nature, while one study was a cohort study [57] and none were case-control or interrupted-time series studies. The studies included in this sub-section were from the EAP [50], ECA [60, 61], LAC [57, 59], MNA [70], and SSA [40, 66, 69] study region while no studies were from the SAS study region. Two of the nine studies were assessed to be of low-quality [60, 69].

### Pathological cannabis use

Two studies [89, 97] reported the association between pathological cannabis use and SIB; both explored non-fatal suicidal behaviour and one [89] explored suicidal ideation. In Turkey, cannabis abuse showed a null association with suicidal ideation and suicide attempt, but a positive association with self-harm [89]. A South African study reported a positive association between cannabis use/abuse/dependence and suicide attempts [97]. One study included individuals between the ages of 16 and 22 years [89], while the other study included only adults aged 18 years and older [97]. Both studies were assessed to be of high-quality and made use of a cross-sectional study design.

### Cannabis and mandrax use (consumed together)

One study [66] explored the association between cannabis and mandrax use and SIB. This South African study reported an association between past month frequency of cannabis and mandrax use and higher suicide risk among males from the age of 15 to 18 years old. This study was assessed to be of high-quality and made use of a cross-sectional study design.

### Opioid use

Four studies investigated the association between opioid use and SIB; one [98] focused on suicidal ideation and all four [66, 70, 71, 98] focused on non-fatal suicidal behaviour. In China, lifetime, past year, and past month non-medical use of opioids was associated with suicidal ideation, self-harm, and suicide attempt [98]. In Iran, opioid use was not associated with lifetime suicide attempts [71]. In South Africa, higher past month frequency of opiate use was associated with higher suicide risk [66].

Two studies included males and females between the ages of 12 and 19 years old, while the remaining two studies included individuals from the age of 14 years and older. All four studies in this sub-section made use of a cross-sectional study design and were of a high-quality. The study regions included in this sub-section were MNA [70, 71], SSA [66], and EAP [98], while no studies were from the ECA, LAC, and SAS study regions.

### Pathological opioid use

Only one study [99], from Iran (i.e. MNA study region), explored the association between pathological opioid use and SIB among males and females from the age of 16 to 25 years old. Specifically, the study focused on non-fatal suicidal behaviour: opium dependence was associated with self-immolation. The study was assessed to be of a high-quality and made use of a case-control study design.

### Sedative use

Five studies investigated the association between sedative use and SIB; three [52, 60, 98] focused on suicidal ideation and four [60, 66, 98, 100] focused on non-fatal suicidal behaviour. In Kosovo, tranquilizer use was associated with suicidal ideation for females but not for males, making the association for the overall sample unclear [60]. In China, lifetime, past year, and past month non-medical use of sedatives were associated with self-harm [98].

Three studies included only adolescents in their sample, while one study included individuals between 14 and 65 years old [52], and another included individuals 15 years and older [100]. All five studies employed a cross-sectional study design, where four studies were assessed to be of high-quality and one of low-quality [60]. The study regions in this sub-section included EAP [52, 98], ECA [60], MNA [100], and SSA [66], while no studies were from the LAC or SAS study regions.

### Pathological sedative use

Only one study [89] explored the association between pathological sedative use and SIB, focusing on suicidal ideation and non-fatal suicidal behaviour. This Turkish study showed that tranquilizer abuse was positively associated with suicidal ideation, self-harm and suicide attempt. The individuals included in this sample were between 16 and 22 years old. The study made use of a cross-sectional study design and was assessed to be of high-quality.

### Stimulant use

Four studies investigated the association between stimulant use and SIB. In China, past suicidal behaviour was positively associated with amphetamine-type stimulant (ATS) use [101]. A study from Iran reported a null association between ATS use and suicide attempt [70]. In Brazil, cocaine use was associated with suicide risk [102]. In South Africa, higher past month frequency of cocaine use was associated with suicide risk [66].

All four studies made use of a cross-sectional design and were assessed to be of high-quality. The study regions included EAP [101], LAC [102], MNA [70], and SSA [66], while no studies were from the ECA or SAS study regions.

### Use of unspecified prescription medication

Three studies explored the association between non-medical use of prescription medication and SIB; two [52, 103] focused on suicidal ideation and two [66, 103] focused on non-fatal suicidal behaviour. A Chinese study reported positive associations between having ever considered suicidal behaviour or suicide attempts and non-medical use of prescription pain relief medication [103]. In South Africa, higher past month frequency of over-the-counter medication use was positively associated with non-fatal suicidal behaviour [66]. In Vietnam, any non-medical use of prescription pain relief medication was positively associated with suicidal ideation [52].

All three studies made use of a cross-sectional design and were assessed to be of high-quality. The studies were conducted in the EAP [52, 103] and SSA [66] regions. Two of the studies included only adolescents [66, 103], while one study included individuals of 14 years and older [52].

### Inhalant use

One study [66] explored the association between inhalant use and SIB, focusing on non-fatal suicidal behaviour. In South Africa, higher past month frequency of inhalant use was positively associated with higher suicide risk. The sample included only males between the ages of 15 and 18 years old. The study made use of a cross-sectional design and was assessed to be of high-quality.

### Pathological inhalant use

One study [89] explored the association between pathological inhalant use and SIB, and focused on both suicidal ideation and non-fatal suicidal behaviour. In Turkey, inhalant abuse was positively associated with self-harm, while a null association was found between inhalant abuse and suicidal ideation and suicide attempt. The sample included only males and females between the ages of 16 and 22 years old. The study made use of a cross-sectional design and was assessed to be of high-quality.

### Hallucinogen use

Two studies [66, 70] explored the association between hallucinogen use and SIB; both focused on non-fatal suicidal behaviour. In Iran, a null association was found between hallucinogen use and suicide attempt [70]. In South Africa, higher past month frequency of hallucinogen use was positively associated with higher suicide risk [66]. One study focused only on adolescent males between 15 and 18 years old [66], while the other study included males and females aged 14 years and older. Both studies made use of a cross-sectional design and were considered to be of high-quality.

### Unspecified substance intoxication

One study [104] explored the association between intoxication with an unspecified substance and SIB, and focused on non-fatal suicidal behaviour in a sample of males. In Turkey, substance intoxication was positively associated with self-harm [104]. The study made use of a cross-sectional design and was considered to be of high-quality.

### Use of unspecified substances

Nineteen studies investigated the association between unspecified substance use and SIB; 15 [32, 38, 39, 41, 47, 50, 55, 56, 58, 60, 61, 67, 81, 83, 105] focused on suicidal ideation, 12 [33, 44, 47, 50, 56, 58, 60, 67, 81, 83, 106, 107] focused on non-fatal suicidal behaviours, and none reported on suicide. Eleven studies [36, 38, 39, 47, 50, 55, 56, 61, 81, 83, 105] found a positive association between substance use and suicidal ideation, five [32, 56, 58, 67, 105] reported a null association, and three [36, 55, 60] reported unclear findings. A study from Kosovo did not report findings for the total sample but did report a positive association between use of drugs other than cannabis (amphetamines, hallucinogens, or ecstasy) and suicidal ideation for males but not for females [60]. A study that included samples from the Philippines, China, and Namibia showed that lifetime unspecified drug use was consistently associated with suicide plan across both genders in the Philippines and Namibia, but not China [55].

Ten studies [33, 47, 50, 56, 58, 67, 81, 83, 106, 107] found a positive association between unspecified substance use and non-fatal suicidal behaviour, four [44, 56, 60, 83] reported a null association, and one [50] reported an unclear finding. In Kiribati, Samoa, Solomon Islands, and Vanuatu, substance initiation with one, two, or three substances before 12 years old was not consistently associated with suicidal ideation and suicide attempt [50].

Fifteen studies included adolescent or young adult samples ranging from 11 to 24 years old [32, 33, 36, 38, 39, 50, 55, 56, 58, 60, 61, 67, 81, 83, 106]. Only one study made use of a case-control design [106], no studies used an interrupted-time series design, where the remaining studies were cross-sectional or cohort studies. Three studies were assessed to be of low-quality [44, 47, 60]. No studies in this sub-section were from the SAS study region, while studies were from the ECA [60, 61, 106, 107], EAP [33, 36, 47, 50, 55], LAC [38, 44, 56, 58], MNA [39, 81], and SSA [32, 55, 67, 83] regions.

### Pathological use of unspecified substances

Forty-two studies explored the association between pathological unspecified substance use and SIB. Nine studies reported a positive association with suicidal ideation and three reported a null association. Nineteen studies reported a positive association with non-fatal suicidal behaviour and 16 reported a null association. Two studies [108, 109] reported a positive association with suicide and two [41, 110] reported a null association.

Three studies made use of a case-control study design [51, 72, 111], while no studies used an interrupted-time series design. Nine studies were assessed to be of low-quality [44, 51, 72, 77, 84, 91, 111–113]. The studies were spread across all six study regions; SSA [43, 65, 114–117], EAP [34, 41, 45, 51, 109, 118, 119], ECA [104, 111, 120, 121] SAS [77, 91, 92], MNA [110, 112, 113, 122, 123], and LAC [44, 56, 72, 78, 94, 124–126].

## Discussion

The results from this review demonstrate a consistent positive association between SIB and substance use, substance intoxication, and pathological substance use in studies that use bivariate analysis. These positive associations are somewhat attenuated in studies employing multivariate analysis, but remain predominantly positive. Of the 162 associations reported in the multivariate analysis studies, 55 were null or unclear and only two studies showed negative associations. This pattern of predominantly positive associations was consistent across all substances, substance use dimensions (i.e. intoxication, use, and pathological use), and all SIB dimensions (i.e. suicidal ideation, non-fatal suicidal behaviour, and suicide). These findings support the assertion that substance use is an important risk factor for SIB in LMICs, and should be the target of continued public health policy, research and clinical attention in the effort to advance suicide prevention and reduce the morbidity and mortality associated with suicidal behaviour. A number of points from this review deserve attention and may provide directions for future research in this area.

First, it is significant that most of the included studies focused on alcohol and tobacco (*n* = 78), while illicit drugs and non-medical use of prescription medication received comparatively less attention (*n* = 19). Notable here is the absence of studies on substances such as methamphetamine (also known as Tik) and methcathinone (also known as Kat), which are used in LMICs [127, 128]. This bias in the literature may in part reflect the difficulties researchers face when trying to collect information on illegal activities. Under-reporting of illicit drug use in LMICs is linked to legal constrictions, limited resources and a lack of expertise to screen for drug use [27]. Furthermore, the studies included in this systematic review do not demonstrate the different effects of various substances (e.g. alcohol compared to different types of illicit drug use) as risk factors for SIB. Future research could employ rigorous epidemiological methods that might report on the public health burden and potential mechanisms of the association between a range of substances, substance use dimensions, and SIB. For example, a timeline follow-back methodology may be used to identify potential mechanisms underlying the association between different types of substances and SIB. This information would be helpful to guide policy makers on whether to concentrate their efforts on the reduction of alcohol, tobacco, illicit drug use, or a combination of substances.

Second, the bulk of the evidence comes from studies conducted in UMCs (*n* = 80) and from EAP, ECA, LAC, and SSA regions. This suggests that there is a relative scarcity of studies from LMCs and LICs as well as from regions such as SAS and MNA. This finding is significant given that there are four LICs and one LMC found among the top ten countries with the highest suicide rates in the world. These findings may in part reflect the fact that this review only included studies published in English. Nonetheless, the apparent unequal distribution of studies across LMICs warrants further attention.

Third, most of the literature in this field focuses on suicidal ideation (*n* = 47) and non-fatal suicidal behaviour (*n* = 78); only 11 associations were reported between substance use and suicide. Seven positive associations were reported between alcohol and suicide, no associations were reported for illicit drug use and suicide, and two associations were reported for unspecified substance use and suicide. This suggests that there is a gap in the literature focusing on the association between death by suicide and substance use, particularly illicit drug use. This may in part reflect the reality that in low resource environments it is not routine practice to screen for illicit drugs in mortuaries [127] and that many LMICs lack mortality surveillance systems [1].

Fourth, 11 [35, 50, 55, 60, 68, 69, 76, 79, 95, 98, 106] of the included studies reported findings for men and women separately, while other studies combined them into one group. Globally, evidence suggests that men are at higher risk for substance use and that gender is an important variable in the aetiology of suicidal behaviour [1, 6, 11]. The specific role of gender in the association between substance use and SIB is complex and has not been adequately investigated in the reviewed literature.

Fifth, the majority of studies included in this review were conducted within a risk factor paradigm and have failed to advance the development of theory. This point has been made by other authors in the context of studies about poverty and SIB [129]. It might be helpful if subsequent studies moved beyond simply establishing that substance use is a risk factor for SIB. In this context it is also significant that most studies included in this review employed statistical analyses that only reported on an association between substance use and SIB, without taking potential confounding variables into account. Studies using sophisticated statistical analyses were scarce. One study [130] made use of structural equation modelling, but did not investigate the specific mechanism of action or how exactly substance use increases the risk for SIB. Among studies included in this review, factors that interacted with substance use and SIB were age [35], level of education [38], socioeconomic status [58], feelings of sadness or loneliness [40], parental physical abuse or neglect [32], and comorbid psychiatric disorder [56]. However, the nature of the interaction between these factors is not clear from the available evidence at this time. To answer questions about how substance use interacts with other variables to precipitate SIB, it will be necessary to conduct studies which employ more sophisticated statistical methods and modelling to investigate the interaction between a wider array of variables.

Sixth, most included studies employed a cross-sectional study design. The comparative lack of longitudinal studies in this area is noteworthy. Longitudinal studies could help illuminate how contextual and temporal factors interact with substance use to precipitate SIB. Longitudinal studies will also help generate knowledge on the cumulative effects of substance use over time and how changes in patterns of use may influence SIB. Specifically, studies might focus on factors such as: the role of acute versus chronic substance use; patterns and quantity of substance use; and frequency of substance use. Moreover, suicidal ideation is known to fluctuate over the course of time (sometimes rapidly) making it problematic to simply rely on results from cross-sectional studies [131]. Longitudinal studies may also assist us to understand the progression from suicidal ideation to non-fatal suicidal behaviour and death by suicide among persons who use substances. Such knowledge could contribute to theory building in this area. It is likely that resource constraints in LMICs impede the completion of longitudinal studies in this field. There are a number of reasons why it is difficult to complete longitudinal research in low resource environments, including the high costs of this research, high rates of attrition, and the fact that many people living in low resource environments are highly mobile, making follow-up difficult and expensive [132, 133].

### Study limitations

This systematic review represents an important first step in synthesizing the current literature and planning future public health research in this field. Nonetheless, this study has some limitations. The data may be influenced by publication bias, as studies that report negative or null associations often go unpublished [134]. Some caution is necessary when interpreting the findings from studies assessed as low-quality and where a sub-section consists of only one or two studies (i.e. pathological cannabis use, cannabis and mandrax use, pathological opioid use, pathological sedative use, inhalant use, and hallucinogen use). Excluding qualitative studies limits our ability to understand the mechanism by which substance use relates to SIB as well as the role of sociocultural factors that influence this association. This review only included studies published in English [135], and was limited to studies published between 2006 and 2016.

## Conclusions

The results from this review strongly support the assertion that substance use is associated with increased risk of SIB. This confirms the call from the WHO to focus on substance use as a key element of suicide prevention in LMICs. We assert that the current evidence has the following public health implications in LMICs: (1) substance use is a potentially modifiable risk factor for fatal and non-fatal suicidal behaviour; (2) assessment and management of substance use should be integral to the care of at-risk patients; (3) suicide prevention in LMICs should focus on reducing consumption and hazardous use of alcohol, tobacco, cannabis, opioids, sedatives, stimulants and non-medical use of medications; and (4) more focused research is required in order to better understand the nature of the relationship between substance use and SIB across all types of substances, substance use dimensions, SIB dimensions, and spread throughout all LMICs. It would be helpful if future research focused on providing insight into the nature of this association in a way that permits an understanding of why substance use precipitates SIB among some individuals but not others. We suggest that research needs to be theory-driven and needs to integrate qualitative studies that explore the lived experience of substance use among individuals who engage in SIB.

## Additional file

Additional file 1: Appendix A. Search strategy; Appendix B. Country classification; **Table S1.** Quality assessment criteria; **Table S2.** Study quality; **Table S3.** Characteristics of included studies, by substance type and dimension; Appendix C. Figures to illustrate World Bank region, World Bank income group, study setting, substance type and substance use dimension, suicide dimension and study design. (DOCX 493 kb)

## Abbreviations

ATS: Amphetamine-type stimulant
DSM: Diagnostic and Statistical Manual of Mental Disorders
EAP: East Asia and Pacific
ECA: Europe and Central Asia
HIC: High-income counties
LAC: Latin America and the Caribbean
LIC: Low-income countries
LMC: Lower-middle-income
LMICs: Low- and middle-income countries
MeSH: Medical subject heading
MIC: Middle-income countries
MNA: Middle East and North Africa
PRISMA: Preferred Reporting Items for Systematic Reviews and Meta-Analyses checklist
SAS: South Asia
SIB: Suicidal ideation and behaviour
SSA: Sub-Saharan Africa
UMC: Upper-middle-income countries
WHO: World Health Organisation
