# Supplementary material for: Substance use and suicidal ideation and behaviour in low- and middle-income countries: a systematic review
Source: BMC Public Health. 2018 Apr 24;18:549. doi: 10.1186/s12889-018-5425-6 (PMC5921303; doi:10.1186/s12889-018-5425-6)
Supplement: Supplementary file 1 — Appendix A. Search strategy; Appendix B. Country classification; Table S1. Quality assessment criteria; Table S2. Study quality; Table S3. Characteristics of included studies, by substance type and dimension; Appendix C. Figures to illustrate World Bank region, World Bank income group, study setting, substance type and substance use dimension, suicide dimension and study design. (DOCX 493 kb) [file 12889_2018_5425_MOESM1_ESM.docx]

**Supplementary material**

Appendix A: Search Strategy

Appendix B: Country Classification

Table S1: Quality assessment criteria

Table S2: Study quality

Table S3: Characteristics of included studies, by substance type and dimension (N=108)

Appendix C: Figures to illustrate Word Bank region, World Bank income group, study setting, substance type and substance use dimension, suicide dimension, and study design

**Appendix A: Search strategy**

**Pubmed/MEDLINE (Searched 10 February 2016)**

1. self-injurious behav* [Mesh]

2. ("suicide, assisted"[Mesh] OR “euthanasia” [Mesh] OR “assisted dying”)

3. (“suicide” [Mesh] OR parasuicide* OR "self-killing" OR self-injur* OR self-mutilat* OR self-harm* OR self-immolat* OR self-poison* OR self-drowning OR self-hang* OR "deliberate overdose")

4. (#1 OR #3) NOT #2

5. substance AND (induced OR addiction OR dependen* OR use OR misuse OR withdraw* OR intoxicat*)

6. (alcohol OR caffeine OR cannabis OR cocaine OR crack OR drug OR ecstasy OR hallucinogen OR heroin OR hypnotics OR inhalant OR marijuana OR methamphetamine OR amphetamine OR morphine OR opioid OR opiate OR pain medication OR phencyclidine OR sedative OR solvent OR steroid OR stimulant OR tobacco) AND (substance-induc* OR addict* OR dependen* OR use OR misuse OR withdraw* OR intoxicat* OR chronic use OR acute use OR illicit use OR drug abuse)

7. (substance-related disorder* [Mesh])

8. (Afghanistan OR Benin OR "Burkina Faso" OR Burundi OR "Central African Republic" OR Chad OR Comoros OR "Democratic Republic of the Congo" OR DRC OR Zaire OR Eritrea OR Ethiopia OR Gambia OR Guinea OR Guinea-Bissau OR "Guinea Bissau" OR Haiti OR "Democratic Republic of Korea" OR "North Korea" OR DPRK OR Liberia OR Madagascar OR Malawi OR Mali OR Mozambique OR Nepal OR Niger OR Rwanda OR Senegal OR "Sierra Leone" OR Somalia OR "South Sudan" OR Tanzania OR Togo OR Uganda OR Zimbabwe)

9. (Armenia OR Bangladesh OR Bhutan OR Bolivia OR Cameroon OR Cambodia OR "Cape Verde" OR "Cabo Verde" OR Democratic Republic of the Congo OR DR Congo, DRC, DROC, RDC, Congo-Kinshasa OR Congo OR "Cote d Ivoire" OR "Ivory Coast" OR Djibouti OR Egypt OR "El Salvador" OR Ghana OR Guatemala OR Honduras OR India OR Indonesia OR Kenya OR Kiribati OR Kosovo OR Kyrgyz Republic OR Lao OR Laos OR Lesotho OR Mauritania OR Micronesia OR Moldova OR Mongolia OR Morocco OR Myanmar OR Nicaragua OR Nigeria OR Pakistan OR "Papua New Guinea" OR Philippines OR Samoa OR "Sao Tome" OR Principe OR "Solomon Islands" OR "Sri Lanka" OR Sudan OR Swaziland OR Syria OR "Syrian Arab Republic" OR Tajikistan OR Timor-Leste OR "Timor Leste" OR "East Timor" OR Tonga OR Tunisia OR Ukraine OR Uzbekistan OR Vanuatu OR Vietnam OR "Viet Nam" OR "West Bank" OR Gaza OR Yemen OR Zambia)

10. (Albania OR Algeria OR "American Samoa" OR Angola OR Azerbaijan OR Belarus OR Belize OR Bosnia OR Herzegovina OR Botswana OR Brazil OR Bulgaria OR China OR Colombia OR "Costa Rica" OR Cuba OR Dominica OR "Dominican Republic" OR Ecuador OR Equatorial Guinea OR Fiji OR Gabon OR Georgia OR Grenada OR Guyana OR Iran OR Iraq OR Jamaica OR Jordan OR Kazakhstan OR Lebanon OR Libya OR Macedonia OR Malaysia OR Maldives OR "Marshall Islands" OR Mauritius OR Mexico OR Montenegro OR Namibia OR Palau OR Panama OR Paraguay OR Peru OR Romania OR Russian Federation OR Serbia OR "South Africa" OR "St Lucia" OR "Saint Lucia" OR "St Vincent" OR "Saint Vincent" OR Grenadines OR Suriname OR Thailand OR Turkey OR Turkmenistan OR Tuvalu OR Venezuela)

11. developing countries OR africa OR asia, central OR asia, southeastern OR asia, western OR central america OR south america OR middle east

12. ("developing country" OR "developing countries" OR "low-income country" OR "low-income countries" OR "low income country" OR "low income countries" OR "middle-income country" OR "middle-income countries" OR "middle income country" OR "middle income countries" OR "third world" OR Africa OR "Central Asia" OR "South Asia" OR "Southeast Asia" OR "South-East Asia" OR "Central America" OR "Latin America" OR "South America" OR "Middle East")

13. #5 OR #6 OR #7

14. #8 OR #9 OR #10 OR #11 OR #12

15. #4 AND #13 AND #14

16. limit 15 to yr="2006 -Current"

**CINHAL Plus (EBSCO) (Searched 10 February 2016)**

1. (MH “Suicide +”)
2. (MH "Suicide, Assisted") OR (MH "Euthanasia+") OR “assisted dying”
3. (parasuicide* OR "self-killing" OR self-injur* OR self-mutilat* OR self-harm* OR self-immolat* OR self-poison* OR self-drowning OR self-hang* OR "deliberate overdose")
4. S1 NOT S2
5. S3 NOT S2
6. substance NEAR (induced OR addiction OR dependen* OR use OR misuse OR withdraw* OR intoxicat*)
7. (alcohol OR caffeine OR cannabis OR cocaine OR crack OR drug OR ecstasy OR hallucinogen OR heroin OR hypnotics OR inhalant OR marijuana OR methamphetamine OR amphetamine OR morphine OR opioid OR opiate OR pain medication OR phencyclidine OR sedative OR solvent OR steroid OR stimulant OR tobacco) AND (substance-induc* OR addict* OR dependen* OR use OR misuse OR withdraw* OR intoxicat* OR chronic use OR acute use OR illicit use OR drug abuse)
8. (MH "Substance Use Disorders+")
9. (Afghanistan OR Benin OR "Burkina Faso" OR Burundi OR "Central African Republic" OR Chad OR Comoros OR "Democratic Republic of the Congo" OR DRC OR Zaire OR Eritrea OR Ethiopia OR Gambia OR Guinea OR Guinea-Bissau OR "Guinea Bissau" OR Haiti OR "Democratic Republic of Korea" OR "North Korea" OR DPRK OR Liberia OR Madagascar OR Malawi OR Mali OR Mozambique OR Nepal OR Niger OR Rwanda OR Senegal OR "Sierra Leone" OR Somalia OR "South Sudan" OR Tanzania OR Togo OR Uganda OR Zimbabwe)
10. (Armenia OR Bangladesh OR Bhutan OR Bolivia OR Cameroon OR Cambodia OR "Cape Verde" OR "Cabo Verde" OR Democratic Republic of the Congo OR DR Congo, DRC, DROC, RDC, Congo-Kinshasa OR Congo OR "Cote d Ivoire" OR "Ivory Coast" OR Djibouti OR Egypt OR "El Salvador" OR Ghana OR Guatemala OR Honduras OR India OR Indonesia OR Kenya OR Kiribati OR Kosovo OR Kyrgyz Republic OR Lao OR Laos OR Lesotho OR Mauritania OR Micronesia OR Moldova OR Mongolia OR Morocco OR Myanmar OR Nicaragua OR Nigeria OR Pakistan OR "Papua New Guinea" OR Philippines OR Samoa O7R "Sao Tome" OR Principe OR "Solomon Islands" OR "Sri Lanka" OR Sudan OR Swaziland OR Syria OR "Syrian Arab Republic" OR Tajikistan OR Timor-Leste OR "Timor Leste" OR "East Timor" OR Tonga OR Tunisia OR Ukraine OR Uzbekistan OR Vanuatu OR Vietnam OR "Viet Nam" OR "West Bank" OR Gaza OR Yemen OR Zambia)
11. (Albania OR Algeria OR "American Samoa" OR Angola OR Azerbaijan OR Belarus OR Belize OR Bosnia OR Herzegovina OR Botswana OR Brazil OR Bulgaria OR China OR Colombia OR "Costa Rica" OR Cuba OR Dominica OR "Dominican Republic" OR Ecuador OR Equatorial Guinea OR Fiji OR Gabon OR Georgia OR Grenada OR Guyana OR Iran OR Iraq OR Jamaica OR Jordan OR Kazakhstan OR Lebanon OR Libya OR Macedonia OR Malaysia OR Maldives OR "Marshall Islands" OR Mauritius OR Mexico OR Montenegro OR Namibia OR Palau OR Panama OR Paraguay OR Peru OR Romania OR Russian Federation OR Serbia OR "South Africa" OR "St Lucia" OR "Saint Lucia" OR "St Vincent" OR "Saint Vincent" OR Grenadines OR Suriname OR Thailand OR Turkey OR Turkmenistan OR Tuvalu OR Venezuela)
12. (MH "Developing Countries") OR (MH "Africa+") OR (MH "Asia, Central+") OR (MH "Asia, Southeastern+") OR (MH "Asia, Western+") OR (MH "Central America+") OR (MH "South America+") OR (MH "Middle East+")
13. ("developing country" OR "developing countries" OR "low-income country" OR "low-income countries" OR "low income country" OR "low income countries" OR "middle-income country" OR "middle-income countries" OR "middle income country" OR "middle income countries" OR "third world" OR Africa OR "Central Asia" OR "South Asia" OR "Southeast Asia" OR "South-East Asia" OR "Central America" OR "Latin America" OR "South America" OR "Middle East")
14. S4 OR S5
15. S6 OR S7 OR S8
16. S9 OR S10 OR S11 OR S12 OR S13
17. S14 AND S15 AND S16
18. Limit 18 to yr=”2006-Current

**DARE (Database of Abstracts of Reviews of Effectiveness) (The Cochrane Library)**

1. MeSH DESCRIPTOR Self-Injurious Behavior EXPLODE ALL TREES

2. MeSH DESCRIPTOR Suicide, Assisted

3. MeSH DESCRIPTOR Euthanasia EXPLODE 1

4. #2 OR #3

5. #1 NOT #4

6. (parasuicide* OR "self-killing" OR self-injur* OR self-mutilat* OR self-harm* OR self-immolat* OR self-poison* OR self-drowning OR self-hang* OR "deliberate overdose")

7. (("assisted suicide" or euthanasia or "assisted dying")) IN DARE

8.#6 NOT #7

9.#5 OR #8

10. (substance AND (induced OR addiction OR dependen* OR use OR misuse OR withdraw* OR intoxicat*)) IN DARE

11. ((alcohol OR caffeine OR cannabis OR cocaine OR crack OR drug OR ecstasy OR hallucinogen OR heroin OR hypnotics OR inhalant OR marijuana OR methamphetamine OR amphetamine OR morphine OR opioid OR opiate OR pain medication OR phencyclidine OR sedative OR solvent OR steroid OR stimulant OR tobacco) AND (substance-induc* OR addict* OR dependen* OR use OR misuse OR withdraw* OR intoxicat* OR chronic use OR acute use OR illicit use OR drug abuse)) IN DARE

12. ((substance-related disorder*)) IN DARE

13. ((Afghanistan OR Benin OR "Burkina Faso" OR Burundi OR "Central African Republic" OR Chad OR Comoros OR "Democratic Republic of the Congo" OR DRC OR Zaire OR Eritrea OR Ethiopia OR Gambia OR Guinea OR Guinea-Bissau OR "Guinea Bissau" OR Haiti OR "Democratic Republic of Korea" OR "North Korea" OR DPRK OR Liberia OR Madagascar OR Malawi OR Mali OR Mozambique OR Nepal OR Niger OR Rwanda OR Senegal OR "Sierra Leone" OR Somalia OR "South Sudan" OR Tanzania OR Togo OR Uganda OR Zimbabwe)) IN DARE

14. ((Armenia OR Bangladesh OR Bhutan OR Bolivia OR Cameroon OR Cambodia OR "Cape Verde" OR "Cabo Verde" OR Democratic Republic of the Congo OR DR Congo, DRC, DROC, RDC, Congo-Kinshasa OR Congo OR "Cote d Ivoire" OR "Ivory Coast" OR Djibouti OR Egypt OR "El Salvador" OR Ghana OR Guatemala OR Honduras OR India OR Indonesia OR Kenya OR Kiribati OR Kosovo OR Kyrgyz Republic OR Lao OR Laos OR Lesotho OR Mauritania OR Micronesia OR Moldova OR Mongolia OR Morocco OR Myanmar OR Nicaragua OR Nigeria OR Pakistan OR "Papua New Guinea" OR Philippines OR Samoa OR "Sao Tome" OR Principe OR "Solomon Islands" OR "Sri Lanka" OR Sudan OR Swaziland OR Syria OR "Syrian Arab Republic" OR Tajikistan OR Timor-Leste OR "Timor Leste" OR "East Timor" OR Tonga OR Tunisia OR Ukraine OR Uzbekistan OR Vanuatu OR Vietnam OR "Viet Nam" OR "West Bank" OR Gaza OR Yemen OR Zambia)) IN DARE

15. ((Albania OR Algeria OR "American Samoa" OR Angola OR Azerbaijan OR Belarus OR Belize OR Bosnia OR Herzegovina OR Botswana OR Brazil OR Bulgaria OR China OR Colombia OR "Costa Rica" OR Cuba OR Dominica OR "Dominican Republic" OR Ecuador OR Equatorial Guinea OR Fiji OR Gabon OR Georgia OR Grenada OR Guyana OR Iran OR Iraq OR Jamaica OR Jordan OR Kazakhstan OR Lebanon OR Libya OR Macedonia OR Malaysia OR Maldives OR "Marshall Islands" OR Mauritius OR Mexico OR Montenegro OR Namibia OR Palau OR Panama OR Paraguay OR Peru OR Romania OR Russian Federation OR Serbia OR "South Africa" OR "St Lucia" OR "Saint Lucia" OR "St Vincent" OR "Saint Vincent" OR Grenadines OR Suriname OR Thailand OR Turkey OR Turkmenistan OR Tuvalu OR Venezuela)) IN DARE

16. (developing countries OR africa OR asia, central OR asia, southeastern OR asia, western OR central america OR south america OR middle ) IN DARE

17. (("developing country" OR "developing countries" OR "low-income country" OR "low-income countries" OR "low income country" OR "low income countries" OR "middle-income country" OR "middle-income countries" OR "middle income country" OR "middle income countries" OR "third world" OR Africa OR "Central Asia" OR "South Asia" OR "Southeast Asia" OR "South-East Asia" OR "Central America" OR "Latin America" OR "South America" OR "Middle East")) IN DARE

18. #10 OR #11 OR #12

19. #13 OR #14 OR #15 OR #16 OR #17

20. #9 AND #18 AND #19

21. limit 20 to yr="2006 -Current"

**Web of Science (Web of Science) (Searched 10 February 2016)**

1. (TS=(suicide* OR parasuicid* OR "self-killing" OR self-injur* OR self-mutilat* OR self-harm* OR self-immolat* OR self-poison* OR self-drowning OR self-hang* OR "deliberate overdose")) OR (TI=( suicide* OR parasuicid* OR "self-killing" OR self-injur* OR self-mutilat* OR self-harm* OR self-immolat* OR self-poison* OR self-drowning OR self-hang* OR "deliberate overdose"))

2. (TS=(euthanasia OR "assisted suicide" OR "assisted dying")) OR (TI=(euthanasia OR "assisted suicide" OR "assisted dying"))

3. #1 NOT #2

4. (TS=(substance) AND TS=(induced OR addiction OR dependen* OR use OR misuse OR withdraw* OR intoxicat*)) OR (TI=(substance) AND TI=(induced OR addiction OR dependen* OR use OR misuse OR withdraw* OR intoxicat*))

5. (TS=(alcohol OR caffeine OR cannabis OR cocaine OR crack OR drug OR ecstasy OR hallucinogen OR heroin OR hypnotics OR inhalant OR marijuana OR methamphetamine OR amphetamine OR morphine OR opioid OR opiate OR pain medication OR phencyclidine OR sedative OR solvent OR steroid OR stimulant OR tobacco) AND TS=(substance-induc* OR addict* OR dependen* OR use OR misuse OR withdraw* OR intoxicat* OR chronic use OR acute use OR illicit use OR drug abuse)) OR (TI=(alcohol OR caffeine OR cannabis OR cocaine OR crack OR drug OR ecstasy OR hallucinogen OR heroin OR hypnotics OR inhalant OR marijuana OR methamphetamine OR amphetamine OR morphine OR opioid OR opiate OR pain medication OR phencyclidine OR sedative OR solvent OR steroid OR stimulant OR tobacco) AND TI=(substance-induc* OR addict* OR dependen* OR use OR misuse OR withdraw* OR intoxicat* OR chronic use OR acute use OR illicit use OR drug abuse))

6. (TS=(substance-related disorder*)) OR (TI=(substance-related disorder*))

7. (TS=(Afghanistan OR Benin OR "Burkina Faso" OR Burundi OR "Central African Republic" OR Chad OR Comoros OR "Democratic Republic of the Congo" OR DRC OR Zaire OR Eritrea OR Ethiopia OR Gambia OR Guinea OR Guinea-Bissau OR "Guinea Bissau" OR Haiti OR "Democratic Republic of Korea" OR "North Korea" OR DPRK OR Liberia OR Madagascar OR Malawi OR Mali OR Mozambique OR Nepal OR Niger OR Rwanda OR Senegal OR "Sierra Leone" OR Somalia OR "South Sudan" OR Tanzania OR Togo OR Uganda OR Zimbabwe)) OR (TI=(Afghanistan OR Benin OR "Burkina Faso" OR Burundi OR "Central African Republic" OR Chad OR Comoros OR "Democratic Republic of the Congo" OR DRC OR Zaire OR Eritrea OR Ethiopia OR Gambia OR Guinea OR Guinea-Bissau OR "Guinea Bissau" OR Haiti OR "Democratic Republic of Korea" OR "North Korea" OR DPRK OR Liberia OR Madagascar OR Malawi OR Mali OR Mozambique OR Nepal OR Niger OR Rwanda OR Senegal OR "Sierra Leone" OR Somalia OR "South Sudan" OR Tanzania OR Togo OR Uganda OR Zimbabwe))

8. (TS=(Armenia OR Bangladesh OR Bhutan OR Bolivia OR Cameroon OR Cambodia OR "Cape Verde" OR "Cabo Verde" OR Democratic Republic of the Congo OR DR Congo, DRC, DROC, RDC, Congo-Kinshasa OR Congo OR "Cote d Ivoire" OR "Ivory Coast" OR Djibouti OR Egypt OR "El Salvador" OR Ghana OR Guatemala OR Honduras OR India OR Indonesia OR Kenya OR Kiribati OR Kosovo OR Kyrgyz Republic OR Lao OR Laos OR Lesotho OR Mauritania OR Micronesia OR Moldova OR Mongolia OR Morocco OR Myanmar OR Nicaragua OR Nigeria OR Pakistan OR "Papua New Guinea" OR Philippines OR Samoa OR "Sao Tome" OR Principe OR "Solomon Islands" OR "Sri Lanka" OR Sudan OR Swaziland OR Syria OR "Syrian Arab Republic" OR Tajikistan OR Timor-Leste OR "Timor Leste" OR "East Timor" OR Tonga OR Tunisia OR Ukraine OR Uzbekistan OR Vanuatu OR Vietnam OR "Viet Nam" OR "West Bank" OR Gaza OR Yemen OR Zambia)) OR (TI=(Armenia OR Bangladesh OR Bhutan OR Bolivia OR Cameroon OR Cambodia OR "Cape Verde" OR "Cabo Verde" OR Democratic Republic of the Congo OR DR Congo, DRC, DROC, RDC, Congo-Kinshasa OR Congo OR "Cote d Ivoire" OR "Ivory Coast" OR Djibouti OR Egypt OR "El Salvador" OR Ghana OR Guatemala OR Honduras OR India OR Indonesia OR Kenya OR Kiribati OR Kosovo OR Kyrgyz Republic OR Lao OR Laos OR Lesotho OR Mauritania OR Micronesia OR Moldova OR Mongolia OR Morocco OR Myanmar OR Nicaragua OR Nigeria OR Pakistan OR "Papua New Guinea" OR Philippines OR Samoa OR "Sao Tome" OR Principe OR "Solomon Islands" OR "Sri Lanka" OR Sudan OR Swaziland OR Syria OR "Syrian Arab Republic" OR Tajikistan OR Timor-Leste OR "Timor Leste" OR "East Timor" OR Tonga OR Tunisia OR Ukraine OR Uzbekistan OR Vanuatu OR Vietnam OR "Viet Nam" OR "West Bank" OR Gaza OR Yemen OR Zambia))

9. (TS=(Albania OR Algeria OR "American Samoa" OR Angola OR Azerbaijan OR Belarus OR Belize OR Bosnia OR Herzegovina OR Botswana OR Brazil OR Bulgaria OR China OR Colombia OR "Costa Rica" OR Cuba OR Dominica OR "Dominican Republic" OR Ecuador OR Equatorial Guinea OR Fiji OR Gabon OR Georgia OR Grenada OR Guyana OR Iran OR Iraq OR Jamaica OR Jordan OR Kazakhstan OR Lebanon OR Libya OR Macedonia OR Malaysia OR Maldives OR "Marshall Islands" OR Mauritius OR Mexico OR Montenegro OR Namibia OR Palau OR Panama OR Paraguay OR Peru OR Romania OR Russian Federation OR Serbia OR "South Africa" OR "St Lucia" OR "Saint Lucia" OR "St Vincent" OR "Saint Vincent" OR Grenadines OR Suriname OR Thailand OR Turkey OR Turkmenistan OR Tuvalu OR Venezuela)) OR (TI=(Albania OR Algeria OR "American Samoa" OR Angola OR Azerbaijan OR Belarus OR Belize OR Bosnia OR Herzegovina OR Botswana OR Brazil OR Bulgaria OR China OR Colombia OR "Costa Rica" OR Cuba OR Dominica OR "Dominican Republic" OR Ecuador OR Equatorial Guinea OR Fiji OR Gabon OR Georgia OR Grenada OR Guyana OR Iran OR Iraq OR Jamaica OR Jordan OR Kazakhstan OR Lebanon OR Libya OR Macedonia OR Malaysia OR Maldives OR "Marshall Islands" OR Mauritius OR Mexico OR Montenegro OR Namibia OR Palau OR Panama OR Paraguay OR Peru OR Romania OR Russian Federation OR Serbia OR "South Africa" OR "St Lucia" OR "Saint Lucia" OR "St Vincent" OR "Saint Vincent" OR Grenadines OR Suriname OR Thailand OR Turkey OR Turkmenistan OR Tuvalu OR Venezuela))

10. (TS=(developing countries OR africa OR asia, central OR asia , southeastern OR asia, western OR central america OR south america OR middle east)) OR (TI=(developing countries OR africa OR asia, central OR asia , southeastern OR asia, western OR central america OR south america OR middle east))

11. (TS=("developing country" OR "developing countries" OR "low-income country" OR "low-income countries" OR "low income country" OR "low income countries" OR "middle-income country" OR "middle-income countries" OR "middle income country" OR "middle income countries" OR "third world" OR Africa OR "Central Asia" OR "South Asia" OR "Southeast Asia" OR "South-East Asia" OR "Central America" OR "Latin America" OR "South America" OR "Middle East")) OR (TI=("developing country" OR "developing countries" OR "low-income country" OR "low-income countries" OR "low income country" OR "low income countries" OR "middle-income country" OR "middle-income countries" OR "middle income country" OR "middle income countries" OR "third world" OR Africa OR "Central Asia" OR "South Asia" OR "Southeast Asia" OR "South-East Asia" OR "Central America" OR "Latin America" OR "South America" OR "Middle East"))

12. 4 OR 5 OR 6

13. 7 OR 8 OR 9 OR 10 OR 11

14. #3 AND #12 AND #13

15. limit 14 to yr="2006-2016"

**PsycINFO (OvidSP) (Searched 15 February 2016)**

1. self destructive behavior

2. euthanasia OR assisted suicide

3. 1 NOT 2

4. (suicid* OR parasuicid* OR "self-killing" OR self-injur* OR self-mutilat* OR self-harm* OR self-immolat* OR self-poison* OR defenestrat* OR self-drowning OR self-hang* OR "deliberate overdose")

5. ("assisted suicide" OR euthanasia OR "assisted dying")

6. 4 NOT 5

7. substance AND (induced OR addiction OR dependen* OR use OR misuse OR withdraw* OR intoxicat*)

8.(alcohol OR caffeine OR cannabis OR cocaine OR crack OR drug OR ecstasy OR hallucinogen OR heroin OR hypnotics OR inhalant OR marijuana OR methamphetamine OR amphetamine OR morphine OR opioid OR opiate OR pain medication OR phencyclidine OR sedative OR solvent OR steroid OR stimulant OR tobacco) AND (substance-induc* OR addict* OR dependen* OR use OR misuse OR withdraw* OR intoxicat* OR chronic use OR acute use OR illicit use OR drug abuse)

9.substance use disorder

10. (Afghanistan OR Benin OR "Burkina Faso" OR Burundi OR "Central African Republic" OR Chad OR Comoros OR "Democratic Republic of the Congo" OR DRC OR Zaire OR Eritrea OR Ethiopia OR Gambia OR Guinea OR Guinea-Bissau OR "Guinea Bissau" OR Haiti OR "Democratic Republic of Korea" OR "North Korea" OR DPRK OR Liberia OR Madagascar OR Malawi OR Mali OR Mozambique OR Nepal OR Niger OR Rwanda OR Senegal OR "Sierra Leone" OR Somalia OR "South Sudan" OR Tanzania OR Togo OR Uganda OR Zimbabwe)

11. (Armenia OR Bangladesh OR Bhutan OR Bolivia OR Cameroon OR Cambodia OR "Cape Verde" OR "Cabo Verde" OR Democratic Republic of the Congo OR DR Congo, DRC, DROC, RDC, Congo-Kinshasa OR Congo OR "Cote d Ivoire" OR "Ivory Coast" OR Djibouti OR Egypt OR "El Salvador" OR Ghana OR Guatemala OR Honduras OR India OR Indonesia OR Kenya OR Kiribati OR Kosovo OR Kyrgyz Republic OR Lao OR Laos OR Lesotho OR Mauritania OR Micronesia OR Moldova OR Mongolia OR Morocco OR Myanmar OR Nicaragua OR Nigeria OR Pakistan OR "Papua New Guinea" OR Philippines OR Samoa OR "Sao Tome" OR Principe OR "Solomon Islands" OR "Sri Lanka" OR Sudan OR Swaziland OR Syria OR "Syrian Arab Republic" OR Tajikistan OR Timor-Leste OR "Timor Leste" OR "East Timor" OR Tonga OR Tunisia OR Ukraine OR Uzbekistan OR Vanuatu OR Vietnam OR "Viet Nam" OR "West Bank" OR Gaza OR Yemen OR Zambia)

12. (Albania OR Algeria OR "American Samoa" OR Angola OR Azerbaijan OR Belarus OR Belize OR Bosnia OR Herzegovina OR Botswana OR Brazil OR Bulgaria OR China OR Colombia OR "Costa Rica" OR Cuba OR Dominica OR "Dominican Republic" OR Ecuador OR Equatorial Guinea OR Fiji OR Gabon OR Georgia OR Grenada OR Guyana OR Iran OR Iraq OR Jamaica OR Jordan OR Kazakhstan OR Lebanon OR Libya OR Macedonia OR Malaysia OR Maldives OR "Marshall Islands" OR Mauritius OR Mexico OR Montenegro OR Namibia OR Palau OR Panama OR Paraguay OR Peru OR Romania OR Russian Federation OR Serbia OR "South Africa" OR "St Lucia" OR "Saint Lucia" OR "St Vincent" OR "Saint Vincent" OR Grenadines OR Suriname OR Thailand OR Turkey OR Turkmenistan OR Tuvalu OR Venezuela)

13. developing countries OR africa OR asia, central OR asia, southeastern OR asia, western OR central america OR south america OR middle east

14. ("developing country" OR "developing countries" OR "low-income country" OR "low-income countries" OR "low income country" OR "low income countries" OR "middle-income country" OR "middle-income countries" OR "middle income country" OR "middle income countries" OR "third world" OR Africa OR "Central Asia" OR "South Asia" OR "Southeast Asia" OR "South-East Asia" OR "Central America" OR "Latin America" OR "South America" OR "Middle East")

15. S3 OR S6

16. S7 OR S8 OR S9

17. S10 OR S11 OR S12 OR S13 OR S14

18. S15 AND S16 AND S17

19. Limit 18 to yr=”2006-Current

**Appendix B: Country classification (list of low-income and middle-income countries)**

**Low-income countries**

Afghanistan, Benin, Burkina Faso, Burundi, Central African Republic, Chad, Comoros, Congo, Dem. Rep., Eritrea, Ethiopia, Gambia, The, Guinea, Guinea-Bissau, Haiti, Korea, Dem Rep., Liberia, Madagascar, Malawi, Mali, Mozambique, Nepal, Niger, Rwanda, Senegal, Sierra Leone, Somalia, South Sudan, Tanzania, Togo, Uganda, Zimbabwe.

**Lower-middle-income countries**

Armenia, Bangladesh, Bhutan, Bolivia, Cabo Verde, Cambodia, Cameroon, Congo, Rep., Côte d'Ivoire, Djibouti, Egypt, Arab Rep., El Salvador, Ghana, Guatemala, Honduras, India, Indonesia, Kenya, Kiribati, Kosovo, Kyrgyz Republic, Lao PDR, Lesotho, Mauritania, Micronesia, Fed. Sts., Moldova, Mongolia, Morocco, Myanmar, Nicaragua, Nigeria, Pakistan, Papua New Guinea, Philippines, Samoa, São Tomé and Principe, Solomon Islands, Sri Lanka, Sudan, Swaziland, Syrian Arab Republic, Tajikistan, Timor-Leste, Tonga, Tunisia, Ukraine, Uzbekistan, Vanuatu, Vietnam, West Bank and Gaza, Yemen, Rep., Zambia.

**Upper-middle-income country**

Albania, Algeria, American Samoa, Angola, Argentina, Azerbaijan, Belarus, Belize, Bosnia and Herzegovina, Botswana, Brazil, Bulgaria, China, Colombia, Costa Rica, Cuba, Dominica, Dominican Republic, Ecuador, Equatorial Guinea, Fiji, Gabon, Georgia, Grenada, Guyana, Iran, Islamic Rep., Iraq, Jamaica, Jordan, Kazakhstan, Lebanon, Libya, Macedonia, FYR, Malaysia, Maldives, Marshall Islands, Mauritius, Mexico, Montenegro, Namibia, Palau, Panama, Paraguay, Peru, Romania, Russian Federation, Serbia, South Africa, St. Lucia, St. Vincent and the Grenadines, Suriname, Thailand, Turkey, Turkmenistan, Tuvalu, Venezuela, RB.

**Table S1 Quality assessment criteria**

| Study design | Criteria |
| --- | --- |
| All study designs | Appropriate research question, valid results, generalizable results. |
| Cross-sectional | Participation rate, clearly defined outcomes, validity and reliability of exposure and outcome measures, and identification of  potential confounders and confidence intervals. |
| Cohort study; interrupted-time series | Comparable baseline, participation rate, outcome presents at baseline, losses to follow-up, impact of losses to follow-up, clearly  defined outcomes, blind outcome assessment, acknowledgment of impact of non-blind assessment, reliable exposure assessment,  validity of outcome assessment, validity of exposure measure, identification of potential confounders and confidence intervals, and  use of control group. |
| Case-control study | Comparable case and controls, same exclusion criteria, participation rate, similarities at baseline, clear case and control definitions,  blind outcome assessment, reliability of exposure measure, and identification of potential confounders and confidence intervals. |
| Overall ratings |  |
| High-quality (++) | The majority of criteria are met with little or no risk of bias. |
| Acceptable-quality (+) | The majority of criteria are met with some risk of bias. |
| Low-quality (-) | The majority of criteria are not met with significant risk of bias. |

**Table S2** Study quality

|  | Low-quality studies | Acceptable-quality studies | High-quality studies |
| --- | --- | --- | --- |
| Cross-sectional study (n=83) | 11 [47, 49, 60, 62, 68, 69, 77, 82, 84, 91, 113] | 15 [33, 37, 39, 59, 61, 67, 78, 80, 87, 110, 122, 112, 115, 130, 145] | 57 [32, 36, 38, 40, 42, 45, 46, 48, 50, 52, 53, 54, 55, 56, 58, 63, 64, 65, 66, 70,  71, 79, 81, 83, 85, 86, 88, 89, 90, 92,  93, 95, 97, 98, 100, 101, 103, 102, 104, 105, 107, 116, 117, 118, 119, 120, 121, 123, 124, 125, 126, 136, 138, 142, 143, 146, 147] |
| Cohort study (n=13) | 1 [44] | 6 [35, 57, 94, 137, 140, 141] | 6 [34, 41, 96, 114, 139, 144] |
| Case-control study (n = 10) | 3 [51, 72, 111] | 2 [43, 73] | 5 [74, 99, 106, 108, 109] |
| Interrupted-time series (n=2) | 2 [75, 76] | 0 | 0 |
| Total | 17 | 23 | 68 |

**Table S3 Characteristics of included studies, by substance type and dimension (N=108)**

| **Substance investigated** | **Substance use dimension** | | **Authors / study** | **Study (setting)** | | **^*^Target population: adult vs adolescent (age)** | | **Study design (sample size)** | | **Gender (%)** | | **Suicide dimension** | | **Statistical analysis** | | **Finding** | | **Association** | | **Study quality** | |
| --- | --- | --- | --- | --- | --- | --- | --- | --- | --- | --- | --- | --- | --- | --- | --- | --- | --- | --- | --- | --- | --- |
| **Alcohol** |  | |  |  | |  | |  | |  | |  | |  | |  | |  | |  | |
| **Alcohol** | Intoxication | | Eddleston, Gunnell et al, 2009 [35] | Sri Lanka  Hospital based | | N/A | | Cohort study | | Male= 55 (76.4%) & female=17 (23.6%) | | Fatal suicide | | Bivariate | | Median alcohol concentration in patients who died (0·94 g l-1·94 mg dl-1, IQR 0·52–1·30) was higher compared to survivors (0·0 g l-1, IQR 0.00–1·04; Mann– Whitney p=0·018). | | Positive | | + | |
|  |  |  |  |  |  |  |  |  |  |  |  |  |  | Multivariate | | Risk of death by suicide was associated with drinking alcohol (OR=4·3, 95% CI 1·2-16·4, p<0·05).  Remained associated when only men were studied (OR=4·8, 95% CI 1·2-19·3, NO p-value); amongst women, there was only one death and she had not consumed alcohol. | | Unclear | |  |  |
|  |  |  |  |  |  |  |  |  |  |  |  |  |  | Multivariate | | The risk of death by suicide associated with alcohol ingestion was weakened by controlling for age (aOR=4·1, 95% CI 1·0-17·8, p<0·05). | | Positive | |  |  |
|  |  |  |  |  |  |  |  |  |  |  |  |  |  | Multivariate | | The risk of death associated with alcohol ingestion was not significant by additionally controlling for dimethoate (OR=0·3, 95% CI 0·0-8·8, p>0·05). This indicates that the deaths were not due to the direct toxic effects of alcohol. | | Null | |  |  |
|  |  | |  |  | |  | |  | |  | |  | |  | |  | |  | |  | |
| **Alcohol** | Intoxication | Mahfoud et al, 2011 [39] | | Lebanon  School based | Adolescents (11 to 16 years old) | | Cross-sectional study | | Male (47.7%) female (52.3%) | | Suicidal ideation | | Bivariate | | Intoxication (OR=2·61, 95% CI 1·99-3·42, p<0·05) was associated with suicidal ideation. | | Positive | | + | |  |
|  |  |  |  |  |  |  |  |  |  |  |  |  | Multivariate | | Intoxication (aOR=2·28, 95% CI 1·68-3·09, p<0·05) remained associated, while controlling for age, gender, and type of school. | | Positive | |  |  |  |
|  |  | |  |  | |  | |  | |  | |  | |  | |  | |  | |  | |
| **Alcohol** | Intoxication | | Muula et al, 2007 [40] | Zambia  School based | | Adolescents (14 to 16 years old) | | Cross-sectional study | | Males 919 (54.0%) & female 964 (46%) | | Suicidal ideation | | Bivariate | | Ever having been drunk (OR=1·40, 95% CI 1·39-1·41, p<0·05) was significantly associated with suicidal ideation. | | Positive | | ++ | |
|  |  |  |  |  |  |  |  |  |  |  |  |  |  | Multivariate | | This association remained significant (aOR=1·28, 95% CI 1·27–1·29, p<0·05), while controlling for age, sex, worry, loneliness, ever smoked marijuana, felt sad or hopeless. | | Positive | |  |  |
|  |  | |  |  | |  | |  | |  | |  | |  | |  | |  | |  | |
| **Alcohol** | Intoxication | | Myint et al, 2014 [41] | Thailand  Hospital based | | Both (10 to 79 years old) | | Cohort study | | Male 136 (91.3%) & 13 (8.7%) | | Fatal suicide | | Bivariate | | Blood alcohol concentrations (6 vs. 32 participants) (x^2^=0·527, df=1, p=0·468) were not significantly different when the suicide and non-suicide groups were compared. | | Null | | ++ | |
|  |  | |  |  | |  | |  | |  | |  | |  | |  | |  | |  | |
|  |  | |  |  | |  | |  | |  | |  | |  | |  | |  | |  | |
|  |  | |  |  | |  | |  | |  | |  | |  | |  | |  | |  | |
|  |  | |  |  | |  | |  | |  | |  | |  | |  | |  | |  | |
|  |  | |  |  | |  | |  | |  | |  | |  | |  | |  | |  | |
|  |  | |  |  | |  | |  | |  | |  | |  | |  | |  | |  | |
|  |  | |  |  | |  | |  | |  | |  | |  | |  | |  | |  | |
|  |  | |  |  | |  | |  | |  | |  | |  | |  | |  | |  | |
|  |  | |  |  | |  | |  | |  | |  | |  | |  | |  | |  | |
| **Alcohol** | Intoxication | | Page et al, 2011 [36] | China; Philippines  School based | | Adolescent 11 to 17 years old) | | Cross-sectional study | | China: male 4356 (51.2%) & female 4537 (48.8%).  Philippines: male 3094 (43.2%) & female 4188 (56.8%). | | Suicidal ideation | | Multivariate | | Among Philippine students, ever been drunk was significantly associated with suicide ideation (aOR=1·34, 95% CI 1·31-1·36, p<0·001) and making a suicide plan (aOR=1·29, 95% CI 1·27-1·31, p<0.001), while controlling for age, grade level, and greater distribution in the two samples. | | Positive | | ++ | |
|  |  |  |  |  |  |  |  |  |  |  |  |  |  |  | | Among Chinese students, ever been drunk was significantly associated with suicide ideation (aOR=28·2, 95% CI 24·7-31·8, p<0·001) and making a suicide plan (aOR=1·09, 95% CI 1·05-1·13, p<0·001), while controlling for age, grade level, and greater distribution in the two samples. | | Positive | |  |  |
|  |  | |  |  | |  | |  | |  | |  | |  | |  | |  | |  | |
|  |  | |  |  | |  | |  | |  | |  | |  | |  | |  | |  | |
|  |  | |  |  | |  | |  | |  | |  | |  | |  | |  | |  | |
| **Alcohol** | Intoxication | | Sitdhiraksa et al, 2014 [33] | Thailand  School based | | Both (12 to 21 years old) | | Cross-sectional study | | Male 1054 (40.2%) & female 1568 (59.8%) | | Suicidal ideation: Thoughts of self-harm  Non-fatal suicidal behaviour: Suicide attempt | | Bivariate | | Being drunk within the last 12 months was significantly associated with thoughts of harming yourself or attempted suicide (OR=3·36, 95% CI 2·74-4·12, p<0·01). | | Positive | | + | |
|  |  | |  |  | |  | |  | |  | |  | |  | |  | |  | |  | |
| **Alcohol** | Intoxication | | de Mattos Souza et al, 2010 [38] | Brazil  National/regional registers (e.g. not clinical-based registers) | | Adolescents (11 to 15 years old) | | Cross-sectional study | | Male 501 (48.2%) &  female 538 (51.8%) | | Suicidal ideation | | Multivariate | | Getting drunk in the last month was not associated with suicidal ideation (aOR=1·94, 95% CI 0·86-4·36, p=0·109), while controlling for gender, age, socioeconomic status, level of education, grade retention, religious practice, sexual activity, alcohol consumption, illicit drug use, conduct disorder and high CDI scores for depressive symptoms. | | Null | | ++ | |
|  |  | |  |  | |  | |  | |  | |  | |  | |  | |  | |  | |
| **Alcohol** | Intoxication | | Swahn et al, 2012 [32] | Uganda  Other | | Both (14 to 24 years old) | | Cross-sectional study | | Male 142 (31.1%) & female 315 (68.5%) | | Suicidal ideation | | Bivariate | | Any drunkenness (OR=1·95, 95% CI 1·29-2·97, p<0·05), significantly associated with suicide ideation. | | Positive | | ++ | |
|  |  |  |  |  |  |  |  |  |  |  |  | Suicidal ideation | | Multivariate | | The association between any drunkenness and suicidal ideation disappeared (aOR=0·93, 95% CI 0·49-1·75, p<0·05), while controlling for: gender, school attendance, whether one or both parents were dead, parental physical abuse, parental neglect due to alcohol use, apprenticeship skills, any drug use, any STD/HIV, any traded sex, any rape, sadness, lonely, expect to die early. | | Null | |  |  |
|  |  |  |  |  |  |  |  |  |  |  |  | Non-fatal suicidal behaviour: suicide attempt | | Bivariate | | Any drunkenness (OR=1·97, 95% CI 1·22-3·18, p<0·05), significantly associated with suicide attempt. | | Positive | |  |  |
|  |  |  |  |  |  |  |  |  |  |  |  | Non-fatal suicidal behaviour: suicide attempt | | Multivariate | | The association between any drunkenness and suicidal attempt disappeared while controlling for the other variables in the model (aOR=1·00, 95% CI 0·51-1·95, p<0·05), while controlling for gender, school attendance, whether one or both parents were dead, parental physical abuse, parental neglect due to alcohol use, apprenticeship skills, any drug use, any STD/HIV, any traded sex, any rape, sadness, lonely, expect to die early. | | Null | |  |  |
|  |  | |  |  | |  | |  | |  | |  | |  | |  | |  | |  | |
|  |  | |  |  | |  | |  | |  | |  | |  | |  | |  | |  | |
|  |  | |  |  | |  | |  | |  | |  | |  | |  | |  | |  | |
|  |  | |  |  | |  | |  | |  | |  | |  | |  | |  | |  | |
|  |  | |  |  | |  | |  | |  | |  | |  | |  | |  | |  | |
|  |  | |  |  | |  | |  | |  | |  | |  | |  | |  | |  | |
|  |  | |  |  | |  | |  | |  | |  | |  | |  | |  | |  | |
|  |  | |  |  | |  | |  | |  | |  | |  | |  | |  | |  | |
|  |  | |  |  | |  | |  | |  | |  | |  | |  | |  | |  | |
| **Alcohol** | Intoxication | | Wei et al, 2013 [34] | China  Hospital based | | Both (15 to 60 years old) | | Cohort study | | Male 53 (22.2%) & female 186 (77.8%) | | Non-fatal suicidal behaviour: Suicide attempt | | Bivariate | | A greater proportion of those who reported alcohol use at the time of the episode or up to 12 hours before reported impulsive suicide attempt when compared to nonimpulsive suicide attempt. This was not a statistically significant difference (14% vs. 9·8%), (x^2^=0·994, df=1, p=0·319). | | Null | | + | |
|  |  | |  |  | |  | |  | |  | |  | |  | |  | |  | |  | |
| **Alcohol** | Intoxication | | Zhu et al, 2015 [37] | China;  Vietnam;  Taiwan  Community based | | Adolescents (15 to 24 years old) | | Cross-sectional study | | N/A | | Suicidal ideation | | Multivariate | | Drunkness predicted suicidal ideation when controlling for city (Hanoi, Shanghai, Taipei) (aOR=2·12, 95% CI 1·33–3·38, p<0·01). | | Positive | | + | |
|  |  |  |  |  |  |  |  |  |  |  |  |  |  |  |  | Suicidal ideation was significantly associated with drunkness in Hanoi (aOR=2·12, 95% CI 1·33–3·38, p<0·01), Shanghai (aOR=2·18, 95% CI 1·47–3·23, p<0·001), Taipei (aOR=1·97, 95% CI 1·57–2·47, p<0·001). | | Positive | |  |  |
|  |  | |  |  | |  | |  | |  | |  | |  | |  | |  | |  | |
| **Alcohol** | Use | | Ahmad et al, 2014 [45] | Malaysia  School-based | | Adolescents (12 to 17 years old) | | Cross-sectional study | | Male (49.6%) & female (50.4 %) | | Suicidal ideation | | Multivariate | | Current drinking was associated with suicidal ideation (aOR=1·55, 95% CI 1·28-1·87, p<0·01), while controlling for gender, ethnicity, parental marital status, current smoking, current drug use, were bullied, physically abused at home, verbally abused at home, stress, anxiety, depression, have close friend, supportive peers, parental supervision, parental connectedness, parental bonding, parental respect for privacy) | | Positive | | ++ | |
|  |  | |  |  | |  | |  | |  | |  | |  | |  | |  | |  | |
| **Alcohol** | Use | | Alvarado-Esquivel et al, 2014 [43] | Mexico  Hospital-based | | Adults [sample: 18-61 years old (mean 34.14+/-  10.24 years old). Control:  18-69 years old (mean 38.23+/-11.76 years old)] | | Case-control study | | Male 85 (30.8%) & female 191 (69.2%) | | Non-fatal suicidal behaviour: Suicide attempt | | Multivariate | | Alcohol consumption (OR=2·39, 95% CI 1·21-4·70, p=0·01) was associated with suicide attempts. | | Positive | | + | |
|  |  | |  |  | |  | |  | |  | |  | |  | |  | |  | |  | |
| **Alcohol** | Use | | Arenliu et al, 2014 [60] | Kosovo  School-based | | Adolescents (15-19 years old) | | Cross-sectional study | | Male (43.7%) & female 55.1%; not responded 1.2% | | Suicidal ideation | | Bivariate | | Alcohol usage over 30 days was associated with suicidal ideation among both males (OR=2·36, 95% CI 1·43–3·82, p<0·001), and females  (OR=3·24, 95% CI 1·93–5·28, p<0·001). | | Positive | | - | |
|  |  |  |  |  |  |  |  |  |  |  |  | Non-fatal suicidal behaviour: Suicide attempt | | Bivariate | | Alcohol usage over 30 days was associated with suicide attempt for both males (OR=2·90, 95% CI 1·51–5·4, p<0·05), for females (OR=2·34, 95% CI 0·92–5·15, p<0·05). | | Positive | |  |  |
|  |  | |  |  | |  | |  | |  | |  | |  | |  | |  | |  | |
|  |  | |  |  | |  | |  | |  | |  | |  | |  | |  | |  | |
| **Alcohol** | Use | | Blum et al, 2012 [46] | Vietnam, and Taiwan  Community based (15 to 24 years old) | | Both (15 to 24 years old) | | Cross-sectional study | | Hanoi (Vietnam): male 3 251 (52.5%) & female 2 940 (47.5%)  Taipei (Taiwan): male 2398 (51%) & female 2308 (49%) | | Suicidal ideation | | Bivariate | | Alcohol use in the past month was associated with suicidal ideation in Hanoi (OR=1·82, 95% CI 1·21-2·74, p<0·01) and Taipei (OR=1·54, 95% CI 1·26-1·88, p<0·01) respectively. | | Positive | | ++ | |
|  |  |  |  |  |  |  |  |  |  |  |  | Non-fatal suicidal behaviour: Suicide attempt | | Bivariate | | Alcohol use in the last month was not associated with suicide attempts in Hanoi, (OR=1·1·58, 95% CI 39-6·41, p<0·05). | | Null | |  |  |
|  |  |  |  |  |  |  |  |  |  |  |  | Non-fatal suicidal behaviour: Suicide attempt | | Bivariate | | Alcohol use in the last month was associated with suicide attempts in Taipei (OR=1·50, 95% CI 1·04 -2·17, p<0·05). | | Positive | |  |  |
|  |  | |  |  | |  | |  | |  | |  | |  | |  | |  | |  | |
| **Alcohol** | Use | | Chan et al, 2013 [47] | Malaysia  National/regional registers (e.g. not clinical-based registers) | | Adolescent (18 to 76 years old) | | Cross-sectional study | | Male 2407 (47.5%) & female 2174 (52.5%) | | Non-fatal suicidal behaviour: deliberate self-harm | | Multivariate | | Alcohol use (aOR=1·34, 95% CI 1·00-1·79, p<0·048) was significantly associated with DSH, while controlling for history of sexual abuse, illicit drug use, and female gender. | | Positive | | - | |
|  |  | |  |  | |  | |  | |  | |  | |  | |  | |  | |  | |
| **Alcohol** | Use | | Chaveepojnkamjorn et al, 2011 [48] | Thailand  School-based | | Adolescents (15 to 18 years old) | | Cross-sectional study | | Male (100%) | | Suicidal ideation: thought and plan | | Multivariate | | Current drinkers, when compared to non-drinkers, were more likely to report serious thoughts about suicide (aOR=2·07, 95% CI 1·38-3·11) and making a suicide plan (aOR=2·10, 95% CI 1·43-3·08, p<0·001), while controlling for socio-demographic factors (age, educational level, residence, cohabitants, GPA, having a part time job, and having family members with alcohol/drug problems). | | Positive | | ++ | |
|  |  | |  |  | |  | |  | |  | |  | |  | |  | |  | |  | |
| **Alcohol** | Use | | Diehl & Laranjeira, 2009 [44] | Brazil  Hospital based | | Adult (18 to 41 years old) | | Cohort study | | Male 22 (27.5%) & female 58 (72.5 %) | | Non-fatal suicidal behaviour: Suicide attempt | | Bivariate | | Suicide attempt method was not associated with alcohol use within six hours prior to the event (p=0·346). No other statistical results were reported. | | Null | | - | |
|  |  | |  |  | |  | |  | |  | |  | |  | |  | |  | |  | |
| **Alcohol** | Use | | Jaisoorya et al, 2015 [63] | India  School based | | Adolescents (12 to 19 years old) | | Cross-sectional study | | Male 865 (23.2%) & female 235  (6.5%) | | Suicidal ideation: Suicidal thoughts  Non-fatal suicidal behaviour: Suicide attempts | | Multivariate | | Suicidal thoughts (aOR=1·7, 95% CI 1·4-2·1, p<0·05) and suicide attempts (aOR=0·9, 95% CI 0·7-1·4, p<0·05) predicted lifetime use of alcohol, while controlling for socio-demographic variables, academic performance, other substance use, self-reported psychological distress scores, suicidality, sexual abuse and ADHD scores. | | Positive | | ++ | |
|  |  | |  |  | |  | |  | |  | |  | |  | |  | |  | |  | |
| **Alcohol** | Use | | Khan, Sulaiman, & Hassali, 2012 [49] | Malaysia  Clinic-based (e.g. mobile clinic, free clinic) | | Both (15 to 84 years old) | | Cross-sectional study | | Male 128 (43%) & female 170 (57%) | | Suicidal ideation | | Bivariate | | Those depressive disorder patients disclosing alcohol use (x^2^=73·3, df=1, p< 0·01) were more likely to report suicidal ideation | | Positive | | - | |
|  |  |  |  |  |  |  |  |  |  |  |  |  |  |  |  | Those depressive disorder patients with alcohol use (OR=3·69, 95% CI 1·99-6·85, p<0·01) had the highest risk of suicidal ideation in comparison to others (i.e., those with a history of medical complications and social problems) | |  |  |  |  |
|  |  | |  |  | |  | |  | |  | |  | |  | |  | |  | |  | |
| **Alcohol** | Use | | Khasakhala et al, 2013 [65] | Kenya  Hospital based | | Both (13 to 25 years old) | | Cross-sectional study | | Male 447 (48.7%) & female 471 (51.3%) | | Non-fatal suicidal behaviour: Suicidal behaviour | | Multivariate | | Any drug abuse (OR=6·66, 95% CI 2·81–15·75, p<0·001), and alcohol use (OR=6·69, 95% CI 2·69–16·6, p<0·001) was associated with suicidal behaviour. | | Positive | | ++ | |
|  |  |  |  |  |  |  |  |  |  |  |  |  |  |  |  | Any drug use was associated with suicidal behaviour (aOR=5·23, 95% CI 1·88–13·9, p<0·001), while controlling for major depressive disorder, any anxiety disorder, and alcohol use. | | Positive | |  |  |
|  |  | |  |  | |  | |  | |  | |  | |  | |  | |  | |  | |
| **Alcohol** | Use | | Miller et al, 2011 [56] | Mexico  Community based | | Adolescents (12 to 17 years old) | | Cross-sectional study | | N/A | | Suicidal ideation | | Bivariate | | Lifetime use of any alcohol beverages (OR=2·13, 95% CI 1·63-2·78, p<0·01) and lifetime use of 12 or more drinks in a year (OR=2·06, 95% CI 1·56-2·73, p<0·01) was associated with suicidal ideation. | | Positive | | ++ | |
|  |  |  |  |  |  |  |  |  |  |  |  | Suicidal ideation | | Bivariate | | Lifetime use of any alcohol beverages (OR=3·87, 95% CI 2·15-6·98, p<0·01) and lifetime use of 12 or more drinks in a year (OR=2·25, 95% CI 1·35-3·74, p<0·01) was associated with suicide plan. | | Positive | |  |  |
|  |  |  |  |  |  |  |  |  |  |  |  | Non-fatal suicidal behaviour: Suicide attempt | | Bivariate | | Lifetime use of any alcohol beverages (OR=3·43, 95% CI 1·88-6·29, p<0·01) and lifetime use of 12 or more drinks in a year (OR=2·26, 95% CI 1·32-3·86, p<0·01) was associated with suicide attempt. | | Positive | |  |  |
|  |  |  |  |  |  |  |  |  |  |  |  | Suicidal ideation | | Multivariate | | Lifetime use of any alcohol beverages (OR=1·21, 95% CI 0·91-1·62, p<0·05) and lifetime use of 12 or more drinks in a year (OR=1·02, 95% CI 0·58-1·81, p>0·05) was not associated with suicidal ideation, while controlling for psychiatric disorder or drug use/disorder and tobacco use/disorder. | | Null | |  |  |
|  |  |  |  |  |  |  |  |  |  |  |  | Suicidal ideation | | Multivariate | | Lifetime use of any alcohol beverages (OR=1·85, 95% CI 1·18-2·90, p<0·01) was associated with suicide plan, while controlling for psychiatric disorder or drug use/disorder and tobacco use/disorder. | | Positive | |  |  |
|  |  |  |  |  |  |  |  |  |  |  |  | Suicidal ideation | | Multivariate | | Lifetime use of 12 or more drinks in a year (OR=0·79, 95% CI 0·30-2·12, p>0·05) was not associated with suicide plan, while controlling for psychiatric disorder or drug use/disorder and tobacco use/disorder. | | Null | |  |  |
|  |  |  |  |  |  |  |  |  |  |  |  | Non-fatal suicidal behaviour: Suicide attempt | | Multivariate | | Lifetime use of any alcohol beverages (OR=1·66, 95% CI 1·01-2·73, p>0·05) and lifetime use of 12 or more drinks in a year (OR=0·56, 95% CI 0·29-1·09, p>0·05) was not associated with suicide attempt, while controlling for psychiatric disorder or drug use/disorder and tobacco use/disorder. | | Null | |  |  |
|  |  | |  |  | |  | |  | |  | |  | |  | |  | |  | |  | |
| **Alcohol** | Use | | Nojomi et al, 2007 [70] | Iran  Community based | | Both 14 years old and older | | Cross-sectional study | | Male 809 (35.2%) & Female 1491 (64.8 %) | | Non-fatal suicidal behaviour: Suicide attempt | | Bivariate | | Alcoholic beverages were higher among suicide attempters than nonattempters (31·6% vs. 8·8%, p<0·0005). | | Positive | | ++ | |
|  |  |  |  |  |  |  |  |  |  |  |  |  |  |  |  | Alcohol use was associated with lifelong suicide attempts (OR=3·80, 95% CI 1·82-7·95, p<0·001). | | Positive | |  |  |
|  |  | |  |  | |  | |  | |  | |  | |  | |  | |  | |  | |
| **Alcohol** | Use | | Page et al, 2011 [55] | Philippines;  China;  Namibia  School based | | Adolescents (11 to 16 years old) | | Cross-sectional study | | Philippines: male 3094 (43.2%) & female 4188 (56.8%).  China: male 4356 (51.2%) & female 4537 (48.8%).  Namibia: male 2931 (45.2%) & female 3352 (54.8%). | | Suicidal ideation | | Bivariate | | Current drinking was associated with suicide plan across gender and the three countries (Philippine, China, Namibia):  Philippine boys %(n) drinker=22·7 (210); %(n) nondrinker=16·8 (269); (OR=1·39, 95% CI 1·12-1·72, p<0·05);  Philippine girls %(n) drinker=26·3 (188); %(n) nondrinker= 15·9 (477); (OR=1·93, 95% CI 1·58-2·36, p<0·05);  China boys %(n) drinker=11·8 (84); %(n) nondrinker= 5·2 (166); (OR=2·50, 95% CI 1·88-3·30, p<0·05);  China girls %(n) drinker=21·5 (92); %(n) nondrinker= 7·9 (300); (OR=3·12, 95% CI 2·40-4·06, p<0·05);  Namibia boys %(n) drinker=36·6 (301); %(n) nondrinker= 27·0 (438); (OR= 1·52, 95% CI 1·26-1·83, p<0·05);  Namibia girls %(n) drinker=37·9 (323); %(n) nondrinker= 27·3 (558); (OR=1·64, 95% CI 1·37-1·95, p<0·05). | | Positive | | ++ | |
|  |  | |  |  | |  | |  | |  | |  | |  | |  | |  | |  | |
| **Alcohol** | Use | | Page, West, et al, 2011 [36] | China; Philippines  School based | | Adolescent 11 to 17 years old) | | Cross-sectional study | | China: male 4356 (51.2%) & female 4537 (48.8%).  Philippines: male 3094 (43.2%) & female 4188 (56.8%). | | Suicidal ideation | | Multivariate | | Use of alcohol in the past 30 days was associated with suicide ideation (OR=1·67, 95% CI 1·63-17·1, p<0·001) and making a suicide plan (OR=2·46, 95% CI 2·39-2·53, p<0·001) in Chinese students, while controlling for age, grade level, and greater distribution in the 2 samples. | | Positive | | ++ | |
|  |  |  |  |  |  |  |  |  |  |  |  |  |  |  |  | Use of alcohol in the past 30 days was associated with suicide ideation (OR=1·10, 95% CI 1·08-1·12, p<0·001) and making a suicide plan (OR=1·03, 95% CI 1·02-1·05, p<0·001) , while controlling for age, grade level, and greater distribution in the 2 samples. | | Positive | |  |  |
|  |  | |  |  | |  | |  | |  | |  | |  | |  | |  | |  | |
| Alcohol | Use | | Peltzer, 2008 [66] | South Africa  School based | | Adolescents (15 to 18 years old) | | Cross-sectional study | | Male | | Non-fatal suicidal behaviour: suicide risk related behaviours | | Multivariate | | Past month frequency of beer or wine (OR=1·23, 95% CI 0·85-1·84, p>0·05) and hard liquor (OR=1·36, 95% CI 0·92-2·09, p>0·05) not significantly associate with high vs. low suicide risk. | | Null | | ++ | |
|  |  | |  |  | |  | |  | |  | |  | |  | |  | |  | |  | |
| **Alcohol** | Use | | Peltzer, 2009 [67] | Kenya; Namibia; Swaziland;  Uganda; Zambia; Zimbabwe  School based | | Adolescents (13 to 15 years old) | | Cross-sectional study | | N/A | | Suicidal ideation | | Bivariate | | Risky drinking was associated with suicidal ideation (OR=2·37, 95% CI 2·34–2·41, p<0·001). | | Positive | | + | |
|  |  |  |  |  |  |  |  |  |  |  |  | Suicidal ideation | | Multivariate | | Suicidal ideation was associated with risky drinking (aOR=1·66, 95% CI 1·63–1·69, p<0·001), while controlling for confounding factors. | | Positive | |  |  |
|  |  |  |  |  |  |  |  |  |  |  |  | Suicidal ideation | | Bivariate | | Risky drinking was associated with suicide plan (OR=2·52, 95% CI 2·48–2·55, p<0·001). | | Positive | |  |  |
|  |  |  |  |  |  |  |  |  |  |  |  | Suicidal ideation | | Multivariate | | Risky drinking was associated with suicide plan (aOR=1·59, 95% CI 1·56–1·62, p<0.001), while controlling for confounding factors. | | Positive | |  |  |
|  |  | |  |  | |  | |  | |  | |  | |  | |  | |  | |  | |
| **Alcohol** | Use | | Peltzer & Pengpid, 2015 [50] | Oceania (Kiribati,  Samoa, Solomon Islands, and Vanuatu)  School based | | Adolescents (13 to 16 years old) | | Cross-sectional study | | Male 2846 (43.5%) & female 3534 (54%) | | Suicidal ideation  Non-fatal suicidal behaviour: Suicide attempt | | Bivariate | | Alcohol use initiation  was associated with suicidal ideation:  **Among the total sample**  Non-initiators 1.00  <12 years (OR=4·14, 95% CI 3·52-4·86, p<0·001);  ≥12 years (OR=2·48, 95% CI 2·01-3·06, p<0·001).  **Among boys**  Non-initiators 1.00  <12 years (OR=3·55, 95% CI 2·6–4·74, p<0·001);  ≥12 years (OR=2·12, 95% CI 1·5–2·92, p<0·001).  AND  **Among girls**  Non-initiators 1·00  <12 years (OR=4·89, 95% CI 3·63–6·59, p<0·001);  ≥12 years (OR=3·14, 95% CI 2·41–4·09, p<0·01). | | Positive | | ++ | |
|  |  |  |  |  |  |  |  |  |  |  |  |  |  | Bivariate | | Current alcohol use was associated with suicidal ideation (OR=2·69, 95% CI 2·3-3·12, p<0·001).  **Among boys**  (OR=2·37, 95% CI 1·9-2·87, p<0·001);  **Among girls**  (OR=3·20, 95% CI 2·54-4·03, p<0·001). | | Positive | |  |  |
|  |  |  |  |  |  |  |  |  |  |  |  |  |  | Bivariate | | Alcohol use initiation  was associated with suicide attempt:  **Among the total sample**  Non-initiators 1·00  <12 years (OR = 8·48, 95% CI 6·51-11·1, p<0·001);  ≥12 years (OR=2·89, 95% CI 2·26-3·70, p<0·001).  **Among boys**  Non-initiators 1.00  <12 years (OR=7·63, 95% CI 5·18–11·2, p<0·001);  ≥12 years (OR=2·37, 95% CI 1·75–3·20, p<0·001).  AND  **Among girls**  Non-initiators 1·00  <12 years (OR=4·89, 95% CI 3·63–6·59, p<0·001);  ≥12 years OR=3·14, 95% CI 2·41–4·09, p<0·001). | | Positive | |  |  |
|  |  |  |  |  |  |  |  |  |  |  |  |  |  | Bivariate | | Current alcohol use was associated with suicide attempt (OR=2·69, 95% CI 2·3-3·12, p<0·001).  **Among boys**  (OR=2·37, 95% CI 1·9-2·87, p<0·001);  **Among girls**  (OR=3·20, 95% CI 2·54-4·03, p<0·001). | | Positive | |  |  |
|  |  |  |  |  |  |  |  |  |  |  |  |  |  | Multivariate | | Alcohol use initiation  was associated with suicidal ideation:  **Among the total sample**  Non-initiators 1·00  <12 years (aOR=3·39, 95% CI 2·44-4·71, p<0·001);  ≥12 years (aOR=1·95, 95% CI 1·32-2·89, p<0·001).  **Among boys**  Non-initiators 1.00  <12 years (aOR=3·37, 95% CI 2·16-5·27, p<0·001);  ≥12 years (aOR=1·88, 95% CI 1·14-3·10, p<0·05).  AND  **Among girls**  Non-initiators 1·00  <12 years (aOR=3·12, 95% CI 1·95-4·90, p<0·001);  ≥12 years (aOR=2·12, 95% CI 1·34-3·34, p<0·01).  (Adjusted for age, psychological distress and current  alcohol use) | | Positive | |  |  |
|  |  |  |  |  |  |  |  |  |  |  |  |  |  | Multivariate | | Alcohol use initiation  was associated with suicide attempt:  **Among the total sample**  Non-initiators 1·00  <12 years (aOR=4·55, 95% CI 3·34-6·21, p<0·001);  ≥12 years (aOR=1·64, 95% CI 1·16-2·32, p<0·01).  **Among boys**  Non-initiators 1.00  <12 years (aOR=3·94, 95% CI 3·46-6·32, p<0·001); but not  ≥12 years (aOR=1·19, 95% CI 0·77-1·85, p>0·05).  AND  **Among girls**  Non-initiators 1·00  <12 years (aOR=5·76, 95% CI 3·84-8·64, p<0·001);  ≥12 years (aOR=1·84, 95% CI 1·05-3·22, p<0·05).  (Adjusted for age, psychological distress and current  alcohol use) | | Positive | |  |  |
| **Alcohol** | Use | | Pillai et al, 2009 [64] | India  Community based | | Adolescents (16 to 24 years old) | | Cross-sectional study | | Male 1780 (49.6%) & females 1882 (51.4%) | | Non-fatal suicidal behaviour: Suicide behaviour | | Multivariate | | Alcohol use (aOR=2·7, 95% CI 1·7-4·4, p<0·05) and was associated with suicidal thinking/planning/attempts during past 3 months. | | Positive | | ++ | |
|  |  | |  |  | |  | |  | |  | |  | |  | |  | |  | |  | |
| **Alcohol** | Use | | Pumariega et al, 2014 [61] | Turkey  School based | | Adolescents (14 to 18 years old) | | Cross-sectional study | | Male 14 477 (46.6%) & female 16581 (53.4%) | | Suicidal ideation | | Bivariate | | Suicidal ideation was a was associated with alcohol use (OR=0·878, NO CI, p=0·043). | | Positive | | + | |
|  |  | |  |  | |  | |  | |  | |  | |  | |  | |  | |  | |
| **Alcohol** | Use | | Reyes-Tovilla et al, 2015 [57] | Mexico  Hospital based | | Both (13 to 60 years old) | | Cohort study | | Male 52 (36.1%) &  female 92 (63.9%) | | Non-fatal suicidal behaviour: Suicide attempt | | Multivariate | | Alcohol consumption was associated with suicide attempt (OR=7·48, 95% CI 1·64-34·2, p=0·009), while controlling for past number of attempts, intake of alcohol prior to attempt, cannabis use, intake of cannabis prior to attempt. | | Positive | | + | |
|  |  |  |  |  |  |  |  |  |  |  |  |  |  |  |  | Intake of alcohol prior to attempt was not associated with whether an attempt was impulsive or premeditated (OR=2·78, 95% CI 0·52-14·9, p=0·23), while controlling for past number of attempts, alcohol consumption, cannabis use, intake of cannabis prior to attempt. | | Null | |  |  |
|  |  | |  |  | |  | |  | |  | |  | |  | |  | |  | |  | |
| **Alcohol** | Use | | Rudatsikira et al, 2007 [68] | Uganda  School based | | Adolescents (11 to 17 years old) | | Cross-sectional study | | Male 784 (53.3 %) & female 676 (46.7%) | | Suicidal ideation | | Bivariate | | Alcohol Drinking in the past 12 months was not associated with suicidal ideation among the total sample (OR=1·40, 95% CI 0·99-2·00, p>0·05). | | Null | | - | |
|  |  |  |  |  |  |  |  |  |  |  |  |  |  | Multivariate | | Alcohol drinking in the last 12 months did not significantly predict suicidal ideation (OR=1·27, 95% CI 0·80-2·01, p>0·05), while controlling for age, gender, loneliness, worry, smoking and being bullied. | | Null | |  |  |
|  |  | |  |  | |  | |  | |  | |  | |  | |  | |  | |  | |
| **Alcohol** | Use | | Rudatsikira et al, 2007 [69] | Zimbabwe  School based | | Adolescents (11 to 17 years old) | | Cross-sectional study | | Male 873 (49.3%) & female 1111 (50.7%) | | Suicidal ideation | | Bivariate | | Drinking alcohol in the past 12 months was associated with suicidal ideation among the total sample (OR=2·24, 95% CI 1·57- 3·19, p<0·05), men (OR=2·28 95% CI 1·34-3·86, p<0·05) and women (OR=2·21, 95% CI 1·36-3·60, p<0·05). | | Positive | | - | |
|  |  | |  |  | |  | |  | |  | |  | |  | |  | |  | |  | |
| **Alcohol** | Use | | Ruengorn et al, 2012 [51] | Thailand  Hospital based | | Both (13 to 60 years old) | | Case-control study | | Male 354 (32.2%) & female 746 (67.8%) | | Non-fatal suicidal behaviour: Suicide attempt | | Bivariate | | Current alcohol use was associated with suicide attempt (OR=2·78, 95% CI 1·91-4·03, p<0·001). | | Positive | | - | |
|  |  | |  |  | |  | |  | |  | |  | |  | |  | |  | |  | |
| **Alcohol** | Use | | Sharma et al, 2015 [58] | Peru  School based | | Adolescents (12 to 18 years old) | | Cross-sectional study | | Male 425 (46.4%) & female 491 (53.6%) | | Non-fatal suicidal behaviour: Suicide attempt | | Multivariate | | Alcohol consumption was associated with increased likelihood of suicide attempts (aOR=1·59, 95% CI 1·04-2·44, p<0·05), while controlling for psychological, and behavioural factors. | | Positive | | ++ | |
|  |  |  |  |  |  |  |  |  |  |  |  |  |  |  |  | Alcohol consumption was significantly associated with increased likelihood of suicide attempts (aOR=1·52, 95% CI 1·00–2·33, p<0·05), while controlling for psychological, socio-environmental, and demographic factors. | | Positive | |  |  |
|  |  | |  |  | |  | |  | |  | |  | |  | |  | |  | |  | |
| **Alcohol** | Use | | Shooshtary et al, 2008 [71] | Iran  Community based | | Both (15 years old and older) | | Cross-sectional study | | Male 187 (37.1%) & female 317 (62.9%) | | Non-fatal suicidal behaviour: Suicide attempt | | Bivariate | | Alcohol use was not associated with lifetime suicide attempts (OR=0·67, 95% CI 0·15-2·91, p>0·05). | | Null | | ++ | |
|  |  | |  |  | |  | |  | |  | |  | |  | |  | |  | |  | |
| **Alcohol** | Use | | Silva et al, 2014 [59] | Brazil  School based | | Adolescents (13 to 18 years old) | | Cross-sectional study | | Male 836 (37.9%) & female 1371 (62.1%) | | Suicidal ideation  Non-fatal suicidal behaviour:  Suicide attempt | | Bivariate | | Alcohol consumption, association was found with suicide ideation (OR=1·93, CI 95% 1·47-2·54, p<0·01), planning (OR=2·22, CI 95% 1·61–3·08, p<0·01), and attempt (OR=1·73, CI 95% 1·15–2·59, p<0·01). | | Positive | | + | |
|  |  | |  |  | |  | |  | |  | |  | |  | |  | |  | |  | |
| **Alcohol** | Use | | Sitdhiraksa et al, 2014 [33] | Thailand  School based | | Both (12 to 21 years old) | | Cross-sectional study | | Male 1054 (40.2%) & female 1568 (59.8%) | | Suicidal ideation: Thoughts of self-harm  Non-fatal suicidal behaviour: Suicide attempt | | Bivariate | | Alcohol use within the last 12 months was associated with thoughts of harming yourself or attempted suicide (OR=2·90, 95% CI 2·36-3·56, p<0·01). | | Positive | | + | |
|  |  | |  |  | |  | |  | |  | |  | |  | |  | |  | |  | |
| **Alcohol** | Use | | Souza et al, 2010 [38] | Brazil  National/ regional registers (e.g. not clinical-based registers) | | Adolescents (11 to 15 years old) | | Cross-sectional study | | Male 501 (48.2%) &  female 538 (51.8%) | | Suicidal ideation | | Multivariate | | Alcohol use in the last month was associated with suicidal ideation (aOR=1·64, 95% CI 1·04-2·58, p=0·033), while controlling for gender, age, socioeconomic status, level of education, grade retention, religious practice, sexual activity, tobacco use, getting drunk, conduct disorder and high CDI scores for depressive symptoms. | | Positive | | ++ | |
|  |  | |  |  | |  | |  | |  | |  | |  | |  | |  | |  | |
| **Alcohol** | Use | | Tran Thi Thanh et al, 2006 [52] | Vietnam  Community based | | Both (14 to 65 years old) | | Cross-sectional study | | Male 1093 (48.4%) & female 1167 (51.6%) | | Suicidal ideation: Suicidal thoughts | | Multivariate | | Having ever used alcohol was associated with suicidal thoughts (aOR=1·6, 95% CI 1·1-2·2, p<0·01), while controlling for age, gender, marital status, education level, income, religion, religiousness, employment status, use of alcohol, sedatives, and pain relief medication. | | Positive | | ++ | |
|  |  | |  |  | |  | |  | |  | |  | |  | |  | |  | |  | |
| **Alcohol** | Use | | Togay et al, 2015 [62] | Turkey  Hospital based | | Both (15 to 45 years old) | | Cross-sectional study | | Suicide attempt vs. no suicide attempt before admission: Male [12 (41.4%) vs. 91 (63.6%)] & female [17 (58.6%) vs. 52 (36.4%)].  Suicide attempt vs. no suicide attempt after admission: Male [8 (72.7%) vs. 59 (67.4%)] & female [3 (27.3%) vs. 28 (32.6%)]. | | Non-fatal suicidal behaviour: Suicide attempt | | Bivariate | | Alcohol use was associated with suicide attempts before first admission (OR=0·040, NO CI, p=0·045). | | Positive | | - | |
|  |  | |  |  | |  | |  | |  | |  | |  | |  | |  | |  | |
| **Alcohol** | Use | | Wan et al, 2011 [53] | China  School based | | Both (12 to 24 years old) | | Cross-sectional study | | Male 8599 (48.8%) & female 9023 (51.2%) | | Non-fatal suicidal behaviour: Deliberate self-harm | | Bivariate | | Among those with alcohol use, a total of 77% reported no DSH, 6% reported a single-incident DSH, 17% reported repeat-incident DSH. There was a significant difference between those with and those without alcohol use among DSH (x^2^=36·6, df=1, p<0·001). | | Positive | | ++ | |
|  |  |  |  |  |  |  |  |  |  |  |  |  |  |  |  | Alcohol use significantly predicted a single-incident DSH (OR=1·79, 95% CI 1·46–2·20, p<0·001), and repeat-incident DSH when controlling for single-incident DSH (OR=0·79, 95% CI 0·63–0·99, p=0·041). | | Positive | |  |  |
|  |  | |  |  | |  | |  | |  | |  | |  | |  | |  | |  | |
| **Alcohol** | Use | | Xiao et al, 2013 [42] | China  Community based | | Both (15 to 65 years old) | | Cross-sectional study | | Male 144 (33.8%) & female 282 (66.2%) | | Non-fatal suicidal behaviour: Self-harm | | Bivariate | | A greater proportion of light self-harm patients reported preceding alcohol intake when compared to severe self-harm (77·9% vs. 22·1%) (x^2^=7·4, df=1, p<0·05). | | Positive | | ++ | |
|  |  |  |  |  |  |  |  |  |  |  |  |  |  | Multivariate | | Preceding alcohol drinking was inversely related to severe self-harm, (OR=-0·40, 95% CI 0·22-0·74, p<0·01), while adjusting for the other factors in the model: gender, age, place of residence, and method of self-harm. | | Negative | |  |  |
|  |  | |  |  | |  | |  | |  | |  | |  | |  | |  | |  | |
| **Alcohol** | Use | | Xing et al, 2010 [54] | China  School based | | Adolescents (11 to 19 years old) | | Cross-sectional study | | Males 6216 (49.8%) & female 6254 (50.2%) | | Non-fatal suicidal behaviour: Suicide attempts | | Bivariate | | Being an alcohol user was associated with being a suicide attempter (x^2^=83, df=1, p<0·001). | | Positive | | ++ | |
|  |  | |  |  | |  | |  | |  | |  | |  | |  | |  | |  | |
| **Alcohol** | Misuse | | Fekadu et al, 2014 [147] | Ethiopia  Community based | | Adults (18 years old and older | | Cross-sectional study | | Male 743 (49.6%) & female 754 (50.4%) | | Non-fatal suicidal behaviour: suicidality | | Bivariate | | Hazardous alcohol use was not associated with suicidality (OR=1·19, 95% CI 0·85-1·65, p=0·305). | | Null | | ++ | |
|  |  | |  |  | |  | |  | |  | |  | |  | |  | |  | |  | |
| **Alcohol** | Misuse | | Peltzer & Louw, 2013 [86] | South Africa  Clinic-based (e.g., mobile clinic, free clinic) | | Adults (18 years old and older) | | Cross-sectional study | | Male 2631 (54.5%) & female 2194 (45.5%) | | Suicidal ideation  Non-fatal suicidal behaviour: Suicide attempt | | Multivariate | | Harmful alcohol use was associated with suicide ideation (aOR=1·97, 95% CI 12·5-3·09, p<0.01) and suicide attempts (aOR=3·01, 95% CI 1·83-4·95, p<0·001), while controlling for age, education, marital status, geolocality and poverty index. | | Positive | | ++ | |
|  |  | |  |  | |  | |  | |  | |  | |  | |  | |  | |  | |
| **Alcohol** | Misuse | | Randall et al, 2014 [83] | Republic of Benin, West Africa  School based | | Adolescents (11 to 16 years old) | | Cross-sectional study | | Male 1798 (67.1%) & female 882 (32.9%) | | Suicidal ideation  Non-fatal suicidal behaviour: Suicide attempt | | Bivariate | | Among alcohol misuse individuals:  15.3% (314) reported no suicidal ideation; 10·1% (13) reported ideation only; 26·7% (116) reported ideation with a plan, p<0·0001. | | Positive | | ++ | |
|  |  |  |  |  |  |  |  |  |  |  |  |  |  | Bivariate | | Alcohol misuse was significantly associated with number of suicide attempts p=0·002; no attempt=14·4% (281), one attempt =18·7%(65), 2 or more attempts =28·6% (95). | | Positive | |  |  |
|  |  |  |  |  |  |  |  |  |  |  |  |  |  | Multivariate | | Alcohol misuse was not associated with suicidal ideation (aOR=0·57, 95% CI 0·31–1·05, p=0·069), while controlling for age, psycho-social symptoms, and socio-environmental factors. | | Null | |  |  |
|  |  |  |  |  |  |  |  |  |  |  |  |  |  | Multivariate | | Alcohol misuse was associated with suicidal ideation with a plan (aOR=1·52, 95% CI 1·02–2·27, p=0·043), while controlling for age, psycho-social symptoms, and socio-environmental factors. | | Positive | |  |  |
|  |  |  |  |  |  |  |  |  |  |  |  |  |  | Multivariate | | Alcohol misuse, was not associated with having one past suicide attempt (aOR=1·11, 95% CI 0·65–1·90, p=0·672), while controlling for age, psycho-social symptoms, and socio-environmental factors. | | Null | |  |  |
|  |  |  |  |  |  |  |  |  |  |  |  |  |  | Multivariate | | Alcohol misuse, was not associated with having two or more past suicide attempt (aOR=1·62, 95% CI 0·97–2·70, p=0·063), while controlling for age, psycho-social symptoms, and socio-environmental factors. | | Null | |  |  |
|  |  | |  |  | |  | |  | |  | |  | |  | |  | |  | |  | |
| **Alcohol** | Misuse | | Razvodovsky, 2009 [140] | Belarus  Hospital based | | N/A | | Cohort study | | N/A | | Fatal suicide | | Bivariate | | The specification of the bivariate ARIMA model and outcome of the analysis are presented. The estimated effects of fatal alcohol poisoning rate (as a proxy for binge drinking) on total suicides number and number of BAC-positive suicides are statistically significant:  Suicide total: (Model=0,1,1*; Estimates=0·088, p<0·000);  Suicide BAC+: (Model=0,1,1*; Estimates=0·111, p<0·000). | | Positive | | + | |
|  |  | |  |  | |  | |  | |  | |  | |  | |  | |  | |  | |
| **Alcohol** | Misuse | | Razvodovsky, 2007 [75] | Belarus  Hospital based | | N/A | | Interrupted time-series | | N/A | | Fatal suicide | | Bivariate | | The results of time series analysis suggests close relationship between suicide and fatal alcohol poisoning rate at aggregate level. | | Positive | | - | |
|  |  | |  |  | |  | |  | |  | |  | |  | |  | |  | |  | |
| **Alcohol** | Misuse | | Razvodovsky, 2009 [76] | Russia  Hospital based | | N/A | | Interrupted time-series | | N/A | | Fatal suicide | | Bivariate | | Binge drinking was correlated with suicide rate for males (r=0·88; p<0·000) and a less strong one for females  (r=0·67; p<0·000). | | Positive | | - | |
|  |  | |  |  | |  | |  | |  | |  | |  | |  | |  | |  | |
| Alcohol | Abuse | | Peltzer, 2008 [66] | South Africa  School based | | Adolescents (15 to 18 years old) | | Cross-sectional study | | Male | | Non-fatal suicidal behaviour: suicide risk related behaviours | | Multivariate | | Level of problem alcohol use control:  Did not stop at one drink (0 to 6), predicted higher suicide risk (OR=1·17, 95% CI 1·00-1·35, p<0·05). | | Positive | | ++ | |
|  |  |  |  |  |  |  |  |  |  |  |  |  |  | Multivariate | | Level of problem alcohol use control:  Kept drinking/using even though had plenty already, predicted higher suicide risk (OR=1·23, 95% CI 1·07-1·40, p<0·01). | | Positive | |  |  |
|  |  | |  |  | |  | |  | |  | |  | |  | |  | |  | |  | |
| **Alcohol** | Abuse | | Altamura et al, 2007 [84] | Hospital-based | | Adults [South African Attempters = 31.2 (9.0); South African non-attempters = 39.3 (17.7)] | | Cross-sectional study | | Attempters: male 20 (64.5%) & female 11 (35.5%)  Non-attempters: male 3 (50%) & female 3 (50%) | | Non-fatal suicidal behaviour: Suicide attempt | | Bivariate | | Lifetime comorbid alcohol abuse was not associated with a suicide attempt during the course of their illness among schizophrenia spectrum disorder patients at high risk for suicide (x^2^=1·32, df=1, p>0·2). | | Null | | ++ | |
|  |  | |  |  | |  | |  | |  | |  | |  | |  | |  | |  | |
| **Alcohol** | Abuse | | Barbosa et al, 2014 [93] | Brazil  Population-based | | Both (14 to 35 years old) | | Cross-sectional study | | Male 594 (43.0 %) & female 786 (57.0 %) | | Non-fatal suicidal behaviour: Suicide risk (ideation, behaviour) | | Multivariate | | Alcohol abuse was associated with suicide risk (aOR=2·5, 95% CI 1·4-4·2, p<0·001), while controlling for gender, age, ethnicity, socioeconomic class, working, marital status, emotional neglect, physical neglect, sexual abuse, physical abuse, emotional abuse. | | Positive | | ++ | |
|  |  | |  |  | |  | |  | |  | |  | |  | |  | |  | |  | |
| **Alcohol** | Abuse | | Hooman et al, 2013 [95] | Iran  Hospital based | | Adults (Mean = 45.31; SD=13.7) | | Cross-sectional study | | Male 594 (43.0 %) & female 786 (57.0 %) | | Non-fatal suicidal behaviour: Suicide attempt | | Bivariate | | Alcohol abuse was associated with suicide attempt across both genders: males (OR=21·8, 95% CI 2·37-200·3, p=0·006) and females (OR=3·91, 95% CI 2·61-5·86, p<0·001). | | Positive | | ++ | |
|  |  | |  |  | |  | |  | |  | |  | |  | |  | |  | |  | |
| **Alcohol** | Abuse | | Menezes et al, 2012 [92] | Nepal  University students | | Adults (18 to 27 years old) | | Cross-sectional study | | Males 112 (54.4%) & females 94 (45.6%) | | Suicidal ideation | | Bivariate | | A greater proportion of suicidal ideation students reported consuming alcohol compared to those who did not consume alcohol (14·3% vs. 9·3%) but this was not a statistically significant difference (OR=1·62, 95% CI 0·64-4·09, p=0·309). | | Null | | ++ | |
|  |  | |  |  | |  | |  | |  | |  | |  | |  | |  | |  | |
| **Alcohol** | Abuse | | Miller et al, 2011 [56] | Mexico  Community based | | Adolescents (12 to 17 years old) | | Cross-sectional study | | N/A | | Suicidal ideation | | Bivariate | | Alcohol abuse or dependence was associated with suicidal ideation (OR=3·10, 95% CI 1·70-5·63, p<0·01). | | Positive | | ++ | |
|  |  |  |  |  |  |  |  |  |  |  |  | Suicidal ideation | | Bivariate | | Alcohol abuse or dependence was associated with a suicide plan (OR=4·21, 95% CI 1·83-9·6, p<0·01). | | Positive | |  |  |
|  |  |  |  |  |  |  |  |  |  |  |  | Non-fatal suicidal behaviour: Suicide attempt | | Bivariate | | Alcohol abuse or dependence was associated with a suicide attempt (OR=2·58, 95% CI 1·05-6·34, p<0·05). | | Positive | |  |  |
|  |  |  |  |  |  |  |  |  |  |  |  | Suicidal ideation | | Multivariate | | Alcohol abuse/dependence was not associated with suicidal ideation (aOR=2·42, 95% CI 0·89–6·61, p>0·05), while controlling for drug use/disorder and tobacco use/disorder. | | Null | |  |  |
|  |  |  |  |  |  |  |  |  |  |  |  | Suicidal ideation | | Multivariate | | Alcohol abuse/dependence was not associated with suicide plan (aOR=1·54, 95% CI 0·36-6·50, p>0·05), while controlling for psychiatric disorder and drug use/disorder and tobacco use/disorder. | | Null | |  | |
|  |  |  |  |  |  |  |  |  |  |  |  | Non-fatal suicidal behaviour: Suicide attempt | | Multivariate | | Alcohol abuse/dependence was not associated with suicide attempt (aOR=1·79, 95% CI 0·37-8·65, p>0·05), while controlling for psychiatric disorder and drug use/disorder and tobacco use/disorder. | | Null | |  | |
|  |  | |  |  | |  | |  | |  | |  | |  | |  | |  | |  | |
| **Alcohol** | Abuse | | Pridemore, 2013 [73] | Russia  Other | | Adults (25 to 54 years old) | | Case-control study | | Male (100%) | | Fatal suicide | | Bivariate | | Relative to moderate drinkers, heavier drinkers are not at greater risk of suicide until the 20 (or more) litres category (OR=2·7, 95% CI 1·5–5·0, p<0·05). | | Positive | | + | |
|  |  |  |  |  |  |  |  |  |  |  |  |  |  | Bivariate | | Relative to men who never or almost never drank non-beverage alcohols, men who drank them one to two times and more than three times per week were (OR=3·9, 95% CI 1·3–11·0, p<0·05) and (OR=7·3, 95% CI 4·3–12·5, p<0·05) times more likely to die from suicide, respectively. Note. Non-beverage alcohol refers to e.g. colognes, medicines, cleaning ﬂuids. | | Positive | |  |  |
|  |  |  |  |  |  |  |  |  |  |  |  |  |  | Bivariate | | Problem drinking was associated with suicide (OR=3·7, 95% CI 2·5–5·6, p<0·05). | | Positive | |  |  |
|  |  | |  |  | |  | |  | |  | |  | |  | |  | |  | |  | |
| **Alcohol** | Abuse | | Singh et al, 2013 [145] | India  Other | | Both (13 to 19 years old) | | Cross-sectional study | | Male 114 (52.3%) & female 104 (47.7%) | | Non-fatal suicidal behaviour: Suicide attempt | | Bivariate | | Alcoholism was not associated with suicide attempts (aOR=0·015, 95% CI -0·151-0·121, p=0·829), while controlling for gender, age, depression, symptoms of anxiety and eating disorders. | | Null | | + | |
|  |  | |  |  | |  | |  | |  | |  | |  | |  | |  | |  | |
| **Alcohol** | Abuse | | Toprak et al, 2011 [89] | Turkey  Others | | Both (16 to 22 years old) | | Cross-sectional study | | Male 293 (46.1%) & female 343 (53.9%) | | Non-fatal suicidal behaviour: Self-harm | | Bivariate | | Frequent alcohol consumption was associated with self-harm in the chi-square analysis (OR=4·99, 95% CI 2·13–11·67, p<0·05). | | Positive | | ++ | |
|  |  |  |  |  |  |  |  |  |  |  |  | Suicidal ideation | | Bivariate | | Frequent alcohol consumption was not associated with suicidal ideation in the chi-square analysis (OR=2·52, 95% CI 0·97-6·53, p>0·05). | | Null | |  |  |
|  |  |  |  |  |  |  |  |  |  |  |  | Non-fatal suicidal behaviour: Suicide attempt | | Bivariate | | Frequent alcohol consumption was not associated with suicide attempt (OR=2·55, 95% CI 0·84-7·77, p>0·05). | | Null | |  |  |
|  |  |  |  |  |  |  |  |  |  |  |  | Non-fatal suicidal behaviour: Self-harm | | Multivariate | | Frequent alcohol consumption was not associated with self-harm (OR=2·97, 95% CI 1·04–8·45, p=0·04). | | Positive | |  |  |
|  |  | |  |  | |  | |  | |  | |  | |  | |  | |  | |  | |
| **Alcohol** | Addiction | | Lavania et al, 2012 [77] | India  Hospital based | | Adults (18 to 60 years old) | | Cross-sectional study | | Male 100% | | Non-fatal suicidal behaviour: Deliberate self-harm | | Bivariate | | Alcohol, mean (SD): group I with deliberate self-harm 2·20 (2·81); group II without deliberate self-harm 4·00 (3·30). Those patients without DSH reported a significantly higher mean for alcohol addiction severity compared to patients with DSH (t=-2·28, p<0·03). | | Negative | | - | |
|  |  | |  |  | |  | |  | |  | |  | |  | |  | |  | |  | |
| **Alcohol** | Dependence | | Armstrong et al, 2014 [100] | India  Community-based | | Adults (18 years old and older) | | Cross-sectional study | | Male 100% | | Suicidal ideation | | Multivariate | | Frequency of alcohol use was not associated with suicidal ideation (aOR=1·43, 95% CI 0·82-2·51, p=0·198), while controlling for current age, place of birth, literacy, place slept most of the time in past 3 months, daily income, current marital status, personal rating of relationship with family, beaten up in last 6 months, ever been forced or coerced into sex, rating of overall personal health, no of times injected in past week, length of injecting drug use, depressive symptom severity scale, anxiety symptom severity scale this. | | Null | | ++ | |
|  |  |  |  |  |  |  |  |  |  |  |  | Non-fatal suicidal behaviour: Suicide attempt | | Multivariate | | Frequency of alcohol use was not associated with suicide attempt in the preceding 12 months, among those reporting suicidal ideation (aOR=2·46, 95% CI 0·88-6·91, p=0·084), while controlling for current age, place of birth, literacy, place slept most of the time in past 3 months, daily income, current marital status, personal rating of relationship with family, beaten up in last 6 months, ever been forced or coerced into sex, rating of overall personal health, no of times injected in past week, length of injecting drug use, depressive symptom severity scale, anxiety symptom severity scale this association remained nonsignificant (aOR=2·46, 95% CI 0·88-6·91, p=0·084). | | Null | |  |  |
|  |  | |  |  | |  | |  | |  | |  | |  | |  | |  | |  | |
| **Alcohol** | Dependence | | Borges et al, 2010 [124] | Mexico  National/  regional registers (e.g. not clinical-based registers) | | Adult (18 to 65 years old) | | Cross-sectional study | | N/A | | Non-fatal suicidal behaviour: suicide attempt | | Multivariate | | Among the total sample: lifetime suicide attempt was associated with alcohol abuse or dependence (aOR=6·6, 95% CI 2·8-15·3, p<0·05), while controlling for age, age-squared, sex, cohorts, and initial categories. | | Positive | | ++ | |
|  |  |  |  |  |  |  |  |  |  |  |  | Suicidal ideation | |  |  | Among the total sample: lifetime suicidal ideation was associated with alcohol abuse or dependence (aOR=2·9, 95% CI 1·6-5·2, p<0·05), while controlling for age, age-squared, sex, cohorts, and initial categories. | | Positive | |  |  |
|  |  |  |  |  |  |  |  |  |  |  |  | Suicidal ideation | |  |  | Alcohol abuse or dependence was associated with a plan among ideators (OR=4·6, 95% CI 2·1-10·0, p<0·05). | | Positive | |  |  |
|  |  |  |  |  |  |  |  |  |  |  |  | Non-fatal suicidal behaviour: suicide attempt | |  |  | Alcohol abuse or dependence did was not associated with a planned attempt (OR=2·2, 95% CI 0·8-6·0, p>0·05). | | Null | |  |  |
|  |  |  |  |  |  |  |  |  |  |  |  | Non-fatal suicidal behaviour: suicide attempt | |  |  | Alcohol abuse or dependence was not associated with an unplanned attempt (OR=2·9, 95% CI 0·8-10·8, p>0·05). | | Null | |  |  |
|  |  | |  |  | |  | |  | |  | |  | |  | |  | |  | |  | |
| **Alcohol** | Dependence | | Botega et al, 2010 [90] | Mexico  Hospital-based | | Adult (18 to 63 years old) | | Cross-sectional study | | Male 4336 (56.6%) & female 1883 (43.4%) | | Suicidal ideation | | Bivariate | | Alcohol use disorders (OR=2·3, 95% CI 1·3-3·6, p=0·002) predicted suicidal ideation. | | Positive | | ++ | |
|  |  | |  |  | |  | |  | |  | |  | |  | |  | |  | |  | |
| **Alcohol** | Dependence | | Bromet et al, 2007 [88] | Ukraine  Population-based | | Adult (18 years old and older) | | Cross-sectional study | | Male 123 (31.9%) & female 264 (68.4%) | | Suicidal ideation | | Multivariate | | Alcoholism (aOR=2·0, 95% CI 1·2-3·4, p<·01); and alcohol abuse/dependence (aOR=2·8, 95% CI 1·7–4·7, p<0·01) were associated with suicidal ideation among the total sample, while controlling for sex and person-years and for the other risk factors. | | Positive | | ++ | |
|  |  |  |  |  |  |  |  |  |  |  |  | Suicidal ideation | | Multivariate | | Alcoholism (aOR=1·2, 95% CI 0·6-2·5, p>0·05); and alcohol abuse/dependence (aOR=2·4, 95% CI 1·2-4·9, p<0·05) were associated with suicide plans among ideators, while controlling for sex and person-years and for the other risk factors. | | Positive | |  |  |
|  |  |  |  |  |  |  |  |  |  |  |  | Non-fatal suicidal behaviour: Suicide attempt | | Multivariate | | Alcoholism (aOR=1·2, 95% CI 0·4-3·4, p>0·05); and alcohol abuse/dependence (aOR=2·8, 95% CI 1·3-6·0, p<0·01) were associated with suicide attempt among suicidal ideators, while controlling for sex and person-years and for the other risk factors. | | Positive | |  |  |
|  |  |  |  |  |  |  |  |  |  |  |  | Suicidal ideation | | Multivariate | | Co-morbid alcoholism (aOR=3·4, 95% CI 2·0-5·6), p<0·001) was associated with increased risk of lifetime suicidal ideation, while controlling for person years and gender. | | Positive | |  |  |
|  |  |  |  |  |  |  |  |  |  |  |  | Suicidal ideation | | Multivariate | | Co-morbid alcoholism (aOR=3·6, 95% CI 1·8-7·2, p<0·001) was associated with increased risk of plans among ideators, while controlling for person years and gender. | | Positive | |  |  |
|  |  |  |  |  |  |  |  |  |  |  |  | Non-fatal suicidal behaviour: Suicide attempt | | Multivariate | | Co-morbid alcoholism (aOR=3·6, 95% CI 1·7-7·9, p<0·01) was associated with increased risk of attempts among ideators, while controlling for person years and gender. | | Positive | |  |  |
|  |  | |  |  | |  | |  | |  | |  | |  | |  | |  | |  | |
| **Alcohol** | Dependence | | Cardoso et al, 2008 [142] | Brazil  Hospital-based | | Adult [BD patients with no alcohol comorbidity (N=125)= mean= 43.9, SD=11.92, BD patients with alcohol abuse (N=23) Mean= 37.7 SD= 9.51, BD patients with alcohol dependence (N=30) mean 42.1, SD= 9.40] | | Cross-sectional study | | Male 54 (29%) & female 132 (71%) | | Non-fatal suicidal behaviour: Suicide attempt | | Bivariate | | In the sample of BD outpatients, they found that, for both the comorbid abuse and the dependence groups, there was a higher percentage of consumers in the group of lifetime suicide attempters than in the group of nonattempters: (1) 65·3% of alcohol-abuse patients in the group of attempters, and 34·7% in the group of nonattempters (x^2^=5·39, df=1, p=0·015); and (2) 67·7% of alcohol-dependent patients in the group of attempters, and 32·3% in the group of nonattempters (x^2^=4·13, df=1, p=0·032). | | Positive | | ++ | |
|  |  | |  |  | |  | |  | |  | |  | |  | |  | |  | |  | |
| **Alcohol** | Dependence | | Coêlho et al, 2010 [143] | Brazil  Community based | | Adult (18 years old and older) | | Cross-sectional study | | N/A | | Suicidal ideation: Thoughts of death, Desire of death, Suicide thought | | Bivariate | | Alcohol use disorder was associated with thoughts of death (OR=2, 95% CI 1·5-2·7, p<0·0001), desire of death (OR=2·5, 95% CI 1·7-3·6, p<0·0001), suicide thought (OR=2·9, 95% CI 1·9-4·3, p<0·0001). | | Positive | | ++ | |
|  |  |  |  |  |  |  |  |  |  |  |  | Non-fatal suicidal behaviour: Suicide attempt | | Bivariate | | Alcohol use disorder was associated with suicide attempt (OR=2·5, 95% CI 1·3-4·9, p<0·005). | | Positive | |  |  |
|  |  |  |  |  |  |  |  |  |  |  |  | Suicidal ideation: Thoughts of death, Desire of death, Suicide thought | | Multivariate | | Alcohol use disorder was associated with thoughts of death (aOR=2·7, 95% CI 1·7-4, p<0·0001), desire of death (aOR=2·5, 95% CI 1·5-4·1, p<0·0006), suicide thoughts (aOR=2·5, 95% CI 1·5-4·1, p<0·0005), while controlling for gender, marital status, age group, education level, major depressive disorder, controlled by all demographic variables and the interaction with gender, and considering AUD as effect modification, and two-way interaction of major depressive episode and AUD, and gender with other socio-demographic variables, and major depressive episode and AUD. Also three-way interaction of major depressive episode, AUD, and gender. | | Positive | |  |  |
|  |  |  |  |  |  |  |  |  |  |  |  | Non-fatal suicidal behaviour: Suicide attempt | | Multivariate | | Alcohol use disorder was associated with suicide attempt (aOR=4, 95% CI 1·7-9·6, p<0·002), while controlling for gender, marital status, age group, education level, major depressive disorder, controlled by all demographic variables and the interaction with gender, and considering AUD as effect modification, and two-way interaction of major depressive episode and AUD, and gender with other socio-demographic variables, and major depressive episode and AUD. Also three-way interaction of major depressive episode, AUD, and gender. | | Positive | |  |  |
|  |  | |  |  | |  | |  | |  | |  | |  | |  | |  | |  | |
| **Alcohol** | Dependence | | Evren et al, 2011 [138] | Turkey  Hospital based | | Both | | Cross-sectional study | | Male 100% | | Non-fatal suicidal behaviour: Self-mutilative behaviour | | Multivariate | | Among the total sample (alcohol dependent men with and without PTSD): age at onset of regular alcohol use was associated with self-mutilation (OR=0·942, 95% CI 0·889-0·998, p=0·044). | | Positive | | ++ | |
|  |  |  |  |  |  |  |  |  |  |  |  | Non-fatal suicidal behaviour: Self-mutilative behaviour | | Multivariate | | Among alcohol dependent men without PTSD: age at onset of regular alcohol use (OR=0·912, 95% CI 0·842-0·988, p=0·024), predicted self-mutilation.  No statistical result among men with PTSD were reported. | | Positive | |  |  |
|  |  | |  |  | |  | |  | |  | |  | |  | |  | |  | |  | |
| **Alcohol** | Dependence | | Evren et al, 2008 [139] | Turkey  Hospital based | | Adults (Mean = 43.1; SD=8.3) | | Cohort study | | Male 100% | | Non-fatal suicidal behaviour: Self-mutilative behaviour | | Bivariate | | Number of self-mutilative episodes was associated with early onset of alcohol use (B=-0·174, SE= 0·084, beta=-0·252, t= -2·068, p=0·044). | | Positive | | ++ | |
|  |  |  |  |  |  |  |  |  |  |  |  |  |  |  |  | Early onset of alcohol use was associated with early onset of self-mutilation among male patients with alcohol dependency (B=0·789, SE=0·164, beta=0·551, t=4·82, p <0·001). | | Positive | |  |  |
|  |  | |  |  | |  | |  | |  | |  | |  | |  | |  | |  | |
| **Alcohol** | Dependence | | Hong et al, 2007 [87] | China  Community based | | Adults (Mean = 23.5; SD = 5.1) | | Cross-sectional study | | Female 100% | | Suicidal ideation | | Bivariate | | Alcohol intoxication within the last six month was reported among female sex workers who reported suicidal ideation (47·7% vs. 30·3%, p<0·01). | | Positive | | + | |
|  |  |  |  |  |  |  |  |  |  |  |  | Non-fatal suicidal behaviour: Suicide attempt | | Bivariate | | Alcohol intoxication within the last six month was reported among female sex workers in China who reported suicide attempts (55·3% vs. 30·8%, p<0·005). | | Positive | |  |  |
|  |  | |  |  | |  | |  | |  | |  | |  | |  | |  | |  | |
| **Alcohol** | Dependence (injecting drug users) | | Jin et al, 2013 [74] | China  Clinic-based (e.g. mobile clinic, free clinic) | | Adults (18 to 55 years old) | | Case-control study | | Male 397 (65.4%) & female 210 (34.6%) | | Suicidal ideation | | Multivariate | | Alcohol use disorder was associated with current suicidal ideation (OR=3·44, 95% CI 1·58-23·6, p<0·01). | | Positive | | ++ | |
|  |  | |  |  | |  | |  | |  | |  | |  | |  | |  | |  | |
| **Alcohol** | Dependence | | Neves et al, 2009 [94] | Brazil  Hospital based | | Adults (Mean = 38.1; SD = 12.2) | | Cohort study | | Male 68 (28.5%) & female 171 (58.4%) | | Non-fatal suicidal behaviour: Suicide attempts | | Bivariate | | Comorbid alcoholism was associated with history of suicide attempt (x^2^=12·8, df=1, p=0·001). | | Positive | | + | |
|  |  |  |  |  |  |  |  |  |  |  |  |  |  | Multivariate | | Comorbid alcoholism was not associated with a history of suicide attempts in bipolar patients (aOR=1·36, NO CI, p=0·410), while controlling for confounding factors. | | Null | |  |  |
|  |  |  |  |  |  |  |  |  |  |  |  |  |  | Multivariate | | Comorbid alcoholism was associated with a history of violent suicide attempts in bipolar patients (aOR=3·11, NO CI, p=0·031), while controlling for confounding factors. | | Positive | |  |  |
|  |  | |  |  | |  | |  | |  | |  | |  | |  | |  | |  | |
| **Alcohol** | Dependence | | Neves et al, 2010 [72] | Brazil  Clinic-based (e.g. mobile clinic, free clinic) | | Adult (Non suicide group Mean = 44.6; SD = 13, suicide group Mean = 38.8; SD = 12.1) | | Case-control study | | Male 62 (30.9%) & female 139 (69.1%) | | Non-fatal suicidal behaviour: Suicide attempt | | Bivariate | | Alcoholism was associated with suicide attempt (45·3% vs. 18·8%, p=0·0001). | | Positive | | - | |
|  |  |  |  |  |  |  |  |  |  |  |  |  |  |  |  | Alcohol dependence was associated with violent suicide attempts (63·6% vs. 34%, x^2^=7·23, df=1, p=0·007). | | Positive | |  |  |
|  |  |  |  |  |  |  |  |  |  |  |  |  |  |  |  | Alcohol dependence was not associated with a lifetime history of violent suicide attempts (OR=5·76, NO CI, p=0·059). | | Null | |  |  |
|  |  | |  |  | |  | |  | |  | |  | |  | |  | |  | |  | |
| **Alcohol** | Dependence | | Nock et al, 2009 [78] | Brazil;  Bulgaria;  Colombia;  India;  Lebanon;  Mexico;  Nigeria;  China (Beijing & Shanghai & Shenzhen);  Romania;  South Africa;  Ukraine  Community based | | Adult (18 years old and older) | | Cross-sectional study | | N/A | | Non-fatal suicidal behaviour: Suicide attempt | | Bivariate | | Alcohol abuse or dependency was associated with lifetime suicide attempt (OR=4·4, 95% CI 3·7–5·3, p<0·05). | | Positive | | + | |
|  |  |  |  |  |  |  |  |  |  |  |  | Non-fatal suicidal behaviour: Suicide attempt | | Bivariate | | Alcohol abuse or dependency was associated with suicide attempt (OR=4·8, 95% CI 3·7–6·1, p<0·05). | | Positive | |  |  |
|  |  |  |  |  |  |  |  |  |  |  |  | Non-fatal suicidal behaviour: Suicide attempt | | Multivariate | | Alcohol abuse or dependency was associated with lifetime suicide attempt (aOR=2·1, 95% CI 1·6–2·6, p<0·05), while controlling for age, age-squared, age cohorts, sex, and person-year. | | Positive | |  |  |
|  |  |  |  |  |  |  |  |  |  |  |  | Non-fatal suicidal behaviour: Suicide attempt | | Multivariate | | Alcohol abuse or dependency was associated with lifetime suicide attempt (aOR=2·6, 95% CI 1·9–3·5, p<0·05), while controlling for age, age-squared, age cohorts, sex, and person-year. | | Positive | |  |  |
|  |  |  |  |  |  |  |  |  |  |  |  | Non-fatal suicidal behaviour: Suicide attempt | | Multivariate | | Alcohol abuse or dependency was associated with suicide attempt (aOR=2·5, 95% CI 1·8–3·6, p<0.05), while controlling for age, age-squared, age cohorts, sex, and personyear. | | Positive | |  |  |
|  |  |  |  |  |  |  |  |  |  |  |  | Non-fatal suicidal behaviour: Suicide attempt | | Multivariate | | Alcohol abuse or dependency was associated with suicide attempt (aOR=3·7, 95% CI 2·6–5·4, p<0·05), while controlling for age, age-squared, age cohorts, sex, and person-year. | | Positive | |  |  |
|  |  |  |  |  |  |  |  |  |  |  |  | Non-fata suicidal behaviour: Suicide attempt | | Multivariate | | Among the total sample, alcohol abuse or dependency was associated with suicidal attempt (OR=2·5, 95% CI 2·0–3·2, p<0·05).  Multivariate survival models of associations between type/number of prior DSM-IV disorders and subsequent suicidal behavior—developing countries. | | Positive | |  |  |
|  |  |  |  |  |  |  |  |  |  |  |  | Non-fatal suicidal behaviour: Suicide attempt | | Multivariate | | Among the total sample, Alcohol abuse or dependency was associated with suicide attempt (OR=3·7, 95% CI 2·6–5·4, p<0·05).  Multivariate survival models of associations between type/number of prior DSM-IV disorders and subsequent suicidal behavior—developing countries. | | Positive | |  |  |
|  |  |  |  |  |  |  |  |  |  |  |  | Non-fatal suicidal behaviour: Suicide attempt | | Multivariate | | Among the total sample, alcohol abuse or dependency was associated with suicide attempt (OR=3·7, 95% CI 2·6–5·4, p<0·05).  Multivariate survival models of associations between type/number of prior DSM-IV disorders and subsequent suicidal behavior—developing countries. | | Positive | |  |  |
|  |  |  |  |  |  |  |  |  |  |  |  | Suicidal ideation | | Multivariate | | Among ideators, alcohol abuse or dependency was associated with suicide pan (OR=1·4, 95% CI 1·0–2·0, p<0·05).  Multivariate survival models of associations between type/number of prior DSM-IV disorders and subsequent suicidal behavior—developing countries. | | Positive | |  |  |
|  |  |  |  |  |  |  |  |  |  |  |  | Non-fatal suicidal behaviour: Suicide attempt | | Multivariate | | Among ideators, alcohol abuse or dependency was not associated with a planned attempt (OR=1·4, 95% CI 0·9–2·1).  Multivariate survival models of associations between type/number of prior DSM-IV disorders and subsequent suicidal behavior—developing countries. | | Null | |  |  |
|  |  |  |  |  |  |  |  |  |  |  |  | Non-fatal suicidal behaviour: Suicide attempt | | Multivariate | | Among ideators, alcohol abuse or dependency was associated with an unplanned attempt (OR=1·9, 95% CI 1·1–3·5, p<0·05). | | Positive | |  |  |
|  |  | |  |  | |  | |  | |  | |  | |  | |  | |  | |  | |
| **Alcohol** | Dependence | | Uzun et al, 2009 [141] | Turkey  Hospital based | | Adult (Age Mean = 36.7; SD = 11.8 years old) | | Cohort study | | Male 195 (65%) & female 105 (35%) | | Non-fatal suicidal behaviour: Suicide attempt | | Bivariate | | Attempted suicide was associated with comorbid alcohol abuse or dependence (24·0% vs. 9·2%), (Fishers’ exact test, df=3, p=0·001). | | Positive | | + | |
|  |  | |  |  | |  | |  | |  | |  | |  | |  | |  | |  | |
| **Alcohol** | Dependence | | Yur’yev et al, 2015 [130] | Ukraine  Population based | | N/A | | Cross-sectional study | | N/A | | Fatal suicide | | Multivariate | | A medium-strength (0·3– 0·7) positive, statistically significant association was revealed between the prevalence of drug addiction and the rates of suicide mortality among both males and females. | | Positive | | + | |
|  |  | |  |  | |  | |  | |  | |  | |  | |  | |  | |  | |
| **Alcohol** | Disorder | | Chan et al, 2014 [96] | Malaysia  Clinic-based (e.g. mobile clinic, free clinic) | | Adults (18 to 76 years old) | | Cohort study | | Male 33 (44%) & female 42 (56%) | | Non-fatal suicidal behaviour: Suicide attempt | | Bivariate | | Current alcohol use disorder (OR=3·14, 95% CI 0·83-11·97, p=0·09), previous alcohol use disorder (OR=7, 95% CI 1·14-42·97, p=0·04) were associated with future suicide attempt among depressed inpatients. | | Positive | | ++ | |
|  |  |  |  |  |  |  |  |  |  |  |  | Non-fatal suicidal behaviour: Suicide attempt | |  |  | Current alcohol use disorder (OR=2, 95% CI 0·52-7·76, p=0·32), and previous alcohol use disorder (no odds ratio calculated) was not associated with the transition from suicidal ideation to future suicide attempt among depressed inpatients. | | Null | |  |  |
|  |  | |  |  | |  | |  | |  | |  | |  | |  | |  | |  | |
| **Alcohol** | Disorder | | Nery et al, 2014 [144] | Brazil  Other | | Adults (18 to 74 years old) | | Cohort study | | Male 139 (28.8%) & female 344 (71.2%) | | Non-fatal suicidal behaviour: Suicide attempt | | Bivariate | | History of at least one suicide attempt was associated with lifetime alcohol use disorder diagnosis (OR=1·8, 95% CI 1·07-3·05, p=0·03). | | Positive | | ++ | |
|  |  | |  |  | |  | |  | |  | |  | |  | |  | |  | |  | |
| **Alcohol** | Disorder | | Suttajit et al, 2013 [146] | Thailand  Hospital based | | Adults (18 years old and older) | | Cross-sectional study | | Male 150 (39.2%) & female 233 (60.8%) | | Non-fatal suicidal behaviour: Suicide risk | | Bivariate | | Alcohol use disorders was associated with suicide risk (t=3·11, β=2·65, 95% CI 0·97-4·33, p<0·001). | | Positive | | ++ | |
|  |  | |  |  | |  | |  | |  | |  | |  | |  | |  | |  | |
| **Alcohol** | Disorder | | Yee, Loh, & Ng, 2013 [136] | Malaysia  Hospital based | | Adults (18 to74 years old) | | Cross-sectional study | | Male 139 (28.8%) & female 344 (71.2%) | | Non-fatal suicidal behaviour: Suicide attempt | | Bivariate | | Those with alcohol-use disorder had a significantly higher rate of suicidal attempt when compared to those with no-alcohol use disorder (31·8% vs. 13·1%, p<0·01). | | Positive | | ++ | |
|  |  |  |  |  |  |  |  |  |  |  |  |  |  | Multivariate | | Alcohol-use disorder was associated with suicidal attempt, (OR=3·09, 95% CI 1·75-55·6, p<0·01), while controlling for gender, race, and other substance use. | | Positive | |  |  |
|  |  | |  |  | |  | |  | |  | |  | |  | |  | |  | |  | |
| **Alcohol** | Disorder | | Zhang et al, 2010 [137] | China  National/  regional registers (e.g. not clinical-based registers) | | Adults (34 to 60 years old) | | Cohort study | | Male 100% | | Fatal suicide | | Bivariate | | Previous suicide attempt was associated with alcohol use disorder (OR=1·63, 95% CI 0·92-2·89, p<0·10). | | Positive | | + | |
|  |  |  |  |  |  |  |  |  |  |  |  | Fatal suicide | | Bivariate | | Previous suicide attempt was not associated with acute alcohol use (OR=0·74, 95% CI 0·38-1·42, NS). | | Null | |  |  |
|  |  |  |  |  |  |  |  |  |  |  |  | Fatal suicide | | Bivariate | | Alcohol use disorder suicide males were more likely than the acute alcohol group to have made previous suicide attempts (OR=2·21, 95% CI 1·01-4·84, p<0·05). | | Positive | |  |  |
|  |  |  |  |  |  |  |  |  |  |  |  | Fatal suicide | | Multivariate | | Alcohol use disorder was associated with previous suicide attempts (aOR=1·94, 95% CI=1·94, 1·07–3·53, p<0·005). | | Positive | |  |  |
|  |  |  |  |  |  |  |  |  |  |  |  | Fatal suicide | | Multivariate | | Alcohol use disorder was associated with having made a previous suicide attempt (aOR=2·14, 95% CI 0·96-4·78, p<0·10). | | Positive | |  |  |
|  |  | |  |  | |  | |  | |  | |  | |  | |  | |  | |  | |
| **Alcohol** | Multiple: abuse & dependence | | Chan et al, 2011 [118] | Malaysia  Hospital-based | | Adult (18 to 76 years old) | | Cross-sectional study | | Male 23 (44%) & Female 42 (56%) | | Non-fatal suicidal behaviour: Suicide attempt | | Bivariate | | Current alcohol abuse or dependence was associated with suicide attempt (x^2^=4·164, df=1, p=0·041). | | Positive | | ++ | |
|  |  |  |  |  |  |  |  |  |  |  |  | Non-fatal suicidal behaviour: Suicide attempt | | Bivariate | | Past alcohol abuse or dependence was not associated with suicide attempt (39·1% vs. 21·2, p>0·05). | | Null | |  |  |
|  |  | |  |  | |  | |  | |  | |  | |  | |  | |  | |  | |
| **Alcohol** | Multiple: abuse & dependence | | Lückhoff et al, 2014 [97] | South Africa  Other | | Adults (Mean age years old =35; SD=10.5) | | Cross-sectional study | | Male 784 (80.5%) & females 190 (19.5%) | | Non-fatal suicidal behaviour: Suicide attempt | | Bivariate | | Alcohol abuse (11·8% vs. 13·6%) (x^2^=0·30, p=0·583) and alcohol dependence (5·1% vs. 2·2%) (x^2^=3·54, p=0·060) was associated with suicide attempt. | | Positive | | ++ | |
|  |  |  |  |  |  |  |  |  |  |  |  | Non-fatal suicidal behaviour: Suicide attempt | | Multivariate | | Alcohol dependence was not associated with suicide attempts (OR=1·34, 95% CI 0·85-2·10, p=0·207), while controlling for cannabis use, marital status, global alogia, and bizarre behaviour (lifetime). | | Null | |  |  |
|  |  | |  |  | |  | |  | |  | |  | |  | |  | |  | |  | |
| **Tobacco** |  | |  |  | |  | |  | |  | |  | |  | |  | |  | |  | |
| **Tobacco** | Use | | Ahmad et al, 2014 [45] | Malaysia  School-based | | Adolescents (12 to 17 years old) | | Cross-sectional study | | Male 12486 (49.6%) & female 12688 (50.4 %) | | Suicidal ideation | | Multivariate | | Current smoking was associated with suicidal ideation (aOR=1·63, 95% CI 1·36-1·96, p<0·01), while controlling for the other factors in the model (gender, ethnicity, parental marital status, current smoking, current drinker, were bullied, physically abused at home, verbally abused at home, stress, anxiety, depression, have close friend, supportive peers, parental supervision, parental connectedness, parental bonding, parental respect for privacy). | | Positive | | ++ | |
|  |  | |  |  | |  | |  | |  | |  | |  | |  | |  | |  | |
| **Tobacco** | Use | | Altamura et al, 2007 [84] | Hospital-based | | Adults [South African Attempters = 31.2 (9.0); South African non-attempters = 39.3 (17.7)] | | Cross-sectional study | | Attempters: male 20 (64.5%) & female 11 (35.5%)  Non-attempters: male 3 (50%) & female 3 (50%) | | Non-fatal suicidal behaviour: Suicide attempt | | Bivariate | | Current smoking was not associated with a suicide attempt during the course of their illness among schizophrenia spectrum disorder patients at high risk for suicide (x^2^=0·676, df=1, p>0·4). | | Null | | - | |
|  |  | |  |  | |  | |  | |  | |  | |  | |  | |  | |  | |
| **Tobacco** | Use | | Blum et al, 2012 [46] | Vietnam, and Taiwan  Community based | | Both (15 to 24 years old) | | Cross-sectional study | | Hanoi (Vietnam): male 3 251 (52.5%) & female 2 940 (47.5%)  Taipei (Taiwan): male 2398 (51%) & female 2308 (49%) | | Suicidal ideation | | Bivariate | | Cigarette smoke in the last month, In Hanoi (Vietnam), was not associated with suicidal ideation (OR=1·63, 95% CI 0·54-4·89, p>0·05). | | Null | | ++ | |
|  |  |  |  |  |  |  |  |  |  |  |  | Suicidal ideation | | Bivariate | | Cigarette smoke in the last month, in Taipei, was associated with suicidal ideation (OR=1·70, 95% CI 1·27-2·29, p<0·01). | | Positive | |  |  |
|  |  |  |  |  |  |  |  |  |  |  |  | Non-fatal suicidal behaviour: Suicide attempt | | Bivariate | | Cigarette smoke in the last month, in Hanoi (Vietnam), was not associated with suicide attempts (OR=0·79, 95% CI 0·09-6·59, p>0·05). | | Null | |  |  |
|  |  |  |  |  |  |  |  |  |  |  |  | Non-fatal suicidal behaviour: Suicide attempt | | Bivariate | | Cigarette smoke in the last month (in Taipei) was associated with suicidal attempt (OR=1·88, 95% CI 1·30-2·70, p<0·01). | | Positive | |  |  |
|  |  | |  |  | |  | |  | |  | |  | |  | |  | |  | |  | |
| **Tobacco** | Use | | Dewing et al, 2013 [85] | South Africa  Community based | | Adults (23 to 29 years old) | | Cross-sectional study | | Female 100% | | Non-fatal suicidal behaviour: suicidal ideation, suicide plan, suicide attempt | | Multivariate | | Smoking was not associated with suicidality (aOR=0·48, 95% CI 0·02-10·1, p>0·05), while controlling for age, self-reported diagnosis of HIV that was newly ascertained during the pregnancy, self-reported cigarette smoking status, marital status (married vs. other), employment status (any vs. none), employment status of the baby's father, and type of housing (informal vs. other). | | Null | | ++ | |
|  |  | |  |  | |  | |  | |  | |  | |  | |  | |  | |  | |
| **Tobacco** | Use | | Evren et al, 2014 [80] | Turkey  School-based | | Adolescents (15 to 17 years old) | | Cross-sectional study | | Male 2614 (52.7%) & female 2343 (47.3%) | | Non-fatal suicidal behaviour: Self-mutilation | | Bivariate | | Among 10th grade school pupils, self-mutilation was associated with smoking more frequently:  •never smoked/1-2 times lifetime=10·2%,  •few times a month/week=22·6%  •everyday=44·5%  x^2^=320·00, df=1, p<0·001. | | Positive | | + | |
|  |  |  |  |  |  |  |  |  |  |  |  | Non-fatal suicidal behaviour: suicide attempt | | Bivariate | | Among 10th grade school pupils, suicide attempt was associated with smoking more frequently:  •never smoked/1-2 times lifetime=10·1%,  •few times a month/week=19·7%  •everyday=38·3%  x^2^=225·8, p<0·001 | | Positive | |  |  |
|  |  | |  |  | |  | |  | |  | |  | |  | |  | |  | |  | |
| **Tobacco** | Use | | Khan et al, 2012 [49] | Malaysia  Clinic-based (e.g. mobile clinic, free clinic) | | Both (15 to 84 years old) | | Cross-sectional study | | Male 128 (43%) & female 170 (57%) | | Suicidal ideation | | Bivariate | | Smoking habit was associated with risk of suicidal ideation (OR=7·10, 95% CI 3·94-2·83). | | Positive | | - | |
|  |  |  |  |  |  |  |  |  |  |  |  | Suicidal ideation | | Bivariate | | Smoking habit was associated with risk for suicidal ideation (OR=7·10, 95% CI 3·94-2·83). | | Positive | |  |  |
|  |  | |  |  | |  | |  | |  | |  | |  | |  | |  | |  | |
| **Tobacco** | Use | | Miller et al, 2011 [56] | Mexico  Community based | | Adolescents (12 to 17 years old) | | Cross-sectional study | | N/A | | Suicidal ideation | | Bivariate | | Lifetime use of tobacco (OR=3·76, 95% CI 2·86-4·93, p<0·01), weekly use of tobacco (OR=3·36, 95% CI 2·12-5·31, p<0·01) and daily use of tobacco (OR=2·96, 95% CI 1·85-4·74, p<0·01) was associated with suicidal ideation. | | Positive | | ++ | |
|  |  |  |  |  |  |  |  |  |  |  |  | Suicidal ideation | | Bivariate | | Lifetime use of tobacco (OR=5·27, 95% CI 3·42-8·12, p<0·01), weekly use of tobacco (OR=4·15, 95% CI 2·69-6·40, p<0·01) and daily use of tobacco (OR=3·19, 95% CI 1·98-5·15, p<0·01) was associated with suicide plan. | | Positive | |  |  |
|  |  |  |  |  |  |  |  |  |  |  |  | Non-fatal suicidal behaviour: Suicide attempt | | Bivariate | | Lifetime use of tobacco (OR=5·91, 95% CI 2·72-12·85, p<0·01), weekly use of tobacco (OR=4·92, 95% CI 2·90-8·35, p<0·01) and daily use of tobacco (OR=4·05, 95% CI 2·33-7·05, p<0·01) was associated with suicide attempt. | | Positive | |  |  |
|  |  |  |  |  |  |  |  |  |  |  |  | Suicidal ideation | | Multivariate | | Lifetime use of tobacco (OR=2·16, 95% CI 1·66-2·81, p<0.01), weekly use of tobacco (OR=3·07, 95% CI 1·76-5·35, p<0·01) and daily use of tobacco (OR=2·50, 95% CI 1·30-4·82, p<0·01) was associated with suicidal ideation, while controlling for psychiatric disorder or drug use/disorder or alcohol use/disorder. | | Positive | |  |  |
|  |  |  |  |  |  |  |  |  |  |  |  | Suicidal ideation | | Multivariate | | Lifetime use of tobacco (OR=2·20, 95% CI 1·25-3·87, p<0·01), weekly use of tobacco (OR=4·55, 95% CI 1·96-10·6, p<0.01) and daily use of tobacco (OR=3·75, 95% CI 1·67-8·41, p<0·01) was associated with suicide plan, while controlling for psychiatric disorder or drug use/disorder or alcohol use/disorder. | | Positive | |  |  |
|  |  |  |  |  |  |  |  |  |  |  |  | Non-fatal suicidal behaviour: Suicide attempt | | Multivariate | | Lifetime use of tobacco (OR=2·59, 95% CI 1·21-5·56, p<0·05), weekly use of tobacco (OR=5·74, 95% CI 2·45-13·4, p<0·01) and daily use of tobacco (OR=5·46, 95% CI 1·92-15·51, p<0.01) was associated with suicide attempt, while controlling for psychiatric disorder or drug use/disorder or alcohol use/disorder. | | Positive | |  |  |
|  |  | |  |  | |  | |  | |  | |  | |  | |  | |  | |  | |
| **Tobacco** | Use | | Nojomi et al, 2007 [70] | Iran  Community based | | Both (14 years old and older) | | Cross-sectional study | | Male 809 (35.2%) & Female 1491 (64.8 %) | | Non-fatal Suicidal behaviour: Suicide attempt | | Bivariate | | Tobacco use were higher among suicide attempters than nonattempters (50% vs. 21·9%, p<0·0005) | | Positive | | ++ | |
|  |  | |  |  | |  | |  | |  | |  | |  | |  | |  | |  | |
| **Tobacco** | Use | | Page et al, 2011 [55] | Philippines;  China;  Namibia  School based | | Adolescents (11 to 16 years old) | | Cross-sectional study | | Philippines: male 3094 (43.2%) & female 4188 (56.8%).  China: male 4356 (51.2%) & female 4537 (48.8%).  Namibia: male 2931 (45.2%) & female 3352 (54.8%). | | Suicidal ideation | | Bivariate | | Current smoking was associated with suicide plan across gender and the three countries (Philippine, China, Namibia):  Philippine boys % (n) smoker=25·1 (162); % (n) nonsmoker=17·3 (361); (OR=1·50, 95% CI 1·20-1·88, p<0·05);  Philippine girls % (n) smoker=32·0 (79); % (n) nonsmoker=17·4 (649); (OR=2·27, 95% CI 1·71-3·03, p<0·05);  China boys % (n) smoker=13·2 (59); % (n) nonsmoker= 5·7 (207); (OR= 2·61, 95% CI 1·90-3·60, p<0·05);  China girls % (n) smoker=31·3 (26); % (n) nonsmoker= 8·8 (380); (OR=4·86, 95% CI 3·00-7·89, p<0·05);  Namibia boys % (n) smoker=40·1 (163); % (n) nonsmoker= 22·9 (561); (OR=1·63, 95% CI 1·29-2·04, p<0·05);  Namibia girls % (n) smoker=39·2 (153); % (n) nonsmoker= 28·5 (710); (OR=1·59, 95% CI 1·26-2·00, p<0·05). | | Positive | | ++ | |
|  |  | |  |  | |  | |  | |  | |  | |  | |  | |  | |  | |
| **Tobacco** | Use | | Page et al, 2011 [36] | China; Philippines  School based | | Adolescent (11 to 17 years old) | | Cross-sectional study | | China: male 4356 (51.2%) & female 4537 (48.8%).  Philippines: male 3094 (43.2%) & female 4188 (56.8%). | | Suicidal ideation | | Multivariate | | Smoked cigarettes in past 30 days was associated with suicide ideation (OR=1·11, 95% CI 1·07-1·14, p<0·001). | | Positive | | ++ | |
|  |  |  |  |  |  |  |  |  |  |  |  | Suicidal ideation | | Multivariate | | Among Chinese students: Smoked cigarettes in past 30 days was associated with making a suicide plan (OR=1·81, 95% CI 1·74-1·89, p<0·001). | | Positive | |  |  |
|  |  |  |  |  |  |  |  |  |  |  |  | Suicidal ideation | | Multivariate | | Among Philippine students: Smoked cigarettes in past 30 days was associated with suicide ideation (OR=1·51, 95% CI 1·48-1·54, p<0·001). | | Positive | |  |  |
|  |  |  |  |  |  |  |  |  |  |  |  | Suicidal ideation | | Multivariate | | Among Philippine students: Smoked cigarettes in past 30 days was associated with making a suicide plan (OR=2·45 95% CI 2·41-2·49, p<0·001). | | Positive | |  |  |
|  |  | |  |  | |  | |  | |  | |  | |  | |  | |  | |  | |
| **Tobacco** | Use | | Peltzer, 2009 [67] | Kenya; Namibia; Swaziland;  Uganda; Zambia; Zimbabwe  School based | | Adolescents (13 to 15 years old) | | Cross-sectional study | | N/A | | Suicidal ideation | | Bivariate | | Predict tobacco use:  Suicide ideation: (OR=2·25, 95% CI 2·22–2·78, p<0·001); (aOR=1·55, 95% CI 1·53–1·57, p<0·001)  Suicide plan (OR=2·68, 95% CI 2·65–2·70, p<0·001); (aOR=2·13, 95% CI 2·10–2·16, p<0·001). | | Positive | | + | |
|  |  |  |  |  |  |  |  |  |  |  |  | Suicidal ideation | | Bivariate | | Predict tobacco use:  Suicide plan (OR=2·68, 95% CI 2·65–2·70, p<0·001); (aOR= 2·13 95% CI 2·10–2·16, p<0·001). | | Positive | |  |  |
|  |  | |  |  | |  | |  | |  | |  | |  | |  | |  | |  | |
| **Tobacco** | Use | | Peltzer, 2011 [79] | Botswana  National/regional registers (e.g. not clinical-based registers) | | Adolescents (13 to 15 years old) | | Cross-sectional study | | N/A | | Suicidal ideation | | Multivariate | | Smoking initiation at younger than 14 years old was associated with suicide ideation in the past 12 months among girls (aOR=2·05, 95% CI 1·15-3·66, p<·02) but not boys (No statistical results provided), while controlling for grade, alcohol and other drug use, parental tobacco use, poverty, mental distress, unintentional injuries and violence, ever had sex, physical activity. | | Unclear | | ++ | |
|  |  | |  |  | |  | |  | |  | |  | |  | |  | |  | |  | |
| **Tobacco** | Use | | Peltzer & Louw, 2013 [86] | South Africa  Clinic-based (e.g. mobile clinic, free clinic) | | Adults (18 years old and older) | | Cross-sectional study | | Male 2631 (54.5%) & female 2194 (45.5%) | | Suicidal ideation | | Multivariate | | Current tobacco use was not associated with suicidal ideation (OR=1·19, 95% CI 0·93-1·54, p>0·05). | | Null | | ++ | |
|  |  |  |  |  |  |  |  |  |  |  |  | Non-fatal suicidal behaviour: suicide attempt | | Multivariate | | Current tobacco use was not associated with suicide attempt (OR=1·34, 95% CI 0·91-1·97, p>0·05). | | Null | |  |  |
|  |  | |  |  | |  | |  | |  | |  | |  | |  | |  | |  | |
| **Tobacco** | Use | | Peltzer & Pengpid, 2015 [50] | Oceania (Kiribati,  Samoa, Solomon Islands, and Vanuatu)  School based | | Adolescents (13 to 16 years old) | | Cross-sectional study | | Male 2846 (43.5%) & female 3534 (54%) | | Suicidal ideation | | Bivariate | | Cigarette smoking initiation  was associated with suicidal ideation:  **Among the total sample**  Non-initiators 1·00  <12 years (OR = 2·74, 95% CI 2·12-3·53, p<0·001);  ≥12 years (OR= 2·75, 95% CI 2·19-3·48, p<0·001).  **Among boys**  Non-initiators 1·00  <12 years (OR=2·04, 95% CI 1·4-2.87, p<0.001);  ≥12 years (OR=2·60, 95% CI 1·9-3·49, p<0·001).  AND  **Among girls**  Non-initiators 1·00  <12 years (OR=4·41, 95% CI 2·98-6·52, p<0·001);  ≥12 years (OR=2·84, 95% CI 2·06-3·90, p<0·001). | | Positive | | ++ | |
|  |  |  |  |  |  |  |  |  |  |  |  | Suicidal ideation | | Bivariate | | Current smoking was associated with suicidal ideation (OR=2·73, 95% CI 2·2-3·31, p<0·001).  **Among boys**  (OR=2·48, 95% CI 1·9-3·15, p<0·001);  **Among girls**  (OR=3·08, 95% CI 2·40-3·95, p<0·001). | | Positive | |  |  |
|  |  |  |  |  |  |  |  |  |  |  |  | Non-fatal suicidal behaviour: Suicide attempt | | Bivariate | | Cigarette smoking initiation  was associated with suicide attempt:  **Among the total sample**  Non-initiators 1·00  <12 years (OR=5·81, 95% CI 4·41-7·65, p<0·001);  ≥12 years (OR=3·69, 95% CI 2·75-4·95, p<0·001).  **Among boys**  Non-initiators 1·00  <12 years (OR=5·84, 95% CI 4·15-8·22, p<0·001);  ≥12 years (OR=3·13, 95% CI 2·22-4·41, p<0·001).  AND  **Among girls**  Non-initiators 1·00  <12 years (OR=5·77, 95% CI 3·75-8·88, p<0·001);  ≥12 years OR=4·34, 95% CI 3·26-5·80, p<0·001). | | Positive | |  |  |
|  |  |  |  |  |  |  |  |  |  |  |  | Non-fatal suicidal behaviour: Suicide attempt | | Bivariate | | Current smoking was associated with suicide attempt (OR=4·29, 95% CI 3·41-5·41, p<0·001).  **Among boys**  (OR=4·66, 95% CI 3·25-6·68, p<0·001);  **Among girls**  (OR=4·29, 95% CI 3·39-5·42, p<0·001). | | Positive | |  |  |
|  |  |  |  |  |  |  |  |  |  |  |  | Suicidal ideation | | Multivariate | | Smoking initiation  was associated with suicidal ideation:  **Among the total sample**  Non-initiators 1·00  <12 years (aOR=1·65, 95% CI 1·07-2·53, p<0·05);  Not  ≥12 years (aOR=1·53, 95% CI 0·96-2·42, p>0·05).  **Not among boys**  Non-initiators 1·00  <12 years (aOR=1·04, 95% CI 0·56-1.94, p>0·05);  ≥12 years (aOR=1·08, 95% CI 0·57-2·07, p>0·05).  AND  **Among girls**  Non-initiators 1·00  <12 years (aOR=2·79, 95% CI 1·64-4·76, p<0·001);  ≥12 years (aOR=1·83, 95% CI 1·08-3·10, p<0·05).  (Adjusted for age, psychological distress and current  alcohol use). | | Unclear | |  |  |
|  |  |  |  |  |  |  |  |  |  |  |  | Non-fatal suicidal behaviour: Suicide attempt | | Multivariate | | Smoking initiation  was associated with suicide attempt:  **Among the total sample**  Non-initiators 1·00  <12 years (aOR=2·12, 95% CI 1·40-3·22, p<0·001);  Not  ≥12 years (aOR=1·21, 95% CI 0·78-1·89, p>0·05).  **Among boys**  Non-initiators 1·00  <12 years (aOR=1·87, 95% CI 1·13-2·95, p<·05);  Not  ≥12 years (aOR=0·91, 95% CI 0·52-1·61, p>0·05).  AND  **Among girls**  Non-initiators 1·00  <12 years (aOR=2·64, 95% CI 1·67-4·16, p<0·001);  Not  ≥12 years (aOR=1·56, 95% CI 0·94-2·58, p>0·05).  (Adjusted for age, psychological distress and current  alcohol use). | | Unclear | |  |  |
|  |  | |  |  | |  | |  | |  | |  | |  | |  | |  | |  | |
| **Tobacco** | Use | | Pillai et al, 2009 [64] | India  Community based | | Adolescents (16 to 24 years old) | | Cross-sectional study | | Male 1780 (49.6%) & females 1882 (51.4%) | | Non-fatal suicidal behaviour: Suicide behaviour | | Multivariate | | Tobacco use at least once a week was associated with suicidal thinking/planning/attempts during past 3 months in young adults (aOR=2·3, 95% CI 1·2-4·2, p<0·05). | | Positive | | ++ | |
|  |  |  |  |  |  |  |  |  |  |  |  | Non-fatal suicidal behaviour: Suicide behaviour | | Multivariate | | Tobacco use less than once a week was not associated with suicide behaviour (aOR=0·5, 95% CI 0·1-2·2, p>0·05). | | Null | |  |  |
|  |  | |  |  | |  | |  | |  | |  | |  | |  | |  | |  | |
| **Tobacco** | Use | | Pumariega et al, 2014 [61] | Turkey  School based | | Adolescents (14 to 18 years old) | | Cross-sectional study | | Male 14477 (46.6%) & female 16581 (53.4%) | | Suicidal ideation | | Bivariate | | Suicidal ideation was not associated with tobacco use (OR=1·075, NO CI, p=0·311). | | Null | | + | |
|  |  | |  |  | |  | |  | |  | |  | |  | |  | |  | |  | |
| **Tobacco** | Use | | Randall et al, 2014 [83] | Republic of Benin, West Africa  School based | | Adolescents (11 to 16 years old) | | Cross-sectional study | | Male 1798 (67.1%) & female 882 (32.9%) | | Suicidal ideation | | Bivariate | | Among Benin adolescents who reported tobacco use, 2.7% (54) reported no suicidal ideation, 6.1% (8) reported ideation only, 6.7% (31) reported ideation with a plan, p=0·0004. | | Positive | | ++ | |
|  |  |  |  |  |  |  |  |  |  |  |  | Non-fatal suicidal behaviour: Suicide attempt | | Bivariate | | Tobacco use was associated with number of suicide attempts p<0·0001; no attempt=2·7%(50), one attempt =4%(14), 2 or more attempts =8·4%(29). | | Positive | |  |  |
|  |  |  |  |  |  |  |  |  |  |  |  | Suicidal ideation | | Multivariate | | Tobacco use was not associated with suicidal ideation (OR=0·98, 95% CI 0·37–2·61, p=0·963), while controlling for age, psycho-social symptoms, and socio-environmental factors. | | Null | |  |  |
|  |  |  |  |  |  |  |  |  |  |  |  | Suicidal ideation | | Multivariate | | Tobacco use was not associated with suicidal ideation with a plan (OR=1·24, 95% CI 0·58–2·63, p=0·560), while controlling for age, psycho-social symptoms, and socio-environmental factors. | | Null | |  |  |
|  |  |  |  |  |  |  |  |  |  |  |  | Non-fatal suicidal behaviour: Suicide attempt | | Multivariate | | Tobacco use was not associated with one past suicide attempt (OR=0·64, 95% CI 0·31–1·31, p=0·200), while controlling for age, psycho-social symptoms, and socio-environmental factors. | | Null | |  |  |
|  |  |  |  |  |  |  |  |  |  |  |  | Non-fatal suicidal behaviour: Suicide attempt | | Multivariate | | Tobacco use was not associated with two or more past suicide attempts (OR=0·89, 95% CI 0·39–2·03, p=0·766), while controlling for age, psycho-social symptoms, and socio-environmental factors. | | Null | |  |  |
|  |  | |  |  | |  | |  | |  | |  | |  | |  | |  | |  | |
| **Tobacco** | Use | | Rudatsikira et al, 2007 [68] | Uganda  School based | | Adolescents (11 to 17 years old) | | Cross-sectional study | | Male 784 (53.3 %) & female 676 (46.7%) | | Suicidal ideation | | Bivariate | | Cigarette smoking in the past 12 months was associated with suicidal ideation among the total sample (OR=1·75, 95% CI 1·01-3·04, p<0·05), but not among males (OR=1·2, 95% CI 0·68-1·85, p>0·05) and females respectively (OR=1·81, 95% CI 1·05-3·13, p>0·05). | | Unclear | | - | |
|  |  |  |  |  |  |  |  |  |  |  |  | Suicidal ideation | | Multivariate | | Cigarette smoking in the last 12 months was not associated with suicidal ideation (OR=1·29, 95% CI 0·55-3·01, p>0·05), while controlling for age, gender, loneliness, worry, drinking and being bullied. | | Null | |  | |
|  |  | |  |  | |  | |  | |  | |  | |  | |  | |  | |  | |
| **Tobacco** | Use | | Rudatsikira et al, 2007 [69] | Zimbabwe  School based | | Adolescents (11 to 17 years old) | | Cross-sectional study | | Male 873 (49.3%) & female 1111 (50.7%) | | Suicidal ideation | | Bivariate | | Tobacco cigarette smoking in the past 12 months was associated with suicidal ideation among the total sample (OR=2·34, 95% CI 1·61-3·39, p<0·05), males (OR=2·70, 95% CI 1·61-4·65, p<0·05), and females(OR=1·90, 95% CI 1·07-3·38, p<0·05). | | Positive | | - | |
|  |  | |  |  | |  | |  | |  | |  | |  | |  | |  | |  | |
| **Tobacco** | Use | | Ruengorn et al, 2012 [51] | Thailand  Hospital based | | Both (13 to 60 years old) | | Case-control study | | Male 354 (32.2%) & female 746 (67.8%) | | Non-fatal suicidal behaviour: Suicide attempt | | Bivariate | | Smoking was not associated with suicide attempts (OR=1·38, 95% CI 0·89-2·15, p=0·147). | | Null | | - | |
|  |  | |  |  | |  | |  | |  | |  | |  | |  | |  | |  | |
| **Tobacco** | Use | | Sharma et al, 2015 [58] | Peru  School based | | Adolescents (12 to 18 years old) | | Cross-sectional study | | Male 425 (46.4%) & female 491 (53.6%) | | Suicidal ideation | | Multivariate | | Smoking was associated with suicidal ideation (aOR=1·70, 95% CI 1·08–2·66, p<0·05), while controlling for psychological, socio-environmental, and demographic factors. | | Positive | | ++ | |
|  |  |  |  |  |  |  |  |  |  |  |  | Non-fatal suicidal behaviour: Suicide attempt | | Multivariate | | Smoking was not associated with suicide attempt (aOR=1·41 95% CI 0·89-2·22, p>0·05), while controlling for psychological, and behavioural factors. | | Null | |  |  |
|  |  | |  |  | |  | |  | |  | |  | |  | |  | |  | |  | |
| **Tobacco** | Use | | Shooshtary et al, 2008 [71] | Iran  Community based | | Both (15 years old and older) | | Cross-sectional study | | Male 187 (37.1%) & female 317 (62.9%) | | Non-fatal suicidal behaviour: Suicide attempt | | Bivariate | | Tobacco use was not associated with lifetime suicide attempts (OR=2·3, 95% CI 0·81-7·31, p=0·09). | | Null | | ++ | |
|  |  | |  |  | |  | |  | |  | |  | |  | |  | |  | |  | |
| **Tobacco** | Use | | Silva et al, 2014 [59] | Brazil  School based | | Adolescents (13 to 18 years old) | | Cross-sectional study | | Male 836 (37.9%) & female 1371 (62.1%) | | Suicidal ideation    Non-fatal suicidal behaviour:  Non-fatal: Suicide attempt | | Bivariate | | For cigarette consumption,  association was found with suicide ideation (OR=1·62, CI 95% 1·03-2·55, p<0·05), planning (OR=1·88, CI 95% 1·15-3·08, p<0·05), and attempt (OR = 2·35, CI 95% 1·37 to 4·03). | | Positive | | + | |
|  |  | |  |  | |  | |  | |  | |  | |  | |  | |  | |  | |
| **Tobacco** | Use | | Sitdhiraksa et al, 201) [33] | Thailand  School based | | Both (12 to 21 years old) | | Cross-sectional study | | Male 1054 (40.2%) & female 1568 (59.8%) | | Non-fatal suicidal behaviour: Thoughts of self-harm  Suicide attempt | | Bivariate | | Smoking within the last 12 months was associated with thoughts of harming yourself or attempted suicide (OR=2·04, 95% CI 1·64- 2·54, p<0·01). | | Positive | | + | |
|  |  | |  |  | |  | |  | |  | |  | |  | |  | |  | |  | |
| **Tobacco** | Use | | de Mattos Souza et al, 2010 [38] | Brazil  National/regional registers (e.g. not clinical-based registers) | | Adolescents (11 to 15 years old) | | Cross-sectional study | | Male 501 (48.2%) &  female 538 (51.8%) | | Suicidal ideation | | Multivariate | | Tobacco use was not associated with suicidal ideation (aOR=1·09 95% CI 0·56 - 2·14, p>0·05), while controlling for gender, age, socioeconomic status, level of education, grade retention, religious practice, sexual activity, tobacco use, getting drunk, conduct disorder and high CDI scores for depressive symptoms. | | Null | | ++ | |
|  |  | |  |  | |  | |  | |  | |  | |  | |  | |  | |  | |
| **Tobacco** | Use | | Thakur et al, 2014 [82] | India  School based | | Adolescents (14 to 19 years old) | | Cross-sectional study | | Males 367 (51%) & females 353 (49%) | | Non-fatal suicidal behaviour: Self-harm | | Multivariate | | Self-harm was associated with smoking behaviour (aOR=2·3, 95% CI 1·2-4·4, p<0·05), while adjusting for age, gender, mothers education and fathers education. | | Positive | | - | |
|  |  | |  |  | |  | |  | |  | |  | |  | |  | |  | |  | |
| **Tobacco** | Use | | Wan et al, 2011 [53] | China  School based | | Both (12 to 24 years old) | | Cross-sectional study | | Male 8599 (48.8%) & female 9023 (51.2%) | | Non-fatal suicidal behaviour: Deliberate self-harm | | Bivariate | | Among those with cigarette use, a total of 76·9% reported no DSH, 4.8% reported a single-incident DSH, 18·3% reported repeat-incident DSH. There was a significant difference between those with and those without cigarette use among DSH (x^2^=36·6, df=1, p<0·001). | | Positive | | ++ | |
|  |  |  |  |  |  |  |  |  |  |  |  | Non-fatal suicidal behaviour: Deliberate self-harm | | Bivariate | | Cigarette use did not significantly predict a single-incident DSH (OR=1·07, 95% CI 0·80–1·45, p=0·646), and repeat-incident DSH when controlling for single-incident DSH (OR=1·38, 95% CI 0·99–1·91, p=0·057). | | Null | |  |  |
|  |  | |  |  | |  | |  | |  | |  | |  | |  | |  | |  | |
| **Tobacco** | Use | | Xing et al, 2010 [54] | China  School based | | Adolescents (11 to 19 years old) | | Cross-sectional study | | Males 6216 (49.8%) & female 6254 (50.2%) | | Non-fatal suicidal behaviour: Suicide attempts | | Bivariate | | Suicide attempters were significantly more likely than nonattempters to be current smokers (x^2^=42·45, df=1, p<0·001), and alcohol users (x^2^=83·00, p<0·001). | | Positive | | ++ | |
|  |  | |  |  | |  | |  | |  | |  | |  | |  | |  | |  | |
| **Tobacco** | Use | | Zarrouq et al, 2015 [81] | Morocco  School based | | Adolescents (11 to 23 years old) | | Cross-sectional study | | Males 1602 (53 %) & females 1418 (47 %) | | Suicidal ideation | | Bivariate | | Among smokers, Death wish 34·1% (95% CI 29·8–38·7, p<0·05), Self-harm wish 27% (95% CI 23–31·4, p<0·001), Suicide ideation 25·1% (95% CI 21·2–29·4, p<0·001).  Among nonsmokers, Death wish 25·2% (95% CI 23·5–27, p<0·001), Self-harm wish 12·4, (95% CI 11·1–13·8, p<0·001), Suicide ideation 14% (95% CI 12·6–15·4, p<0·001) . | | Positive | | ++ | |
|  |  |  |  |  |  |  |  |  |  |  |  | Suicidal ideation | | Bivariate | | Among smokers, Suicide plan 10·9% (95% CI 8·2–14·2, p<0·001),  Among nonsmokers, Suicide plan 5·4% (95% CI 4·6–6·4, p<0·001). | | Positive | |  |  |
|  |  |  |  |  |  |  |  |  |  |  |  | Non-fatal suicidal behaviour: Suicide attempts | | Bivariate | | Among smokers, suicide attempt in the past month 12·2% (95% CI 9·4–15·6, p<0·001), Lifetime suicide attempts 16·2 (13–20).  Among nonsmokers, Suicide attempt in the past month 5·4% 95% CI 4·5–6·4, p<0·001), Lifetime suicide attempts 9·5% (95% CI 8·3–10·7, p<0·001). | | Positive | |  |  |
|  |  | |  |  | |  | |  | |  | |  | |  | |  | |  | |  | |
| **Tobacco** | Misuse | | Arenliu et al, 2014 [60] | Kosovo  School-based | | Adolescents (15-19 years old) | | Cross-sectional study | | Male (43.7%) & female (55.1%); not responded (1.2%) | | Suicidal ideation | | Bivariate | | Daily smoking was associated with reported suicidal ideation among males (OR=2·88, 95% CI 1·39–5·66, p<0·05) but not females (OR=1·91, 95% CI 0·48-6·00, p>0·05). No statistics were reported for the total sample. | | Unclear | | - | |
|  |  |  |  |  |  |  |  |  |  |  |  | Non-fatal suicidal behaviour: Suicide attempt | | Bivariate | | Daily smoking was not associated with reported suicide attempt among male (OR=2·23, 95% CI 0·90-5·12, p>0·05) and female adolescents (OR=1·54, 95% CI 0·14-7·88, p>0·05). | | Null | |  |  |
|  |  | |  |  | |  | |  | |  | |  | |  | |  | |  | |  | |
| **Tobacco** | Dependence | | Barbosa et al, 2014 [93] | Brazil  Population-based | | Both (14 to 35 years old) | | Cross-sectional study | | Male 594 (43.0 %) & female 786 (57.0 %) | | Non-fatal suicidal behaviour: Suicide risk (ideation, behaviour) | | Multivariate | | Suicide risk was associated with individuals who smoked when compared to individuals who did not smoke (aOR=2·7, 95% CI 1·8-4, p<0·001), while controlling for gender, age, ethnicity, socioeconomic class, working, marital status, emotional neglect, physical neglect, sexual abuse, physical abuse, emotional abuse. | | Positive | | ++ | |
|  |  | |  |  | |  | |  | |  | |  | |  | |  | |  | |  | |
| **Tobacco** | Dependence | | Botega et al, 2010 [90] | Mexico  Hospital-based | | Adult (18 to 63 years old) | | Cross-sectional study | | Male 4336 (56.6%) & female 1883 (43.4%) | | Suicidal ideation | | Bivariate | | Smoking predicted suicidal ideation (OR=1·9, 95% CI 1·3-2·9, p=0·0008). | | Positive | | ++ | |
|  |  | |  |  | |  | |  | |  | |  | |  | |  | |  | |  | |
| **Tobacco** | Dependence | | Bromet et al, 2007 [88] | Ukraine  Population-based | | Adult (18 years old and older) | | Cross-sectional study | | Male 123 (31.9%) & female 264 (68.4%) | | Suicidal ideation | | Multivariate | | Smoking was not associated with lifetime suicidal ideation among the total sample (aOR=1·7, 95% CI 1·0-3·0, p>0·05), while controlling for sex and person-years and for the other risk factors in each set. | | Null | | ++ | |
|  |  |  |  |  |  |  |  |  |  |  |  | Suicidal ideation | | Multivariate | | Smoking was associated with suicide plans among ideators (aOR=2·3, 95% CI 1·2-4·3, p<0·05), while controlling for sex and person-years and for the other risk factors in each set. | | Positive | |  |  |
|  |  |  |  |  |  |  |  |  |  |  |  | Non-fatal suicidal behaviour: Suicide attempt | | Multivariate | | Smoking was a significant predictor of suicide attempts among ideators (aOR=3·6, 95% CI 1·6-8·0, p<0·01), while controlling for sex and person-years and for the other risk factors in each set. | | Positive | |  |  |
|  |  | |  |  | |  | |  | |  | |  | |  | |  | |  | |  | |
| **Tobacco** | Dependence | | Hong et al, 2007 [87] | Hong  Community based | | Adults (Mean = 23.5; SD = 5.1) | | Cross-sectional study | | Female 100% | | Suicidal ideation | | Bivariate | | Significantly higher proportions of daily smoking in the past 6 months was reported among female sex workers in China who reported suicidal ideation (26·2% vs. 13·6%, p<0·05). | | Positive | | + | |
|  |  |  |  |  |  |  |  |  |  |  |  | Non-fatal suicidal behaviour: Suicide attempt | | Bivariate | | Significantly higher proportions of daily smoking in the past 6 months was reported among female sex workers in China who reported suicide attempts (36·8% vs. 13·5%, p<0·001). | | Positive | |  |  |
|  |  | |  |  | |  | |  | |  | |  | |  | |  | |  | |  | |
| **Tobacco** | Dependence | | Menezes et al, 2012 [92] | Nepal  University students | | Adults (18 to 27 years old) | | Cross-sectional study | | Males 112 (54.4%) & females 94 (45.6%) | | Suicidal ideation | | Bivariate | | A greater proportion of suicidal ideation students reported smoking cigarettes compared to those who did not smoke cigarettes (22·2% vs. 9·6%) but this was not a statistically significant difference (OR=2·69, 95% CI 0·8-9·07, p=0·109). | | Null | | ++ | |
|  |  | |  |  | |  | |  | |  | |  | |  | |  | |  | |  | |
| **Tobacco** | Dependence | | Miller et al, 2011 [56] | Mexico  Community based | | Adolescents (12 to 17 years old) | | Cross-sectional study | | N/A | | Suicidal ideation | | Bivariate | | Tobacco dependence was associated with suicidal ideation (OR=3·79, 95% CI 1·17-12·3, p<0·05). | | Positive | | ++ | |
|  |  |  |  |  |  |  |  |  |  |  |  | Suicidal ideation | | Bivariate | | Tobacco dependence was associated with suicide plan (OR=5·34, 95% CI 1·60-17·8, p<0·01). | | Positive | |  |  |
|  |  |  |  |  |  |  |  |  |  |  |  | Non-fatal Suicidal behaviour: Suicide attempt | | Bivariate | | Tobacco dependence was associated with suicide attempt (OR=7·65, 95% CI 2·05-28·6, p<0·01). | | Positive | |  |  |
|  |  |  |  |  |  |  |  |  |  |  |  | Suicidal ideation | | Multivariate | | Tobacco dependence was associated with suicidal ideation (aOR=4·79, 95% CI 1·05-21·8, p<0·05), while controlling for psychiatric disorder or drug use/disorder or alcohol use/disorder. | | Positive | |  |  |
|  |  |  |  |  |  |  |  |  |  |  |  | Suicidal ideation | | Multivariate | | Tobacco dependence was not associated with suicide plan (OR=1·80, 95% CI 0·15-21·6, p>0·05), while controlling for psychiatric disorder or drug use/disorder or alcohol use/disorder. | | Null | |  |  |
|  |  |  |  |  |  |  |  |  |  |  |  | Non-fatal suicidal behaviour: Suicide attempt | | Multivariate | | Tobacco dependence was associated with suicide attempt (OR=16·4, 95% CI 2·74-98·4, p<0·05), while controlling for psychiatric disorder or drug use/disorder or alcohol use/disorder. | | Positive | |  |  |
|  |  | |  |  | |  | |  | |  | |  | |  | |  | |  | |  | |
| **Tobacco** | Dependence | | Neves et al, 2009 [94] | Brazil  Hospital based | | Adults (Mean = 38.1; SD = 12.2) | | Cohort study | | Male 68 (28.5%) & female 171 (58.4%) | | Non-fatal suicidal behaviour: Suicide attempt | | Bivariate | | Comorbid smoking was associated with history of suicide attempt (x^2^=9·07, df=1, p=0·003). | | Positive | | + | |
|  |  |  |  |  |  |  |  |  |  |  |  | Non-fatal suicidal behaviour: suicide attempt | | Multivariate | | Comorbid smoking was not associated with a history of suicide attempts in bipolar patients (aOR=1·66, NO CI, p=0·114), while controlling for confounding factors. | | Null | |  |  |
|  |  |  |  |  |  |  |  |  |  |  |  | Non-fatal suicidal behaviour: Suicide attempt | | Multivariate | | Comorbid smoking was not associated with a history of violent suicide attempts in bipolar patients (aOR=1·21, NO CI, p=0·693), while controlling for confounding factors. | | Null | |  |  |
|  |  | |  |  | |  | |  | |  | |  | |  | |  | |  | |  | |
| **Tobacco** | Disorder | | Nojomi et al, 2007 [70] | Iran  Community based | | Both (14 years old and older) | | Cross-sectional study | | Male 809 (35.2%) & Female 1491 (64.8 %) | | Non-fatal suicidal behaviour: Suicide attempt | | Bivariate | | Lifelong tobacco use significantly predicted lifelong suicide attempts (OR=3·043, 95% CI 1·67-5·54, p<0·001). | | Positive | | ++ | |
|  |  | |  |  | |  | |  | |  | |  | |  | |  | |  | |  | |
| **Tobacco** | Dependence | | Osama et al, 2014 [91] | Pakistan  Others | | Adults (18 to 29 years old) | | Cross-sectional study | | 135 Males 135 (41.2%) & female 193 (58.8%) | | Suicidal ideation | | Multivariate | | Smoking (90·5% vs. 9·5%) significantly predicted suicidal ideation (aOR=3·18, 95% CI 1·49-6·82, p<0·003), while controlling for parental neglect, demanding parents, dissatisfied with college, ragging, assault, breakup, and psychiatric disorder. | | Positive | | - | |
|  |  | |  |  | |  | |  | |  | |  | |  | |  | |  | |  | |
| **Tobacco** | Dependence | | Toprak et al, 2011 [89] | Turkey  Others | | Both (16 to 22 years old) | | Cross-sectional study | | Male 293 (46.1%) & female 343 (53.9%) | | Non-fatal suicidal behaviour: Self-harm | | Bivariate | | Daily smoking was associated with self-harm (OR=3·19, 95% CI 1·99-5·11, p<0·05). | | Positive | | ++ | |
|  |  |  |  |  |  |  |  |  |  |  |  | Suicidal ideation | | Bivariate | | Daily smoking was associated with suicidal ideation (OR=1·75, 95% CI 1·00–3·06, p<0·05). | | Positive | |  |  |
|  |  |  |  |  |  |  |  |  |  |  |  | Non-fatal suicidal behaviour: Suicide attempt | | Bivariate | | Daily smoking was not associated with suicide attempt (OR=1·75, 95% CI 0·89-3·44, p>0·05). | | Null | |  |  |
|  |  |  |  |  |  |  |  |  |  |  |  | Non-fatal suicidal behaviour: Self-harm | | Multivariate | | Daily smoking was associated with self-harm (OR=2·72, 95% CI 1·46–5·06, p=0·002). | | Positive | |  |  |
|  |  | |  |  | |  | |  | |  | |  | |  | |  | |  | |  | |
| **Tobacco** | Multiple: abuse & dependence | | Chan et al, 2014 [96] | Malaysia  Clinic-based (e.g. mobile clinic, free clinic) | | Adults | | Cohort study | | Male 2407 (47.5%) & Female 2174 (52.5%) | | Non-fatal suicidal behaviour: Suicide attempt | | Bivariate | | Nicotine abuse or dependence was not associated with future suicide attempt among depressed inpatients (OR=2·60, 95% CI 0·72-9·34, p=0·14).  Nicotine abuse or dependence was not associated with the transition from suicidal ideation to future suicide attempt among depressed inpatients (OR=4·13, 95% CI 0·52-7·16, p=0·33). | | Null | | ++ | |
|  |  | |  |  | |  | |  | |  | |  | |  | |  | |  | |  | |
| **Tobacco** | Multiple: use or dependence | | Hooman et al, 2013 [95] | Iran  Hospital based | | Adults (Mean = 45.31; SD=13.7.) | | Cross-sectional study | | Male 695 (73.2%) & female 255 (26.8%) | | Non-fatal suicidal behaviour: Suicide attempt | | Bivariate | | Cigarette smoking was associated with suicide attempt among males (OR=2·24, 95% CI 1·05-4·80, p=0·037), and females (OR=2·09 95% CI 1·29-3·41, p<0·001). | | Positive | | ++ | |
|  |  | |  |  | |  | |  | |  | |  | |  | |  | |  | |  | |
| **Cannabis** |  | |  |  | |  | |  | |  | |  | |  | |  | |  | |  | |
| **Cannabis** | Use | | Arenliu et al, 2014 [60] | Kosovo  School-based | | Adolescents (15-19 years old) | | Cross-sectional study | | Male (43.7%) & female (55.1%); not responded (1.2%) | | Suicidal ideation | | Bivariate | | Cannabis use in the last 30 days for males (OR=3·51, 95% CI 1·51–7·91, p=0·05) and for females (OR=6·79, 95% CI 1·67–25·4, p=0·05) were associated with suicidal ideation. | | Positive | | - | |
|  |  |  |  |  |  |  |  |  |  |  |  | Non-fatal suicidal behaviour: Suicide attempt | | Bivariate | | Cannabis use in the last 30 days for males (OR= 6·17, 95% CI 2·37–15·5, p=0·001) and for females (OR=16·1, 95% CI 3·96–60·98, p=0·001) were associated with suicide attempt. | | Positive | |  |  |
|  |  | |  |  | |  | |  | |  | |  | |  | |  | |  | |  | |
| **Cannabis** | Use | | Muula et al, 2007 [40] | Zambia  School based | | Adolescents (14 to 16 years old) | | Cross-sectional study | | Males 919 (54.0%) & female 964 (46%) | | Suicidal ideation | | Bivariate | | Ever having smoked marijuana was associated with suicidal ideation (OR=1·34, 95% CI 1·33-1·35, p<0·05). | | Positive | | + | |
|  |  | |  |  | |  | |  | |  | |  | |  | |  | |  | |  | |
| **Cannabis** | Use | | Nojomi et al, 2007 [70] | Iran  Community based | | Both (14 years old and older) | | Cross-sectional study | | Male 809 (35.2%) & Female 1491 (64.8 %) | | Non-fatal suicidal behaviour: Suicide attempt | | Bivariate | | Cannabis use were higher among suicide attempters than nonattempters (2·6% vs. 0·50%, p>0·05). | | Null | | ++ | |
|  |  | |  |  | |  | |  | |  | |  | |  | |  | |  | |  | |
| **Cannabis** | Use | | Peltzer, 2008 [66] | South Africa  School based | | Adolescents (15 to 18 years old) | | Cross-sectional study | | Male | | Non-fatal suicidal behaviour: Suicide risk | | Multivariate | | Past month frequency of cannabis use was associated with a higher suicide risk (OR=2·63, 95% CI 1·54-4·49, p<0·001). | | Positive | | ++ | |
|  |  | |  |  | |  | |  | |  | |  | |  | |  | |  | |  | |
| **Cannabis** | Use | | Peltzer & Pengpid, 2015 [50] | Oceania (Kiribati,  Samoa, Solomon Islands, and Vanuatu)  School based | | Adolescents (13 to 16 years old) | | Cross-sectional study | | Male 2846 (43.5%) & female 3534 (54%) | | Suicidal ideation | | Bivariate | | Current cannabis use associated with suicidal ideation (OR=3·18, 95% CI 2·5-3·91, p<0·001). | | Positive | | ++ | |
|  |  |  |  |  |  |  |  |  |  |  |  | Non-fatal suicidal behaviour: Suicide attempt | | Bivariate | | Current cannabis use was associated with suicide attempt (OR=7·33, 95% CI 5·47-9·82, p<0·001). | | Positive | |  | |
|  |  | |  |  | |  | |  | |  | |  | |  | |  | |  | |  | |
| **Cannabis** | Use | | Pumariega et al, 2014 [61] | Turkey  School based | | Adolescents (14 to 18 years old) | | Cross-sectional study | | Male 14 477 (46.6%) & female 16581 (53.4%) | | Suicidal ideation | | Bivariate | | Suicidal ideation was not associated with cannabis/marijuana use (OR=1·22, NO CI, p=0·130). | | Null | | + | |
|  |  | |  |  | |  | |  | |  | |  | |  | |  | |  | |  | |
| **Cannabis** | Use | | Reyes-Tovilla et al, 2015 [57] | Mexico  Hospital based | | Both (13 to 60 years old) | | Cohort study | | Male 52 (36.1%) &  female 92 (63.9%) | | Non-fatal suicidal behaviour: Suicide attempt | | Multivariate | | Intake of cannabis prior to suicide attempt was inversely associated with an impulsive or premeditated suicide attempt (OR=0·25, 95% CI 0·78-0·82, p=0·02), while controlling for past number of attempts, intake of alcohol prior to attempt, cannabis use, alcohol consumption. | | Negative | | + | |
|  |  |  |  |  |  |  |  |  |  |  |  | Non-fatal suicidal behaviour: Suicide attempt | | Multivariate | | Cannabis use was not associated with an impulsive or premeditated suicide attempt (OR=0·64, 95% CI 0·09-3·71, p=0·60), while controlling for past number of attempts, alcohol consumption, intake of alcohol prior to attempt, intake of cannabis prior to attempt. | | Null | |  |  |
|  |  | |  |  | |  | |  | |  | |  | |  | |  | |  | |  | |
| **Cannabis** | Use | | Rudatsikira et al, 2007 [69] | Zimbabwe  School based | | Adolescents (11 to 17 years old) | | Cross-sectional study | | Male 873 (49.3%) & female 1111 (50.7%) | | Suicidal ideation | | Bivariate | | Cannabis smoking in the past 12 months was associated with suicidal ideation among the total sample (OR=1·50, 95% CI 1·04-2·16, p<0·05), and males (OR=1·65, 95% CI 1·03-2·64, p<0·05), but not females (OR=1·35, 95% CI 0·71-2·57, p>0·05). | | Unclear | | - | |
|  |  | |  |  | |  | |  | |  | |  | |  | |  | |  | |  | |
| **Cannabis** | Use | | Silva et al, 2014 [59] | Brazil  School based | | Adolescents (13 to 18 years old) | | Cross-sectional study | | Male 836 (37.9%) & female 1371 (62.1%) | | Suicidal ideation | | Bivariate | | Marijuana consumption was not associated with suicidal ideation, suicide planning or suicide attempt: No statistical results provided. | | Null | | + | |
|  |  |  |  |  |  |  |  |  |  |  |  | Suicidal ideation | | Bivariate | | Marijuana consumption was not associated with suicidal ideation, suicide planning or suicide attempt: No statistical results provided. | | Null | |  |  |
|  |  |  |  |  |  |  |  |  |  |  |  | Non-fatal suicidal behaviour: Suicide attempt | | Bivariate | | Marijuana consumption was not associated with suicidal ideation, suicide planning or suicide attempt: No statistical results provided. | | Null | |  |  |
|  |  | |  |  | |  | |  | |  | |  | |  | |  | |  | |  | |
| **Cannabis** | Abuse | | Toprak et al, 2011 [89] | Turkey  Others | | Both (16 to 22 years old) | | Cross-sectional study | | Male 293 (46.1%) & female 343 (53.9%) | | Non-fatal suicidal behaviour: Self-harm | | Bivariate | | Cannabis abuse was significantly associated with self-harm (aOR=6·65, 95% CI 2·54-17·4, p<0·05), while controlling for age and gender. | | Positive | | ++ | |
|  |  |  |  |  |  |  |  |  |  |  |  | Suicidal ideation | | Bivariate | | Cannabis abuse was not significantly associated with suicidal ideation (OR=0·84, 95% CI 0·19-3·73, p>0·05). | | Null | |  |  |
|  |  |  |  |  |  |  |  |  |  |  |  | Non-fatal suicidal behaviour: Suicide attempt | | Bivariate | | Cannabis abuse was not significantly associated with suicide attempt (OR=1·60, 95% CI 0·35-7·20, p>0·05). | | Null | |  |  |
|  |  | |  |  | |  | |  | |  | |  | |  | |  | |  | |  | |
| **Cannabis** | Composite substance measure: use, abuse, dependence | | Lückhoff et al, 2014 [97] | South Africa  Other | | Adults (Mean age years old =35; SD=10.5) | | Cross-sectional study | | Male 784 (80.5%) & females 190 (19.5%) | | Non-fatal suicidal behaviour: Suicide attempt | | Bivariate | | When the suicide group was compared to the group without suicide: cannabis use or abuse (31·3% vs. 27·2) (x^2^=1·02, p=0.0313); or dependency (11% vs. 4·7%) (x^2^=8·49, p=0·004); use or abuse or dependence (47·5% vs. 35·5%) (x^2^=7·15, p=0·008) was risk factors for suicidal behaviour. | | Positive | | ++ | |
|  |  |  |  |  |  |  |  |  |  |  |  | Non-fatal suicidal behaviour: Suicide attempt | | Multivariate | | Cannabis use or abuse or dependence was associated with suicide attempts (aOR=1·22, 95% CI 1·01-1·47, p=0·038), while controlling for alcohol dependence, marital status, global alogia, and bizarre behaviour (lifetime). | | Positive | |  |  |
|  |  | |  |  | |  | |  | |  | |  | |  | |  | |  | |  | |
| **Cannabis and Mandrax (consumed together)** |  | |  |  | |  | |  | |  | |  | |  | |  | |  | |  | |
| **Cannabis and Mandrax (consumed together)** | Use | | Peltzer, 2008 [66] | South Africa  School based | | Adolescents (15 to 18 years old) | | Cross-sectional study | | Male | | Non-fatal suicidal behaviour: Suicide risk | | Multivariate | | Past month frequency of cannabis & mandrax use predicted higher suicide risk (aOR=8·35, 95% CI 2·58-26·99, p<0·001), while controlling for age and gender. | | Positive | | ++ | |
|  |  | |  |  | |  | |  | |  | |  | |  | |  | |  | |  | |
| **Opioid** |  | |  |  | |  | |  | |  | |  | |  | |  | |  | |  | |
| **Opioid** | Use | | Juan et al, 2015 [98] | China  School based | | Adolescents (12 to 19 years old) | | Cross-sectional study | | Male 37,753 (45.3%) & female 45,523 (54.7%) | | Non-fatal suicidal behaviour: Deliberate self-harm | | Multivariate | | Lifetime, past-year, and past-month non-medical use of opioids was associated with DSH across gender, when adjusting for family economic status, and the educational background of parents:  **Among males:**  Lifetime (aOR=1·59, 95% CI 1·38–1·83, p<0·05), past-year (aOR=1·89, 95% CI 1·42–2·52, p<0·05), and past-month (aOR=3·76, 95% CI 3.05–4·64, p<0·05) non-medical use of opioids.  **Among females:**  Lifetime (aOR=2·25, 95% CI 1·97-2·56, p<0·05), past-year (aOR=2·66, 95% CI 2·00-3·55, p<0·05), and past-month (aOR=4·32, 95% CI 3·44-5·41, p<0·05) non-medical use of opioids. | | Positive | | ++ | |
|  |  |  |  |  |  |  |  |  |  |  |  | Suicidal ideation | | Multivariate | | Lifetime, past-year, and past-month non-medical use of opioids was associated with suicidal ideation across gender, when adjusting for family economic status, and the educational background of parents:  **Among males:**  Lifetime (aOR=1·34, 95% CI 1·17-1·55, p<0·05), past-year (aOR=1·81, 95% CI 1·37-2·39, p<0·05), and past-month (aOR=2·49, 95% CI 2·00-3·11, p<0·05) non-medical use of opioids.  **Among females:**  Lifetime (aOR=1·41, 95% CI 1·25-1·60, p<0·05), past-year (aOR=1·48, 95% CI 1·13-1·95, p<0·05), and past-month (aOR=1·83, 95% CI 1·45-2·31, p<0·05) non-medical use of opioids. | | Positive | |  |  |
|  |  |  |  |  |  |  |  |  |  |  |  | Non-fatal suicidal behaviour: Suicide attempt | | Multivariate | | Lifetime, past-year, and past-month non-medical use of opioids was associated with suicide attempt across gender, when adjusting for family economic status, and the educational background of parents:  **Among males:**  Lifetime (aOR=2·05, 95% CI 1·60-2·63, p<0·05), past-year (aOR=3·33, 95% CI 2·18-5·08, p<0·05), and past-month (aOR=8·97, 95% CI 6·91-11·7, p<0·05) non-medical use of opioids.  **Among females:**  Lifetime (aOR=2·54, 95% CI 2·07-3·13, p<0·05), past-year (aOR=2·78, 95% CI 1·77-4·36, p<0·05), and past-month (aOR=4·79, 95% CI 3·49-6·56, p<0·05) non-medical use of opioids. | | Positive | |  |  |
|  |  | |  |  | |  | |  | |  | |  | |  | |  | |  | |  | |
| **Opioid** | Use | | Nojomi et al, 2007 [70] | Iran  Community based | | Both (14 years old and older) | | Cross-sectional study | | Male 809 (35.2%) & Female 1491 (64.8 %) | | Non-fatal suicidal behaviour: Suicide attempt | | Bivariate | | Heroin / morphine use were higher among suicide attempters than nonattempters (39·5% vs. 36·2%, p>0·05). | | Null | | ++ | |
|  |  | |  |  | |  | |  | |  | |  | |  | |  | |  | |  | |
| **Opioid** | Use | | Peltzer, 2008 [66] | South Africa  School based | | Adolescents (15 to 18 years old) | | Cross-sectional study | | Male | | Non-fatal suicidal behaviour: Suicide risk | | Multivariate | | Higher past month frequency of opiate use predicted higher suicide risk (OR=5·28, 95% CI 1·20-23·3, p<0·05), while controlling for age and gender. | | Positive | | ++ | |
|  |  | |  |  | |  | |  | |  | |  | |  | |  | |  | |  | |
| **Opioid** | Use | | Shooshtary et al, 2008 [71] | Iran  Community based | | Both (15 years old and older) | | Cross-sectional study | | Male 187 (37.1%) & female 317 (62.9%) | | Non-fatal suicidal behaviour: Suicide attempt | | Bivariate | | Opioid use was not associated with lifetime suicide attempts (OR=1·05, 95% CI 0·007-163·8, p>0·05). | | Null | | ++ | |
|  |  | |  |  | |  | |  | |  | |  | |  | |  | |  | |  | |
| **Opioid** | Dependence | | Ahmadi et al, 2015 [99] | Iran  Hospital-based | | Both (16 to 25 years old) | | Case-control study | | Male (24%) & female (76%) | | Non-fatal suicidal behaviour: Suicide attempt | | Multivariate | | Opium dependence was a significant risk factor associated with self-immolation (aOR=17·33, 95% CI 5·40–55·6, p<0·001), while controlling for adjustment disorders, major depression, individual history of suicide attempts. | | Positive | | ++ | |
|  |  | |  |  | |  | |  | |  | |  | |  | |  | |  | |  | |
| **Sedatives** |  | |  |  | |  | |  | |  | |  | |  | |  | |  | |  | |
|  | Use | | Arenliu et al, 2014 [60] | Kosovo  School-based | | Adolescents (15-19 years old) | | Cross-sectional study | | Male (43.7%) & female (55.1%); not responded (1.2%) | | Suicidal ideation | | Bivariate | | The reported usage of tranquilizers was associated with reported suicide ideation for females (OR=9·39, 95% CI 5·28–16·63, p=0·001) but not for males (OR=1·15, 95% CI 0·39-2·97, p>0·05). | | Unclear | | - | |
|  |  |  |  |  |  |  |  |  |  |  |  | Non-fatal suicidal behaviour: suicide attempt | | Bivariate | | Tranquillizers were associated with reported suicide attempts for both males (OR=2·82, 95% CI 0·95–7·40, p<0·001) and females (OR=4·69, 95% CI 1·81–10·6, p<0·05). | | Positive | |  |  |
|  |  | |  |  | |  | |  | |  | |  | |  | |  | |  | |  | |
| **Sedatives** | Use | | Juan et al, 2015 [98] | China  School based | | Adolescents (12 to 19 years old) | | Cross-sectional study | | Male 37,753 (45.3%) & female 45,523 (54.7%) | | Non-fatal suicidal behaviour: Deliberate self-harm | | Multivariate | | Lifetime, past-year, and past-month non-medical use of sedatives was associated with DSH across gender, when adjusting for family economic status, and the educational background of parents:  **Among males:**  Lifetime (aOR=1·70, 95% CI 1·41-2·06, p<0·05), past-year (aOR=2·64, 95% CI 1·84-3·78, p<0·05), and past-month (aOR=5·80, 95% CI 4·53-7·43, p<0·05) non-medical use of sedatives.  **Among females:**  Lifetime (aOR=2·40, 95% CI 2·06-2·81, p<0·05), past-year (aOR=3·03, 95% CI 2·30-4·00, p<0·05), and past-month (aOR=3·67, 95% CI 2·83-4·77, p<0·05) non-medical use of sedatives. | | Positive | | ++ | |
|  |  |  |  |  |  |  |  |  |  |  |  | Suicidal ideation | | Multivariate | | Lifetime, past-year, and past-month non-medical use of sedatives was associated with suicidal ideation across gender, when adjusting for family economic status, and the educational background of parents:  **Among males:**  Lifetime (aOR=1·53, 95% CI 1·31-1·90, p<0·05), past-year (aOR=1·89, 95% CI 1·30-2·75, p<0·05), and past-month (aOR=2·85, 95% CI 2·19-3·71, p<0·05) non-medical use of sedatives.  **Among females:**  Lifetime (aOR=1·57, 95% CI 1·33-1·77, p<0·05), past-year (aOR=1·58, 95% CI 1·21-2·08, p<0·05), and past-month (aOR=2·18, 95% CI 1·68-2·82, p<0·05) non-medical use of sedatives. | | Positive | |  |  |
|  |  |  |  |  |  |  |  |  |  |  |  | Non-fatal suicidal behaviour: Suicide attempt | | Multivariate | | Lifetime, past-year, and past-month non-medical use of sedatives was associated with suicide attempt across gender, when adjusting for family economic status, and the educational background of parents:  **Among males:**  Lifetime (aOR=2·57, 95% CI 1·90-3·48, p<0·05), past-year (aOR=4·26, 95% CI 2·57-7·06, p<0·05), and past-month (aOR=10·8, 95% CI 8·02-14·4, p<0·05) non-medical use of sedatives.  **Among females:**  Lifetime (aOR=2·92, 95% CI 2·31-3·70, p<0·05), past-year (aOR=3·36, 95% CI 22·23-5·04, p<0·05), and past-month (aOR=5·19, 95% CI 3·69-7·29, p<0·05) non-medical use of sedatives. | | Positive | |  |  |
|  |  | |  |  | |  | |  | |  | |  | |  | |  | |  | |  | |
| **Sedatives** | Use | | Peltzer, 2008 [66] | South Africa  School based | | Adolescents (15 to 18 years old) | | Cross-sectional study | | Male | | Non-fatal suicidal behaviour: Suicide risk | | Multivariate | | Higher past month frequency of tranquilizer use predicted higher suicide risk (OR=6·32, 95% CI 1·62-24·7, p<0·01) when controlling for age and gender. | | Positive | | ++ | |
|  |  | |  |  | |  | |  | |  | |  | |  | |  | |  | |  | |
| **Sedatives** | Use | | Shooshtary et al, 2008 [100] | Iran  Community based | | Both (15 years old and older) | | Cross-sectional study | | Male 187 (37.1%) & female 317 (62.9%) | | Non-fatal suicidal behaviour: Suicide attempt | | Bivariate | | Sedatives use did not significantly predicted lifetime suicide attempts (OR=1·1 95% CI 0·86-8·27, p>0·05). | | Null | | ++ | |
|  |  | |  |  | |  | |  | |  | |  | |  | |  | |  | |  | |
| **Sedatives** | Use | | Tran Thi Thanh et al, 2006 [52] | Vietnam  Community based | | Both (14 to 65 years old) | | Cross-sectional study | | Male 1093 (48.4%) & female 1167 (51.6%) | | Suicide ideation: Suicidal thoughts | | Multivariate | | Having ever used sedatives was associated with suicidal thoughts (aOR=2·7, 95% CI 1·7-4·3, p<0·01), while controlling for age, gender, marital status, education level, income, religion, religiousness, employment status, use of alcohol, sedatives, and pain relief medication. | | Positive | | ++ | |
|  |  | |  |  | |  | |  | |  | |  | |  | |  | |  | |  | |
| **Sedatives** | Abuse | | Toprak et al, 2011 [89] | Turkey  Others | | Both (16 to 22 years old) | | Cross-sectional study | | Male 293 (46.1%) & female 343 (53.9%) | | Non-fatal suicidal behaviour: Self-harm | | Bivariate | | Tranquilizer abuse (OR=7·53, 95% CI 3·32–17·1, p<0·05) was associated with self-harm. | | Positive | | ++ | |
|  |  |  |  |  |  |  |  |  |  |  |  | Suicidal ideation | | Bivariate | | Tranquilizer abuse (OR=4·60, 95% CI 2·09–10·1, p<0·05) was associated with suicide ideation. | | Positive | |  |  |
|  |  |  |  |  |  |  |  |  |  |  |  | Non-fatal suicidal behaviour: Suicide attempt | | Bivariate | | Tranquilizer abuse (OR=5·43, 2·24–13·2, p<0·05) was associated with suicide attempt. | | Positive | |  |  |
|  |  |  |  |  |  |  |  |  |  |  |  | Non-fatal suicidal behaviour: Self-harm | | Multivariate | | Tranquilizer abuse (yes/no) (OR=5·51, 95% CI 2·02–15·05, p=0·001) predicted self-harm. | | Positive | |  |  |
|  |  |  |  |  |  |  |  |  |  |  |  | Suicidal ideation | | Multivariate | | Tranquilizer abuse (yes/no) predicted suicidal ideation (OR=4·79, 95% CI 2-11·5, p=0·000). | | Positive | |  |  |
|  |  |  |  |  |  |  |  |  |  |  |  | Non-fatal suicidal behaviour: Suicide attempt | | Multivariate | | Tranquilizer abuse (yes/no) predicted suicide attempt (OR=6·03, 95% CI 2·35-15·5, p=0·000). | | Positive | |  |  |
|  |  | |  |  | |  | |  | |  | |  | |  | |  | |  | |  | |
| **Stimulants** |  | |  |  | |  | |  | |  | |  | |  | |  | |  | |  | |
| **Stimulants** | Use | | Du et al, 2014 [101] | China  Clinic-based (e.g. mobile clinic, free clinic) | | Both (16 to 60 years old) | | Cross-sectional study | | Males 305 (81.1) & females 71 (18.9) | | Non-fatal suicidal behaviour: Suicide behaviour | | Multivariate | | Participants who ever had suicide behaviour were associated with a higher likelihood of being in the high ATS use trajectory group (OR= 3·30, 95% CI 1·35-8·06, p<0·005). | | Positive | | ++ | |
|  |  | |  |  | |  | |  | |  | |  | |  | |  | |  | |  | |
| **Stimulants** | Use | | Narvaez et al, 2014 [102] | Brazil  National/regional registers (e.g. not clinical-based registers) | | Young adults (18 to 24 years old) | | Cross-sectional study | | N/A | | Non-fatal suicidal behaviour: Suicide risk | | Multivariate | | Crack use was associated with suicide risk (OR=3·03, 95% CI 1·22 –7·50, p=0·017), while controlling for lifetime cocaine use. | | Positive | | ++ | |
|  |  | |  |  | |  | |  | |  | |  | |  | |  | |  | |  | |
| **Stimulants** | Use | | Nojomi et al, 2007 [70] | Iran  Community based | | Both (14 years old and older) | | Cross-sectional study | | Male 809 (35.2%) & Female 1491 (64.8 %) | | Non-fatal suicidal behaviour: Suicide attempt | | Bivariate | | ATS use was higher among suicide attempters than nonattempters (1·3% vs. 0·3%, p>0·05) | | Null | | ++ | |
|  |  | |  |  | |  | |  | |  | |  | |  | |  | |  | |  | |
| **Stimulants** | Use | | Peltzer, 2008 [66] | South Africa  School based | | Adolescents (15 to 18 years old) | | Cross-sectional study | | Male | | Non-fatal suicidal behaviour: Suicide risk | | Multivariate | | Higher past month frequency of cocaine use predicted higher suicide risk (OR=8·35, 95% CI 2·58-26·99, p<0·05), while controlling for age and gender. | | Positive | | ++ | |
|  |  | |  |  | |  | |  | |  | |  | |  | |  | |  | |  | |
| **Unspecified prescription medication** |  | |  |  | |  | |  | |  | |  | |  | |  | |  | |  | |
| **Unspecified prescription medication** | Use | | Guo et al, 2015 [103] | China  School based | | Adolescents (Mean = 16.7; SD = 1.2) | | Cross-sectional study | | Male 5465 (45.9%) & female 6441 (54.1%) | | Suicidal ideation | | Multivariate | | Considered suicide behaviour was associated with non-medical use of prescription pain relievers (OR=3·47, 95% CI 2·34-5·15, p<0·05). | | Positive | | ++ | |
|  |  |  |  |  |  |  |  |  |  |  |  | Non-fatal suicidal behaviour: suicide behaviour | | Multivariate | | Attempted suicidal behaviour was associated with non-medical use of prescription pain relievers (OR=2·08, 95% CI 1·29-3·35, p<0·05). | | Positive | |  |  |
|  |  | |  |  | |  | |  | |  | |  | |  | |  | |  | |  | |
| **Unspecified prescription medication** | Use | | Peltzer, 2008 [66] | South Africa  School based | | Adolescents (15 to 18 years old) | | Cross-sectional study | | Male | | Non-fatal suicidal behaviour: Suicide risk | | Multivariate | | Higher past month frequency of over-the-counter drug use predicted higher suicide risk (OR=2·20, 95% CI 1·07-4·51, p<0·01), while controlling for age and gender. | | Positive | | ++ | |
|  |  | |  |  | |  | |  | |  | |  | |  | |  | |  | |  | |
| **Unspecified prescription medication** | Use | | Tran Thi Thanh et al, 2006 [52] | Vietnam  Community based | | Both (14 to 16 years old) | | Cross-sectional study | | Male 1093 (48.4%) & female 1167 (51.6%) | | Suicidal ideation: Suicidal thoughts | | Multivariate | | Having ever used pain relief medication was associated with suicidal thoughts respectively (aOR=2·6, 95% CI 1·6-4·1, p<0·01), while controlling for age, gender, marital status, education level, income, religion, religiousness, employment status, use of alcohol, sedatives, and pain relief medication. | | Positive | | ++ | |
|  |  | |  |  | |  | |  | |  | |  | |  | |  | |  | |  | |
| **Inhalants** |  | |  |  | |  | |  | |  | |  | |  | |  | |  | |  | |
| **Inhalants** | Use | | Peltzer, 2008 [66] | South Africa  School based | | Adolescents (15 to 18 years old) | | Cross-sectional study | | Male | | Non-fatal suicidal behaviour: Suicide risk | | Multivariate | | Higher past month frequency of inhalant use was associated with higher suicide risk (aOR=5·41, 95% CI 1·30-22·5, p<0·05), while controlling for age and gender. | | Positive | | ++ | |
|  |  | |  |  | |  | |  | |  | |  | |  | |  | |  | |  | |
| **Inhalants** | Abuse | | Toprak et al, 2011 [89] | Turkey  Others | | Both (16 to 22 years old) | | Cross-sectional study | | Male 293 (46.1%) & female 343 (53.9%) | | Non-fatal suicidal behaviour: Self-harm | | Bivariate | | Inhalant abuse was associated with self-harm (OR=8·05, 95% CI 3·32-19·5, p<0·05). | | Positive | | ++ | |
|  |  |  |  |  |  |  |  |  |  |  |  | Suicidal ideation | | Bivariate | | Inhalant abuse was not associated with suicidal ideation (OR=0·64, 95% CI 0·14-2·82, p>0·05). | | Null | |  |  |
|  |  |  |  |  |  |  |  |  |  |  |  | Non-fatal suicidal behaviour: Suicide attempt | | Bivariate | | Inhalant abuse was not associated with suicide attempt (OR=0·53, 95% CI 0·07-4·09, p>0·05). | | Null | |  |  |
|  |  |  |  |  |  |  |  |  |  |  |  | Non-fatal suicidal behaviour: Self-harm | | Multivariate | | Inhalant abuse was associated with self-harm (aOR=4·51, 95% CI 1·49-13·7, p<0·008), while controlling for income, good family relationships, frequent alcohol consumption, daily smoking, and tranquilizer abuse. | | Positive | |  |  |
|  |  | |  |  | |  | |  | |  | |  | |  | |  | |  | |  | |
| **Hallucinogens** |  | |  |  | |  | |  | |  | |  | |  | |  | |  | |  | |
| **Hallucinogens** | Use | | Nojomi et al, 2007 [70] | Iran  Community based | | Both (14 years old and older) | | Cross-sectional study | | Male 809 (35.2%) & Female 1491 (64.8 %) | | Non-fatal suicidal behaviour: Suicide attempt | | Bivariate | | Hallucinogens use were higher among suicide attempters than nonattempters (1·3% vs. 0·1%, p>0·05) | | Null | | ++ | |
| **Hallucinogens** | Use | | Peltzer, 2008 [66] | South Africa  School based | | Adolescents (15 to 18 years old) | | Cross-sectional study | | Male | | Non-fatal suicidal behaviour: suicide risk | | Multivariate | | Higher past month frequency of hallicinogens use predicted higher suicide risk (OR=4·76, 95% CI 1·61-14·1, p<0·05), while controlling for age and gender. | | Positive | | ++ | |
|  |  | |  |  | |  | |  | |  | |  | |  | |  | |  | |  | |
| **Unspecified substance** |  | |  |  | |  | |  | |  | |  | |  | |  | |  | |  | |
| **Unspecified substance)** | Intoxication | | Evren et al, 2012 [104] | Turkey  Hospital based | | N/A | | Cross-sectional study | | Male 100% | | Non-fatal suicidal behaviour: Self-mutilation | | Bivariate | | Substance intoxication before the act was associated with self-mutilation. The rate of intoxication before self-mutilation was higher among alcohol dependents when compared to drug dependents (79·6% vs. 54·7%) (x^2^=8·04, df=1, p=0·005). | | Positive | | ++ | |
|  |  | |  |  | |  | |  | |  | |  | |  | |  | |  | |  | |
| **Unspecified substance** | Use | | Arenliu et al, 2014 [60] | Kosovo  School-based | | Adolescents (15-19 years old) | | Cross-sectional study | | Male (43.7%) & female (55.1%); not responded (1.2%) | | Suicidal ideation | | Bivariate | | Usage of drugs other than cannabis (amphetamines, hallucinogens or ecstasy) was associated with reported suicide ideation for males (OR=4·78, 95% CI 2·15–10·18, p<0·001) but not for females (OR=0·67, 95% CI 0·18-2·1, p>0·05). | | Unclear | | - | |
|  |  |  |  |  |  |  |  |  |  |  |  | Non-fatal suicidal behaviour: suicide behaviour | | Bivariate | | The usage of drugs other than cannabis and tobacco was not associated with reported suicide attempts for males (OR=2·26, 95% CI 0·79-5·88, p>0·05) and females (OR=1·05, 95% CI 0·17-4·44, p>0·05). | | Null | |  |  |
|  |  | |  |  | |  | |  | |  | |  | |  | |  | |  | |  | |
| **Unspecified substance** | Use | | Chan et al, 2013 [47] | Malaysia  National/regional registers (e.g. not clinical-based registers) | | Adolescent (18 to 76 years old) | | Cross-sectional study | | Male 2407 (47.5%) & female 2174 (52.5%) | | Non-fatal suicidal behaviour: deliberate self-harm | | Multivariate | | Illicit drugs was associated with DSH, while controlling for history of sexual abuse, alcohol use, and female gender (aOR=2·06, 95% CI 1·05-4·04, p<0·035). | | Positive | | - | |
|  |  |  |  |  |  |  |  |  |  |  |  | Suicidal ideation | | Multivariate | | Illicit drugs was associated with suicidal plans, while controlling for history of sexual abuse, and female gender (aOR=2·62, 95% CI 1·05-6·53, p<0·038). | | Positive | |  |  |
|  |  |  |  |  |  |  |  |  |  |  |  | Suicidal ideation | | Multivariate | | Illicit drugs was associated with suicidal ideation, while controlling for history of sexual abuse, and female gender (aOR=4·04, 95% CI 2·14-7·66, p<0·001). | | Positive | |  |  |
|  |  | |  |  | |  | |  | |  | |  | |  | |  | |  | |  | |
| **Unspecified substance** | Use | | Diehl & Laranjeira, 2009 [44] | Brazil  Hospital based | | Adult (18 to 41 years old) | | Cohort study | | Male 22 (27.5%) & female 58 (72.5 %) | | Non-fatal suicidal behaviour: Suicide attempt | | Bivariate | | Drug use was not associated with the method of suicide attempt (p=0·205).  No other statistical results provided. | | Null | | - | |
|  |  | |  |  | |  | |  | |  | |  | |  | |  | |  | |  | |
| **Unspecified substance** | Use | | Ekramzadeh et al, 2012 [105] | Iran  Hospital based | | Adults (Mean = 70.5; SD = 7.5) | | Cross-sectional study | | Male 349 (61.2%) & female 221 (38.8%) | | Suicidal ideation | | Bivariate | | History of substance use was associated with harmful behaviours (β=0·087, t=2·25, p<0·05). | | Positive | | ++ | |
|  |  |  |  |  |  |  |  |  |  |  |  | Suicidal ideation | | Bivariate | | History of substance use was not associated with suicidal ideation. No statistical results provided. | | Null | |  |  |
|  |  | |  |  | |  | |  | |  | |  | |  | |  | |  | |  | |
| **Unspecified substance** | Use | | Kalyoncu et al, 2007 [106] | Turkey  Clinic-based (e.g. mobile clinic, free clinic) | | Adults (18 to 24 years old) | | Case-control study | | Male 78 (72.2%) & female 30 (27.8%) | | Non-fatal suicidal behaviour: Suicide attempt | | Bivariate | | Both the male (mean=6·32, SD= 1·15) and the female (mean=6.08, SD=0·66) suicide attempters had significantly higher drug use scores on the ASI measure than did the male (mean=4·58, SD=1·26) and the female (mean=4·33, SD=1·18) nonattempters (t=6·02, p<0·001; t=4·62, p<0·001, respectively). | | Positive | | ++ | |
|  |  |  |  |  |  |  |  |  |  |  |  | Non-fatal suicidal behaviour: Suicide attempt | | Bivariate | | Both the male (mean=2·82, SD=0·71) and the female (mean=3·25, SD=1·13) suicide attempters had significantly higher drug use scores on the ASI measure than did the male (mean=1·84, SD=0·73) and the female (mean=1·61, SD=0·69) nonattempters (t=6·31, p<0·001; t= 4·90, p<0·001, respectively). | | Positive | |  |  |
|  |  | |  |  | |  | |  | |  | |  | |  | |  | |  | |  | |
| **Unspecified substance** | Use | | Mahfoud et al, 2011 [39] | Lebanon  School based | | Adolescents (11 to 16 years old) | | Cross-sectional study | | Male 2333 (47.7%) & female 2776 (52.3%) | | Suicidal ideation | | Multivariate | | Using drugs was associated with suicidal ideation (aOR=2·07, 95% CI 1·33-3·20, p<0·05), while controlling for age, gender, and type of school. | | Positive | | + | |
|  |  |  |  |  |  |  |  |  |  |  |  | Suicidal ideation | | Bivariate | | Using drugs was associated with suicidal ideation (OR=5·42, 95% CI 3·83-7·67, p<0·05). | | Positive | |  |  |
|  |  | |  |  | |  | |  | |  | |  | |  | |  | |  | |  | |
| **Unspecified substance** | Use | | Miletic et al, 2015 [107] | Serbia  Other | | Adults (18 to 34 years old) | | Cross-sectional study | | Male 500 (38.6%) & female 794 (61.3 %) | | Non-fatal suicidal behaviour suicidal behaviour: suicide attempt | | Bivariate | | Drug use (p=0·007) was a signiﬁcant predictor of future suicide attempts. No other statistical results provided. | | Positive | | ++ | |
|  |  | |  |  | |  | |  | |  | |  | |  | |  | |  | |  | |
| **Unspecified substance** | Use | | Miller et al, 2011 [56] | Mexico  Community based | | Adolescents (12 to 17 years old) | | Cross-sectional study | | N/A | | Suicidal ideation | | Bivariate | | Lifetime use of illicit drugs was associated with suicidal ideation (OR=3·77, 95% CI 2·74-5·19, p<0·01). | | Positive | | ++ | |
|  |  |  |  |  |  |  |  |  |  |  |  | Suicidal ideation | | Bivariate | | Lifetime use of illicit drugs was associated with suicide plan (OR=6·29, 95% CI 3·58-11·2, p<0·01). | | Positive | |  |  |
|  |  |  |  |  |  |  |  |  |  |  |  | Non-fatal suicidal behaviour: Suicide attempt | | Bivariate | | Lifetime use of illicit drugs was associated with suicide attempt (OR=5·11, 95% CI 2·68-9·71, p<0·01). | | Positive | |  |  |
|  |  |  |  |  |  |  |  |  |  |  |  | Suicidal ideation | | Multivariate | | Lifetime use of illicit drugs was not associated with suicidal ideation (aOR=0·43, 95% CI 0·13-1·45, p>0·05), while controlling for psychiatric disorder or alcohol use/disorder or tobacco use/disorder. | | Null | |  |  |
|  |  |  |  |  |  |  |  |  |  |  |  | Suicidal ideation | | Multivariate | | Lifetime use of illicit drugs was not associated with suicide plan (OR=0·50, 95% CI 0·11-2·33, p>0·05), while controlling for psychiatric disorder or alcohol use/disorder or tobacco use/disorder. | | Null | |  |  |
|  |  |  |  |  |  |  |  |  |  |  |  | Non-fatal suicidal behaviour: Suicide attempt | | Multivariate | | Lifetime use of illicit drugs was not associated with suicide attempt (OR=0·47, 95% CI 0·12–1·89, p>0·05), while controlling for psychiatric disorder or alcohol use/disorder or tobacco use/disorder. | | Null | |  |  |
|  |  | |  |  | |  | |  | |  | |  | |  | |  | |  | |  | |
| **Unspecified substance** | Use | | Page, Dennis, et al, 2011 [55] | Philippines;  China;  Namibia  School based | | Adolescents (11 to 16 years old) | | Cross-sectional study | | Philippines: male 3094 (43.2%) & female 4188 (56.8%).  China: male 4356 (51.2%) & female 4537 (48.8%).  Namibia: male 2931 (45.2%) & female 3352 (54.8%). | | Suicidal ideation | | Bivariate | | Lifetime drug use was associated with suicide plan across gender in Philippine and Namibia:  Philippine boys % (n) lifetime drug users=24·8 (96); %(n) nonusers= 19·0 (475); (OR=1·30 95% CI 1·00-1·70, p<0·05)  Philippine girls % (n) lifetime drug users=37·9 (55); %(n) nonusers= 17·9 (701); (OR=2·55, 95% CI 1·79-3·65, p<0·05);  Namibia boys % (n) lifetime drug users=46·0 (323); %(n) nonusers= 26·8 (535); (OR=2·12, 95% CI 1·76-2·54, p<0·05);  Namibia girls % (n) lifetime drug users=49·6 (348); %(n) nonusers= 26·4 (641); (OR=2·47, 95% CI 2·06-2·97, p<0·05). | | Positive | | ++ | |
|  |  |  |  |  |  |  |  |  |  |  |  | Suicidal ideation | | Bivariate | | Lifetime drug use was not consistently associated with suicide plan across both genders in China:  China boys % (n) lifetime drug users=16·3 (15); %(n) nonusers= 6·5 (273); (OR=2·82, 95% CI 1·59-5·00, p>0·05);  China girls % (n) lifetime drug users=16·7 (8); %(n) nonusers= 9·4 (414); (OR=2·04, 95% CI 0·94-4·43, p>0·05). | | Unclear | |  |  |
|  |  | |  |  | |  | |  | |  | |  | |  | |  | |  | |  | |
| **Unspecified substance** | Use | | Page et al, 2011 [36] | China; Philippines  School based | | Adolescent (11 to 17 years old) | | Cross-sectional study | | China: male 4356 (51.2%) & female 4537 (48.8%).  Philippines: male 3094 (43.2%) & female 4188 (56.8%). | | Suicidal ideation | | Multivariate | | Among Chinese students, ever used drugs was significantly associated with suicide ideation (OR=1·36, 95% CI 1·27-1·46, p<0·001). | | Positive | | ++ | |
|  |  |  |  |  |  |  |  |  |  |  |  | Suicidal ideation | | Multivariate | | Among Chinese students, ever used drugs was associated with making a suicide plan (OR=1·64, 95% CI 1·05-1·13, p<0·001). | | Positive | |  |  |
|  |  |  |  |  |  |  |  |  |  |  |  | Suicidal ideation | | Multivariate | | Among Philippine students, ever used drugs was associated with suicide ideation (OR=1·71, 95% CI 1·48-1·96, p<0·001). | | Positive | |  |  |
|  |  |  |  |  |  |  |  |  |  |  |  | Suicidal ideation | | Multivariate | | Among Philippine students, ever used drugs was associated with making a suicide plan (OR=1·31, 95% CI 1·28-1·34, p<0·001). | | Positive | |  |  |
|  |  | |  |  | |  | |  | |  | |  | |  | |  | |  | |  | |
| **Unspecified substance** | Use | | Peltzer, 2009 [67] | Kenya; Namibia; Swaziland;  Uganda; Zambia; Zimbabwe  School based | | Adolescents (13 to 15 years old) | | Cross-sectional study | | N/A | | Suicidal ideation | | Multivariate | | Illicit drug use was not associated with suicidal ideation (aOR=0·95, 95% CI 0·93-0·96, p>0·05). | | Null | | + | |
|  |  |  |  |  |  |  |  |  |  |  |  | Suicidal ideation | | Multivariate | | Predict illicit drug use:  Suicide plan (aOR=1·53, 95% CI 1·51–1·58, p>0·05). | | Positive | |  |  |
|  |  | |  |  | |  | |  | |  | |  | |  | |  | |  | |  | |
| **Unspecified substance** | Use | | Peltzer & Pengpid, 2015 [50] | Oceania (Kiribati,  Samoa, Solomon Islands, and Vanuatu)  School based | | Adolescents (13 to 16 years old) | | Cross-sectional study | | Male 2846 (43.5%) & female 3534 (54%) | | Suicidal ideation | | Bivariate | | Drug use (illicit) initiation  was associated with suicidal ideation:  **Among the total sample**  Non-initiators 1.00  <12 years (OR=3·26, 95% CI 2·57-4·12, p<0·001);  ≥12 years (OR=2·62, 95% CI 2·09-3·27, p<0·001).  **Among boys**  Non-initiators 1.00  <12 years (OR=3·31, 95% CI 2·5-4·27, p<0·001);  ≥12 years (OR=2·29, 95% CI 1·6-3·27, p<0·001).  AND  **Among girls**  Non-initiators 1·00  <12 years (OR=3·22, 95% CI 2·14-4·85, p<0·001);  ≥12 years (OR=2·98, 95% CI 2·21-4·02, p<0·001). | | Positive | | ++ | |
|  |  |  |  |  |  |  |  |  |  |  |  | Non-fatal suicidal behaviour: S uicide attempt | | Bivariate | | Drug use (illicit) initiation  was associated with suicide attempt:  **Among the total sample**  Non-initiators 1.00  <12 years (OR=6·66, 95% CI 4·25-10·4, p<0·001);  ≥12 years (OR=3·89, 95% CI 2·86-5·28, p<0·001).  **Among boys**  Non-initiators 1.00  <12 years (OR=5·74, 95% CI 3·26-10·1, p<0·001);  ≥12 years (OR=3·56, 95% CI 2·45-5·18, p<0·001).  AND  **Among girls**  Non-initiators 1.00  <12 years (OR=7·30, 95% CI 4·43-12·0, p<0·001);  ≥12 years (OR=4·46, 95% CI 3·07-6·49, p<0·001). | | Positive | |  |  |
|  |  |  |  |  |  |  |  |  |  |  |  | Suicidal ideation | | Multivariate | | Drug use (illicit) initiation  was associated with suicidal ideation:  **Among the total sample**  Non-initiators 1.00  <12 years (aOR=2·21, 95% CI 1·48-3·30, p<0·001;  ≥12 years (aOR=1·88, 95% CI 1·34-2·63, p<0·001).  **Among boys**  Non-initiators 1·00  <12 years (aOR=1·04, 95% CI 0·56-1·94, p>0·05);  ≥12 years (aOR=1·08, 95% CI 0·57-2·07, p>0·05).  AND  **Among girls**  Non-initiators 1.00  <12 years (aOR=1·90, 95% CI 1·09-3·70, p<0·05);  ≥12 years (aOR=1·82, 95% CI 1·04-3·18, p<0·05).  (Adjusted for age, psychological distress and current  alcohol use) | | Positive | |  |  |
|  |  |  |  |  |  |  |  |  |  |  |  | Non-fatal suicidal behaviour: Suicide attempt | | Multivariate | | Drug use (illicit) initiation  was associated with suicide attempt:  **Among the total sample**  Non-initiators 1·00  <12 years (aOR=2·57, 95% CI 1·47-4·48, p<0·001);  ≥12 years (aOR=1·94, 95% CI 1·29-2·94, p<0·01).  **Among boys**  Non-initiators 1.00  <12 years (aOR=2·39, 95% CI 1·21-4·74, p<0·05);  ≥12 years (aOR=2·07, 95% CI 1·37-3·13, p<0·01).  AND  **Among girls**  Non-initiators 1·00  <12 years (aOR=2·87, 95% CI 1·63-5·07, p<0·001);  ≥12 years (aOR=1·84, 95% CI 1·05-3·22, p<0·01).  (Adjusted for age, psychological distress and current  alcohol use) | | Positive | |  |  |
|  |  |  |  |  |  |  |  |  |  |  |  | Suicidal ideation | | Bivariate | | **Among the total sample**  Substance use initiation with one, two and three substances at younger than 12 years old was associated with suicidal ideation among the total sample and girls but not boys:  One substance (OR=1·78, 95% CI 1·2-2·62, p<0·01);  Two substances (OR=4, 95% CI 2·9-5·39, p<0·001);  Three substances (OR=3·46, 95% CI 2·2-5·31, p<0·001).  **Among boys**  One substance (OR=1·41, 95% CI 0·4-2·19, p>0·05);  Two substances (OR=3·17, 95% CI 2·1-4·72, p<0·001);  Three substances (OR=3·03, 95% CI 1·7-5·28, p<0·001).  AND  **Among girls**  One substance (OR=2·31, 95% CI 1·23-4·33, p<0·01);  Two substances (OR=6·87, 95% CI 3·99-11·8, p<0·001);  Three substances (OR=4·98, 95% CI 2·38-10·4, p<0·001). | | Unclear | |  | |
|  |  |  |  |  |  |  |  |  |  |  |  | Non-fatal suicidal behaviour: Suicide attempt | | Bivariate | | Substance use initiation with one, two, or three substances at younger than <12 years old  was not consistently associated with suicide attempt:  **Among the total sample**  One substance (OR=2·29, 95% CI 1·58-3·32, p<0·001);  Two substances (OR=9·33, 95% CI 5·69-15·3, p<0·001);  Three substances (OR=12·4, 95% CI 7·24-21·4, p<0·001).  **Among boys**  Not  One substance (OR=1·73, 95% CI 0·97-3·11, p>0·05);  But yes  Two substances (OR=7·25, 95% CI 4·01-13·1, p<0·001);  Three substances (OR=10·9, 95% CI 5·96-19·8, p<0·001).  AND  **Among girls**  One substance (OR=2·84, 95% CI 1·72-4·69, p<0·001);  Two substances (OR=13·5, 95% CI 7·16-21·4, p<0·001);  Three substances (OR=32·9, 95% CI 14·1-76·9, p<0·001). | | Unclear | |  | |
|  |  |  |  |  |  |  |  |  |  |  |  | Suicidal ideation | | Multivariate | | Substance use initiation with one, two, or three substances at younger than <12 years old  was not consistently associated with suicidal ideation, while adjusting for age, psychological distress, current smoking, alcohol, and cannabis use:  **Among the total sample**  One substance (aOR=1·49, 95% CI 1·01–2·16, p<0·05);  Two substances (OaR=2·11, 95% CI 1·38-3·24, p<0.001);  Three substances (aOR=1·70, 95% CI 0·92-3·14, p>0·05).  **Among boys**  One substance (aOR=1·41, 95% CI 0·92-2·15, p>0·05);  Two substances (aOR=1·94, 95% CI 1·15-3·28, p<0·05);  Three substances (aOR=1·93, 95% CI 0·86-4·35, p>0·05).  AND  **Among girls**  One substance (aOR=1·59, 95% CI 0·79-3·17, p<0·05);  Two substances (aOR=3·16, 95% CI 1·46-6·83, p<0·01);  Three substances (aOR=1·18, 95% CI 0·52-2·66, p>0·05).  (Adjusted for age, psychological distress and current  alcohol use) | | Unclear | |  | |
|  |  |  |  |  |  |  |  |  |  |  |  | Non-fatal suicidal behaviour: Suicide attempt | | Multivariate | | Substance use initiation with one, two, or three substances at younger than <12 years old  was not consistently associated with suicide attempt, while adjusting for age, psychological distress, current smoking, alcohol, and cannabis use:  **Among the total sample**  One substance (aOR=1·39, 95% CI 0·92-2·12, p>0·05);  Two substances (aOR=2·86, 95% CI 1·79-4·57, p<0·001);  Three substances (aOR=3·46, 95% CI 2·01-5·95, p<0·001).  **Among boys**  One substance (aOR=1·11, 95% CI 0·65-1·88, p>0·05);  Two substances (aOR=2·98, 95% CI 1·65-5·37, p<0·001);  Three substances (aOR=3·31, 95% CI 1·63-6·73, p<0·001).  AND  **Among girls**  One substance (aOR=1·97, 95% CI 1·16-3·36, p<0·05);  Two substances (aOR=3·35, 95% CI 1·32-8·46, p<0·05);  Three substances (aOR=5·44, 95% CI 1·94-15·3, p<0·01).  (Adjusted for age, psychological distress and current  alcohol use) | | Unclear | |  | |
|  |  | |  |  | |  | |  | |  | |  | |  | |  | |  | |  | |
| **Unspecified substance** | Use | | Pumariega et al, 2014 [61] | Turkey  School based | | Adolescents (14 to 18 years old) | | Cross-sectional study | | Male 14 477 (46.6%) & female 16581 (53.4%) | | Suicidal ideation | | Bivariate | | Suicidal ideation was associated with any illicit drug use (except cannabis/marijuana) (OR=1·28, NO CI, p=0·000). | | Positive | | + | |
|  |  |  |  |  |  |  |  |  |  |  |  | Suicidal ideation | | Bivariate | | Suicidal ideation was associated with polysubstance use (any 2 of 9 illicit substances other than cannabis/marijuana) (OR=1·61, NO CI, p=0·000). | | Positive | |  |  |
|  |  | |  |  | |  | |  | |  | |  | |  | |  | |  | |  | |
| **Unspecified substance** | Use | | Randall et al, 2014 [83] | Republic of Benin, West Africa  School based | | Adolescents (11 to 16 years old) | | Cross-sectional study | | Male 1798 (67.1%) & female 882 (32.9%) | | Suicidal ideation | | Bivariate | | Among Benin adolescents who reported substance use, 2·8% (51) reported no suicidal ideation, 7·8% (9) reported ideation only, 9% (36) reported ideation with a plan, p<0·0001. | | Positive | | ++ | |
|  |  |  |  |  |  |  |  |  |  |  |  | Non-fatal suicidal behaviour: Suicide attempt | | Bivariate | | Substance use was associated with number of suicide attempts p<0·0001; no attempt=1·9% (35), one attempt =7.6%(24), 2 or more attempts =13·5%(38). | | Positive | |  |  |
|  |  |  |  |  |  |  |  |  |  |  |  | Suicidal ideation | | Multivariate | | Substance use was associated with suicidal ideation only (OR=3·06, 95% CI 1·12–8·34, p=0·031), while controlling for age, psycho-social symptoms, and socio-environmental factors. | | Positive | |  |  |
|  |  |  |  |  |  |  |  |  |  |  |  | Suicidal ideation | | Multivariate | | Substance use was associated with suicidal ideation with a plan (OR=1·69, 95% CI 1·05–2·72, p=0·032), while controlling for age, psycho-social symptoms, and socio-environmental factors. | | Positive | |  |  |
|  |  |  |  |  |  |  |  |  |  |  |  | Non-fatal suicidal behaviour: Suicide attempt | | Multivariate | | Substance use was not associated with having one past suicide attempt (OR=2·70, 95% CI 0·93–7·85, p=0·066), while controlling for age, psycho-social symptoms, and socio-environmental factors. | | Null | |  |  |
|  |  |  |  |  |  |  |  |  |  |  |  | Non-fatal suicidal behaviour: Suicide attempt | | Multivariate | | Substance use was associated with having two or more suicide attempts (OR=5·73, 95% CI 2·99–11·0, p=0·001), while controlling for age, psycho-social symptoms, and socio-environmental factors. | | Positive | |  |  |
|  |  | |  |  | |  | |  | |  | |  | |  | |  | |  | |  | |
| **Unspecified substance** | Use | | Sharma et al, 2015 [58] | Peru  School based | | Adolescents (12 to 18 years old) | | Cross-sectional study | | Male 425 (46.4%) & female 491 (53.6%) | | Suicidal ideation | | Multivariate | | Illicit drug use was not associated with increased likelihood of suicidal ideation, while controlling for psychological (aOR=1·54, 95% CI 0·84-2·83, p>0·05), and behavioural factors. | | Null | | ++ | |
|  |  |  |  |  |  |  |  |  |  |  |  | Non-fatal suicidal behaviour: Suicide attempt | | Multivariate | | Illicit drug use was associated with increased likelihood of suicide attempts (aOR=2·91, 95% CI 1·51–5·61, p<0·05), while controlling for psychological, socio-environmental, and demographic factors. | | Positive | |  |  |
|  |  | |  |  | |  | |  | |  | |  | |  | |  | |  | |  | |
| **Unspecified substance** | Use | | Sitdhiraksa et al, 2014 [33] | Thailand  School based | | Both (12 to 21 years old) | | Cross-sectional study | | Male 1054 (40.2%) & female 1568 (59.8%) | | Non-fatal suicidal behaviour: Thoughts of self-harm + Suicide attempt | | Bivariate | | Illicit drug use within the last 12 months was associated with thoughts of harming yourself or attempted suicide (OR=2·08, 95% CI 1·61-2·69, p<0·01). | | Positive | | + | |
|  |  | |  |  | |  | |  | |  | |  | |  | |  | |  | |  | |
| **Unspecified substance** | Use | | Souza et al, 2010 [38] | Brazil  National/regional registers (e.g. not clinical-based registers) | | Adolescents (11 to 15 years old) | | Cross-sectional study | | Male 501 (48.2%) &  female 538 (51.8%) | | Suicidal ideation | | Multivariate | | Illicit drug use was associated with suicidal ideation (OR=2·89, 95% CI 1·33 to 6·28, p<0·05), while controlling for gender, age, socioeconomic status, level of education, grade retension, religious practice, sexual activity, tobacco use, getting drunk, conduct disorder and high CDI scores for depressive symptoms. | | Positive | | ++ | |
|  |  | |  |  | |  | |  | |  | |  | |  | |  | |  | |  | |
| **Unspecified substance** | Use | | Swahn et al, 2012 [32] | Uganda  Other | | Both (14 to 24 years old) | | Cross-sectional study | | Male 142 (31.1%) & female 315 (68.5%) | | Suicidal ideation | | Multivariate | | Any drug use was not associated with suicide ideation (aOR=1·96, 95% CI 0·90-4·28, p>0·05), while controlling for gender, school attendance, whether one or both parents were dead, parental physical abuse, parental neglect due to alcohol use, apprenticeship skills, any drug use, any drunkenness, any STD/HIV, any traded sex, any rape, sadness, lonely, expect to die early. | | Null | | ++ | |
|  |  | |  |  | |  | |  | |  | |  | |  | |  | |  | |  | |
| **Unspecified substance** | Use | | Zarrouq et al, 2015 [81] | Morocco  School based | | Adolescents (11 to 23 years old) | | Cross-sectional study | | Males 1602 (53 %) & females 1418 (47 %) | | Suicidal ideation  Non-fatal suicidal behaviour: Suicide attempts & behaviour | | Bivariate | | Being a psychoactive substance user was associated with death wish (OR=36·8, 95% CI 30·9–43·1, p<0·001), self-harm wish (OR=31·7, 95% CI 26.1–37·8, p<0.001), suicide ideation (OR=26·7, 95% CI 21·4–32·6, p<0.001), suicide plan (OR=14·9, 95% CI 10·8–19·9, p<0·001), suicide attempt in the past month (OR=16·5, 95% CI 12·1–21·6, p<0.001), and lifetime suicide attempts (OR=19·6, 95% CI 14·9–25·1, p<0·001). | | Positive | | ++ | |
|  |  | |  |  | |  | |  | |  | |  | |  | |  | |  | |  | |
| **Unspecified substance** | Misuse | | Cluver et al, 2015 [114] | South Africa  Community based | | Adolescents (10 to 18 years old) | | Cohort study | | Male 1475 (44%) & female 1926 (56%) | | Non-fatal suicidal behaviour: suicide attempt, suicide plan | | Mediation regression (multivariate) | | There were no associations between drug/alcohol misuse and suicide behaviors (r=0·08, p>0·05). | | Null | | ++ | |
|  |  | |  |  | |  | |  | |  | |  | |  | |  | |  | |  | |
| **Unspecified substance** | Abuse | | Ahmad et al, 2014 [45] | Malaysia  School-based | | Adolescents (12 to 17 years old) | | Cross-sectional study | | Male (49.6%) & female (50.4 %) | | Suicidal ideation | | Multivariate | | Current drug abuse was associated with suicidal ideation (aOR=1·12, 95% CI 0·72-1·74, p=0·61), while controlling for gender, ethnicity, parental marital status, current smoking, current drug use, were bullied, physically abused at home, verbally abused at home, stress, anxiety, depression, have close friend, supportive peers, parental supervision, parental connectedness, parental bonding, parental respect for privacy. | | Null | | ++ | |
|  |  | |  |  | |  | |  | |  | |  | |  | |  | |  | |  | |
| **Unspecified substance** | Abuse | | Altamura et al, 2007 [84] | Hospital-based | | Adults [South African Attempters = 31.2 (9.0); South African non-attempters = 39.3 (17.7)] | | Cross-sectional study | | Attempters: male 20 (64.5%) & female 11 (35.5%)  Non-attempters: male 3 (50%) & female 3 (50%) | | Non-fatal suicidal behaviour: Suicide attempt | | Bivariate | | The presence of lifetime comorbid substance abuse was not associated with a suicide attempt during the course of their illness among schizophrenia spectrum disorder patients at high risk for suicide (x^2^=0·676, df=1, p>0·4). | | Null | | - | |
|  |  | |  |  | |  | |  | |  | |  | |  | |  | |  | |  | |
| **Unspecified substance** | Abuse | | Alvarado-Esquivel et al, 2014 [43] | Mexico  Hospital-based | | Adults [sample: 18-61 years old (mean 34.14+/-  10.24 years old). Control:  18-69 years old (mean 38.23+/-11.76 years old). | | Case-control study | | Male 85 (30.8%) & female 191 (69.2%) | | Non-fatal suicidal behaviour: Suicide attempt | | Multivariate | | Drug abuse was not associated with suicide attempts (OR=1·10, 95% CI 0·46-2·64, p=0·81). | | Null | | + | |
|  |  | |  |  | |  | |  | |  | |  | |  | |  | |  | |  | |
| **Unspecified substance** | Abuse | | Govender et al, 2013 [115] | South Africa  School based | | Adolescents (13 to 17 years old) | | Cross-sectional study | | Male 112 (46.9%) & female 127 (53.1%) | | Suicidal ideation | | Bivariate | | Suicide ideation was positively correlated with substance abuse (r=·36, p<·01). | | Positive | | + | |
|  |  | |  |  | |  | |  | |  | |  | |  | |  | |  | |  | |
| **Unspecified substance** | Abuse | | Khasakhala et al, 2013 [65] | Kenya  Hospital based | | Both (13 to 25 years old) | | Cross-sectional study | | Male 447 (48.7%) & female 471 (51.3%) | | Non-fatal suicidal behaviour: Suicidal behaviour | | Multivariate | | Any drug abuse was associated with suicidal behaviour (aOR=5·23, 95% CI 1·88–13·9, p<0·001). | | Positive | | ++ | |
|  |  |  |  |  |  |  |  |  |  |  |  |  |  | Multivariate | | Any drug abuse was associated with suicidal behaviour (aOR=6·66, 95% CI 2·81–15·8, p<0.001), while controlling for other mental health status. | | Positive | |  |  |
|  |  | |  |  | |  | |  | |  | |  | |  | |  | |  | |  | |
| **Unspecified substance** | Abuse | | Maraš et al, 2013[111] | Serbia  Hospital based | | Adolescents (15 to 18 years old) | | Case-control study | | Male 12 (19%) & female 20 (81%) | | Non-fatal suicidal behaviour: Suicide attempt | | Bivariate | | Suicide attempt was associated with substances abuse (x^2^=7·398, df=1, p<0·01). | | Positive | | - | |
|  |  | |  |  | |  | |  | |  | |  | |  | |  | |  | |  | |
| **Unspecified substance** | Abuse | | Menezes et al, 2012 [92] | Nepal  University students | | Adults (18 to 27 years old) | | Cross-sectional study | | Males 112 (54.4%) & females 94 (45.6%) | | Suicidal ideation | | Bivariate | | A greater proportion of suicidal ideation students reported substance abuse compared to those who did not report substance abuse (50% vs. 9·9%) but this was not a statistically significant difference (OR=9·1, 95% CI 1·22-68·2, p=0·032). | | Null | | ++ | |
|  |  | |  |  | |  | |  | |  | |  | |  | |  | |  | |  | |
| **Unspecified substance** | Abuse | | Monsef Kasmaee et al, 2015 [110] | Iran  Hospital based | | Both (12 to 80 years old) | | Cross-sectional study | | Male 49 (83%) & female 10 (17%) | | Fatal suicide | | Bivariate | | Substance abuse status was not significantly different between the outcome death (11·1% vs. 38%) and survive (88·9% vs. 62%), (p=0·12). | | Null | | + | |
|  |  | |  |  | |  | |  | |  | |  | |  | |  | |  | |  | |
| **Unspecified substance** | Abuse | | Myint et al, 2014 [41] | Thailand  Hospital based | | Both (10 to 79 years old) | | Cohort study | | Male 136 (91.3%) & 13 (8.7%) | | Fatal suicide | | Bivariate | | Suicide was not associated with substance abuse (x^2^=0·486, df=1, p=0·486). | | Null | | ++ | |
|  |  | |  |  | |  | |  | |  | |  | |  | |  | |  | |  | |
| **Unspecified substance** | Abuse | | Osama et al, 2014 [91] | Pakistan  Others | | Adults (18 to 29 years old) | | Cross-sectional study | | 135 Males 135 (41.2%) & female 193 (58.8%) | | Suicidal ideation | | Multivariate | | Indulging in substance abuse was associated with suicidal ideation (OR=28, 95% CI 3·703-220·1, p<0·001). | | Positive | | - | |
|  |  | |  |  | |  | |  | |  | |  | |  | |  | |  | |  | |
| **Unspecified substance** | Abuse | | Ruengorn et al, 2012 [51] | Thailand  Hospital based | | Both (13 to 60 years old) | | Case-control study | | Male 354 (32.2%) & female 746 (67.8%) | | Non-fatal suicidal behaviour: Suicide attempt | | Bivariate | | Any substance abuse was not associated with suicide attempt (OR=1·76, CI 0·92-3·36, p<0·001). | | Null | | - | |
|  |  | |  |  | |  | |  | |  | |  | |  | |  | |  | |  | |
| **Unspecified substance** | Abuse | | Sadr et al, 2013 [112] | Iran  Hospital | | Both (15 to 60 years old) | | Cross-sectional study | | Male 175 (43.8%) & female 225 (56.6%) | | Non-fatal suicidal behaviour: Suicide attempt | | Bivariate | | Substance abuse was associated with multiple attempts of suicide in men (OR=1·020, NO CI, p=0·033) and women (OR=1·012, NO CI, p=0·045). | | Positive | | + | |
|  |  | |  |  | |  | |  | |  | |  | |  | |  | |  | |  | |
| **Unspecified substance** | Abuse | | Seghatoleslam et al, 2012 [113] | Iran  Hospital based | | Both (15 to 60 years old) | | Cross-sectional study | | Female 100% | | Non-fatal suicidal behaviour: Suicide attempt | | Bivariate | | Substance abuse was associated with multiple suicide attempts (OR=1·020, NO CI, p=0·033). | | Positive | | - | |
|  |  | |  |  | |  | |  | |  | |  | |  | |  | |  | |  | |
| **Unspecified substance** | Abuse | | Shakeri et al, 2015 [123] | Iran  Hospital based | | Adults (Mean = 27.7; SD = 9.49) | | Cross-sectional study | | Female 100% | | Non-fatal suicidal behaviour: suicide attempt, past suicide attempts | | Bivariate | | History of drug abuse was not associated with repeated suicide attempts (p=0·290). | | Null | | ++ | |
|  |  | |  |  | |  | |  | |  | |  | |  | |  | |  | |  | |
| **Unspecified substance** | Addiction | | Lavania et al, 2012 [77] | India  Hospital based | | Adults (18 to 60 years old) | | Cross-sectional study | | Male 100% | | Non-fatal suicidal behaviour: Deliberate self-harm | | Bivariate | | Drugs, mean (SD): group I with DSH 6·83 (2·55); group II without DSH 3·13 (3·49). Those patients with DSH reported a significantly higher mean for drug addiction severity compared to patients without DSH (t=4·69, p<0·001). | | Positive | | - | |
|  |  | |  |  | |  | |  | |  | |  | |  | |  | |  | |  | |
| **Unspecified substance** | Addiction | | Neves et al, 2009 [94] | Brazil  Hospital based | | Adults (Mean = 38.1; SD = 12.2) | | Cohort study | | Male 68 (28.5%) & female 171 (58.4%) | | Non-fatal suicidal behaviour: Suicide attempts | | Bivariate | | Other drug addiction was associated with a history of suicide attempts (x^2^=10·1, df=1, p<0·02). | | Positive | | + | |
|  |  |  |  |  |  |  |  |  |  |  |  | Non-fatal suicidal behaviour: Suicide attempts | | Multivariate | | Other drug addiction was not associated with a history of suicide attempts in bipolar patients (OR=1·73, NO CI, p=0·243), while controlling for confounding factors. | | Null | |  |  |
|  |  |  |  |  |  |  |  |  |  |  |  | Non-fatal suicidal behaviour: Suicide attempts | | Multivariate | | Other drug addiction was not associated with a history of violent suicide attempts in bipolar patients (OR=1·26, NO CI, p=0·706), while controlling for confounding factors. | | Null | |  |  |
|  |  | |  |  | |  | |  | |  | |  | |  | |  | |  | |  | |
| **Unspecified substance** | Addiction | | Neves et al, 2010 [72] | Brazil  Clinic-based (e.g. mobile clinic, free clinic) | | Adult (Non suicide group Mean = 44.6; SD = 13, suicide group Mean = 38.8; SD = 12.1) | | Case-control study | | Male 62 (30.9%) & female 139 (69.1%) | | Non-fatal suicidal behaviour: Suicide attempts | | Bivariate | | Other drug addiction was associated with lifetime history of suicide attempt (23·3% vs. 8%), (x2=9·01, df=1, p=0·003). | | Positive | | - | |
|  |  |  |  |  |  |  |  |  |  |  |  | Non-fatal suicidal behaviour: Suicide attempts | | Bivariate | | Other drug addiction was not significantly associated with violent suicide attempt (27·3% vs. 20·8%), (x^2^=0·484, df=1, p=0·487). | | Null | |  |  |
|  |  | |  |  | |  | |  | |  | |  | |  | |  | |  | |  | |
| **Unspecified substance** | Abuse/dependence | | Miller et al, 2011 [56] | Mexico  Community based | | Adolescents (12 to 17 years old) | | Cross-sectional study | | N/A | | Suicidal ideation | | Bivariate | | Illicit drug abuse/dependence was associated with suicidal ideation (OR=6·24, 95% CI 2·73-14·3, p<0·01). | | Positive | | ++ | |
|  |  |  |  |  |  |  |  |  |  |  |  | Suicidal ideation | | Bivariate | | Illicit drug abuse/dependence was associated with suicide plan (OR=13·3, 95% CI 5·44-32·5, p<0·01). | | Positive | |  |  |
|  |  |  |  |  |  |  |  |  |  |  |  | Non-fatal suicidal behaviour: Suicide attempt | | Bivariate | | Illicit drug abuse/dependence was associated with suicide attempt (OR=6·79, 95% CI 2·15-21·5, p<0·01). | | Positive | |  |  |
|  |  |  |  |  |  |  |  |  |  |  |  | Suicidal ideation | | Multivariate | | Illicit drug abuse/dependence was not associated with suicidal ideation (OR=1·11, 95% CI 0·19-6·44, p>0·05), while controlling for psychiatric disorder or alcohol use/disorder or tobacco use/disorder. | | Null | |  |  |
|  |  |  |  |  |  |  |  |  |  |  |  | Suicidal ideation | | Multivariate | | Illicit drug abuse/dependence was associated with suicide plan (OR=3·32, 95% CI 1·05-10·5, p<0·05), while controlling for psychiatric disorder or alcohol use/disorder or tobacco use/disorder. | | Positive | |  |  |
|  |  |  |  |  |  |  |  |  |  |  |  | Non-fatal suicidal behaviour: Suicide attempt | | Multivariate | | Illicit drug abuse/dependence was not associated with suicide attempt (OR=0·94, 95% CI 0·15-5·70, p<0·01), while controlling for psychiatric disorder or alcohol use/disorder or tobacco use/disorder. | | Null | |  |  |
|  |  | |  |  | |  | |  | |  | |  | |  | |  | |  | |  | |
| **Unspecified substance** | Dependence | | Chan et al, 2011 [118] | Malaysia  Hospital-based | | Adult | | Cross-sectional study | | Male 23 (44%) & Female 42 (56%) | | Non-fatal suicidal behaviour: Suicide attempt | | Bivariate | | Other (amphetamine, opiates, sedatives) (8·7% vs. 7·7%, p>0·05) and any substance abuse or dependence (47·8% vs. 38·5%, p>0·05) was not associated with suicide attempt. | | Null | | ++ | |
|  |  | |  |  | |  | |  | |  | |  | |  | |  | |  | |  | |
| **Unspecified substance** | Dependence | | Diehl & Laranjeira, 2009 [44] | Brazil  Hospital based | | Adult (18 to 41 years old) | | Cohort study | | Male 22 (27.5%) & female 58 (72.5 %) | | Non-fatal suicidal behaviour: Suicide attempt | | Bivariate | | Substance dependence was not associated with suicide attempt (p=0·204).  No other statistical results provided. | | Null | | - | |
|  |  | |  |  | |  | |  | |  | |  | |  | |  | |  | |  | |
| **Unspecified substance** | Dependence | | Evren et al, 2012 [104] | Turkey  Hospital based | | N/A | | Cross-sectional study | | Male 100% | | Non-fatal suicidal behaviour: Self-mutilation | | Bivariate | | Drug dependence, when compared to alcohol-dependence, was associated with self-mutilative behaviour (x^2^=14·4, df=1, p<0·001). | | Positive | | ++ | |
|  |  |  |  |  |  |  |  |  |  |  |  | Non-fatal suicidal behaviour: Self-mutilation | | Bivariate | | Risk of self-mutilation was higher in drug-dependent inpatients, when compared to alcohol-dependent inpatients (OR=3·12, 95% CI 1·72–5·68, p<0·05). | | Positive | |  |  |
|  |  |  |  |  |  |  |  |  |  |  |  | Non-fatal suicidal behaviour: Self-mutilation | | Bivariate | | Risk of self-mutilation was higher among substance-dependent inpatients with prominent  borderline features (PBF) than those without PBF (OR=4·2, 95% CI 2·23-7·82). | | Positive | |  |  |
|  |  | |  |  | |  | |  | |  | |  | |  | |  | |  | |  | |
| **Unspecified substance** | Dependence | | Evren et al, 2012 [120] | Turkey  Hospital based | | N/A | | Cross-sectional study | | Male 100% | | Non-fatal suicidal behaviour: Self-mutilation | | Bivariate | | The rate of self-mutilation was higher among drug dependents than the alcohol dependents (75·0% vs. 49%), (x^2^=14·4, df=1, p<0·001),  and the risk was 3·12 (95% CI 1·72-5·68) times higher. | | Positive | | ++ | |
|  |  | |  |  | |  | |  | |  | |  | |  | |  | |  | |  | |
| **Unspecified substance** | Dependence | | Evren et al, 2006 [121] | Turkey  Hospital based | | Adults (18 years old and older) | | Cross-sectional study | | Male 100% | | Non-fatal suicidal behaviour: Self-mutilation | | Bivariate | | Self-mutilation behavior was more prevalent in drug dependents than alcohol dependents (53·6% vs. 12·5%) (x^2^=21·35, df=1, p<0·001), (OR=8·08, 95% CI=3·1–20·9, p<0·05). | | Positive | | ++ | |
|  |  | |  |  | |  | |  | |  | |  | |  | |  | |  | |  | |
| **Unspecified substance** | Dependence | | Uzun et al, 2009 [141] | Turkey  Hospital based | | Adult (Age Mean = 36.7; SD = 11.8 years old) | | Cohort study | | Male 195 (65%) & female 105 (35%) | | Non-fatal suicidal behaviour: Suicide attempt | | Bivariate | | Those who had attempted suicide did not have a significantly higher rate of comorbid substance abuse or dependence than that of those who had not (5·8% vs. 3·6%), (Fishers’ exact test, df=4, p=0·112). No other statistical results provided. | | Null | | + | |
|  |  | |  |  | |  | |  | |  | |  | |  | |  | |  | |  | |
| **Unspecified substance** | Disorder | | Chan et al, 2014 [96] | Malaysia  Clinic-based (e.g. mobile clinic, free clinic) | | Adults (18 to 76 years old) | | Cohort study | | Male 33 (44%) & female 42 (56%) | | Non-fatal suicidal behaviour: Suicide attempt | | Bivariate | | Any type of substance use disorder was associated with future suicide attempt (OR=6·95, % CI 1·45-24·9, p=0·01). | | Positive | | ++ | |
|  |  |  |  |  |  |  |  |  |  |  |  | Non-fatal suicidal behaviour: Suicide attempt | | Bivariate | | Any type of substance use disorder was associated with the transition from suicidal ideation to future suicide attempt (OR=4·13, 95% CI 0·96-17·7, p=0·06). | | Null | |  |  |
|  |  |  |  |  |  |  |  |  |  |  |  | Non-fatal suicidal behaviour: Suicide attempt | | Multivariate | | Any type of substance use disorder was associated with future suicide attempt (OR=7·82, 95% CI 1·50–40·8, p=0·02), while controlling for previous psychiatric hospitalization, and major personal injury or illness. | | Positive | |  |  |
|  |  | |  |  | |  | |  | |  | |  | |  | |  | |  | |  | |
| **Unspecified substance** | Disorder | | Coêlho et al, 2010 [143] | Brazil  Community based | | Adult (18 years old and older | | Cross-sectional study | | N/A | | Suicidal ideation | | Multivariate | | Substance use disorder was associated with thoughts of death (OR=2·5, 95% CI 1·5-4·3, p<0.0004), desire of death (OR=2·1, 95% CI 1·1-4·1, p<0·03), suicide thought (OR=2·7, 95% CI 1·4-5·3, p<0·0002). | | Positive | | ++ | |
|  |  |  |  |  |  |  |  |  |  |  |  | Non-fatal suicidal behaviour: suicide attempt | | Multivariate | | Substance use disorder was not associated with suicide attempt (OR=0·6, 95% CI 0·1-3·9, p=0·60). | | Null | |  |  |
|  |  |  |  |  |  |  |  |  |  |  |  | Suicidal ideation | | Multivariate | | Substance use disorder was associated with thoughts of death (aOR=2·8, 95% CI 1·4-5·6, p<0·003), while controlling for gender, marital status, age group, education level, major depressive disorder, controlled by all demographic variables and the interaction with gender, and considering alcohol use disorder as effect modification, and two-way interaction of major depressive episode and substance use disorder, and gender with other socio-demographic variables, and major depressive episode and substance use disorder. Three-way interaction of major depressive episode, substance use disorder, and gender could not be calculated. | | Positive | |  |  |
|  |  | |  |  | |  | |  | |  | |  | |  | |  | |  | |  | |
| **Unspecified substance** | Disorder | | Kittirattanapaiboon et al, 2014 [119] | Thailand  Community based | | Both (15 to 59 years old) | | Cross-sectional study | | N/A | | Non-fatal suicidal behaviour: Suicidal attempts | | Multivariate | | Illicit drug use was associated with risk of suicide (aOR=2·09, 95% CI 1·55-2·81, p<0·001). | | Positive | | + | |
|  |  | |  |  | |  | |  | |  | |  | |  | |  | |  | |  | |
| **Unspecified substance** | Disorder | | Nock et al, 2009 [78] | Brazil;  Bulgaria;  Colombia;  India;  Lebanon;  Mexico;  Nigeria;  China (Beijing & Shanghai & Shenzhen);  Romania;  South Africa;  Ukraine  Community based | | Adult (18 years old and older) | | Cross-sectional study | | N/A | | Non-fatal suicidal behaviour: Suicide attempt | | Bivariate | | Any substance use disorder was associated with lifetime suicide attempt (OR=4·8, 95% CI 4·0–5·8, p<0·05). | | Positive | | + | |
|  |  |  |  |  |  |  |  |  |  |  |  | Non-fatal suicidal behaviour: Suicide attempt | | Bivariate | | Any substance use disorder was associated with subsequent suicide attempt (OR=5·4, 95% CI 4·3–6·8, p<0·05). | | Positive | |  |  |
|  |  |  |  |  |  |  |  |  |  |  |  | Non-fatal suicidal behaviour: Suicide attempt | | Multivariate | | Any substance use disorder was not associated with lifetime suicide attempt, while controlling for age, age-squared, age cohorts, sex, and personyear. No statistical results provided. | | Null | |  |  |
|  |  |  |  |  |  |  |  |  |  |  |  | Non-fatal suicidal behaviour: Suicide attempt | | Multivariate | | Any substance use disorder was not associated with lifetime suicide attempt p>0·05, while controlling for age, age-squared, age cohorts, sex, and person-year. No statistical results provided. | | Null | |  |  |
|  |  |  |  |  |  |  |  |  |  |  |  | Non-fatal suicidal behaviour: Suicide attempt | | Multivariate | | Substance use disorder was not associated with suicide attempt, while controlling for age, age-squared, age cohorts, sex, and personyear. No statistical results provided. | | Null | |  |  |
|  |  |  |  |  |  |  |  |  |  |  |  | Non-fatal suicidal behaviour: Suicide attempt | | Multivariate | | Substance use disorder was not associated with subsequent suicide attempt, while controlling for age, age-squared, age cohorts, sex, and person-year. No statistical results provided. | | Null | |  |  |
|  |  | |  |  | |  | |  | |  | |  | |  | |  | |  | |  | |
| **Unspecified substance** | Disorder | | Tong & Phillips, 2010 [108] | China  National/regional registers (e.g. not clinical-based registers) | | Both | | Case-control study | | Male 986 (61.8%) & female 610 (38.2%) | | Fatal suicide | | Multivariate | | Substance use disorders was associated with suicide risk (OR=2·71, 95% CI 1·81-4·07, p<0·05), while controlling for gender, age, urban versus rural residence and five diagnostic groups (mood disorders, anxiety disorders, psychotic disorders, substance use disorders, organic mental disorders, other mental disorders). | | Positive | | ++ | |
|  |  |  |  |  |  |  |  |  |  |  |  | Fatal suicide | | Multivariate | | Suicide risk associated with substance misuse among men  males (OR=1·79, 95% CI 1·22-2·62, p<0·05) and women (OR=1·77, 95% CI 0·49-6·31, p<0·05), while adjusting for age, and urban versus rural residence, substance use disorders. | | Positive | |  |  |
|  |  | |  |  | |  | |  | |  | |  | |  | |  | |  | |  | |
| **Unspecified substance** | Disorder | | Torres et al, 2011 [126] | Brazil  Clinic-based (e.g. mobile clinic, free clinic) | | N/A | | Cross-sectional study | | Male 254 (43.6%) & female 328 (56.4%) | | Suicidal ideation: suicide thought | | Multivariate | | Substance use disorder was associated with current suicidal thought (aOR=3·33, 95% CI 1·44-7·68, p=0·005), lifetime suicidal thoughts (aOR=2·56, 95% CI 1·21-5·45, p=0·014), while controlling for other psychiatric disorders. | | Positive | | ++ | |
|  |  |  |  |  |  |  |  |  |  |  |  | Non-fatal suicidal behaviour: suicide plan | | Multivariate | | Substance use disorder was associated with lifetime suicidal plans (aOR=2·06, 95% CI 1·01-4·23, p=0·048), while controlling for other psychiatric disorders. | | Positive | |  |  |
|  |  | |  |  | |  | |  | |  | |  | |  | |  | |  | |  | |
| **Unspecified substance** | Disorder | | Uwakwe & Gureje, 2011 [117] | Nigeria  Community based | | Adults (18 years old and older) | | Cross-sectional study | | N/A | | Non-fatal suicidal behaviour: Suicide behaviour | | Bivariate | | Any substance use disorder was associated with suicide attempt (OR=6·3, 95% CI 1·9-20·5, p<0·05). | | Positive | | ++ | |
|  |  |  |  |  |  |  |  |  |  |  |  | Non-fatal suicidal behaviour: Suicide behaviour | | Multivariate | | Substance use disorder was not associated with suicide attempt (OR=2·4, 95% CI 0·4-14·4, p=0·11), while controlling for any anxiety disorder, any mood disorder, any impulse disorder, and any disorder. | | Null | |  |  |
|  |  |  |  |  |  |  |  |  |  |  |  | Non-fatal suicidal behaviour: Suicide behaviour | | Multivariate | | Substance use disorder was not associated with suicide attempt (OR=2·4, 95% CI 0·4-14·4, p=0·11), while controlling for any anxiety disorder, any mood disorder, any impulse disorder, and any disorder, age, age-squared, sex, cohorts, and int categories. | | Null | |  |  |
|  |  |  |  |  |  |  |  |  |  |  |  | Suicidal ideation | | Multivariate | | Any substance use was associated with suicidal ideation (OR=3·3, 95% CI 1·0-10·4, p<0·05), while controlling for any anxiety disorder, any mood disorder, any impulse disorder, and number of other disorders. | | Positive | |  |  |
|  |  |  |  |  |  |  |  |  |  |  |  | Non-fatal suicidal behaviour: Suicide behaviour | | Multivariate | | Any substance use was not associated with suicide attempt (OR=3·0, 95% CI 0·6–15·7, p=0·19), attempt among ideators with a lifetime plan (OR=0·6, 95% CI 0·1–2·9, p=0·53), attempt among ideators without a lifetime plan (no statistical results provided), and plan among ideators (OR=0·9, 95% CI 0·2–3·3, p=0·83). | | Null | |  |  |
|  |  | |  |  | |  | |  | |  | |  | |  | |  | |  | |  | |
| **Unspecified substance** | Disorder | | Wei et al, 2013 [34] | China  Hospital based | | Both (15 to 60 years old) | | Cohort study | | Male 53 (22.2%) & female 186 (77.8%) | | Non-fatal suicidal behaviour: Suicide attempt | | Bivariate | | A greater proportion of substance-related disorder patients reported impulsive suicide attempt when compared to nonimpulsive suicide attempt (5·6% vs. 0%), (x^2^=5·47, df=1, p=0·019). | | Positive | | ++ | |
|  |  | |  |  | |  | |  | |  | |  | |  | |  | |  | |  | |
| **Unspecified substance** | Disorder | | Zhang, Xiao, & Zhou, 2010 [109] | China  Community | | Both (15 to 34 years old) | | Case-control study | | Male 214 (51.5%) & female 178 (48.5%) | | Fatal suicide | | Bivariate | | A greater proportion of substance use disorder individuals were suicide victims when compared to the control group (6·4% vs. 0·9%), no other statistical results provided). | | Positive | | ++ | |
|  |  | |  |  | |  | |  | |  | |  | |  | |  | |  | |  | |
| **Unspecified substance** | Multiple: abuse & dependence | | Borges et al, 2010 [124] | Mexico  National/ regional registers (e.g. not clinical-based registers) | | Adult (18 to 65 years old) | | Cross-sectional study | | N/A | | Non-fatal suicidal behaviour: Suicide attempt | | Multivariate | | Lifetime suicide attempt was not associated with drug abuse or dependence (OR=2·6, 95% CI 0·6-11·1, p>0·05), while controlling for age, age-squared, sex, cohorts, and initial categories. | | Null | | ++ | |
|  |  |  |  |  |  |  |  |  |  |  |  | Suicidal ideation | | Multivariate | | Lifetime suicidal ideation was associated with drug abuse or dependence (OR=4·8, 95% CI 1·8-12·7, p<0·05), while controlling for age, age-squared, sex, cohorts, and initial categories. | | Positive | |  |  |
|  |  |  |  |  |  |  |  |  |  |  |  | Suicidal ideation | | Multivariate | | Among the subgroup of ideators:  drug abuse or dependence was not associated with a plan among ideators (OR=0·3, 95% CI 0·1-1·1, p>0·05). | | Null | |  |  |
|  |  |  |  |  |  |  |  |  |  |  |  | Non-fatal suicidal behaviour: Suicide attempt | | Multivariate | | Drug abuse or dependence was not associated with a planned attempt (OR=1·2, 95% CI 0·1-9·4, p>0·05). | | Null | |  |  |
|  |  |  |  |  |  |  |  |  |  |  |  | Non-fatal suicidal behaviour: Suicide attempt | | Multivariate | | Drug abuse or dependence was not associated with an unplanned attempt (OR=0·7, 95% CI 0·1-3·8, p>0·05). | | Null | |  |  |
|  |  | |  |  | |  | |  | |  | |  | |  | |  | |  | |  | |
| **Unspecified substance** | Multiple: abuse & dependence | | Guimarães et al, 2014 [125] | Brazil  Hospital based | | Adults (18 years old and older) | | Cross-sectional study | | Male 45 (36%) & female 80 (64%). | | Non-fatal suicidal behaviour: Suicide risk | | Bivariate | | Abuse/dependence of alcohol /other drugs was not associated with suicide risk (OR=1·73, 95% CI 0·73-4·08, p>0·05). | | Null | | ++ | |
|  |  | |  |  | |  | |  | |  | |  | |  | |  | |  | |  | |
| **Unspecified substance** | Multiple: dependence & addiction | | Lavania et al, 2012 [77] | India  Hospital based | | Adults (18 to 60 years old) | | Cross-sectional study | | Male 100% | | Non-fatal suicidal behaviour: Deliberate self-harm | | Bivariate | | Deliberate self-harm among patients was associated with substance dependence (x^2^=8·40, df=1, p=0·02). | | Positive | | - | |
|  |  | |  |  | |  | |  | |  | |  | |  | |  | |  | |  | |
| **Unspecified substance** | Multiple: abuse & dependence | | Nock et al, 2009 [78] | Brazil;  Bulgaria;  Colombia;  India;  Lebanon;  Mexico;  Nigeria;  China (Beijing & Shanghai & Shenzhen);  Romania;  South Africa;  Ukraine  Community based | | Adult (18 years old and older) | | Cross-sectional study | | N/A | | Non-fatal suicidal behaviour: Suicide attempts | | Multivariate | | Multivariate interactive model (all disorders together in a discrete time survival model controlling for number of disorders as interactions) includes the following covariates: age, age-squared, age cohorts, sex, and person-year.  Drug abuse or dependency was associated with suicide attempt (OR=7·5, 95% CI 5·4–10·4, p<0·05), while controlling for age, age-squared, age cohorts, sex, and person-year. | | Positive | | + | |
|  |  |  |  |  |  |  |  |  |  |  |  | Non-fatal suicidal behaviour: Suicide attempts | | Multivariate | | Multivariate survival models of associations between type/number of prior DSM-IV disorders and subsequent suicidal behavior—developing countries.  Among the total sample, drug abuse or dependency predicted suicidal attempt (OR=2·1, 95% CI 1·2–3·6, p<0·05). | | Positive | |  |  |
|  |  |  |  |  |  |  |  |  |  |  |  | Non-fatal suicidal behaviour: Suicide attempts | | Multivariate | | Multivariate survival models of associations between type/number of prior DSM-IV disorders and subsequent suicidal behavior—developing countries.  Among the total sample, drug abuse or dependency was associated with suicide attempt (OR=4·0, 2·5–6·4, p<0·05). | | Positive | |  |  |
|  |  |  |  |  |  |  |  |  |  |  |  | Non-fatal suicidal behaviour: Suicide attempts | | Multivariate | | Multivariate survival models of associations between type/number of prior DSM-IV disorders and subsequent suicidal behavior—developing countries.  Among the total sample, drug abuse or dependency was associated with suicide attempt (OR=3·0, 95% CI 2.2–4·2, p<0·05). | | Positive | |  |  |
|  |  |  |  |  |  |  |  |  |  |  |  | Suicidal ideation | | Multivariate | | Multivariate survival models of associations between type/number of prior DSM-IV disorders and subsequent suicidal behavior—developing countries.  Among ideators, drug abuse or dependency was associated with suicide pan (OR=4·0, 95% CI (2·5–6·4, p<0·05). | | Positive | |  |  |
|  |  |  |  |  |  |  |  |  |  |  |  | Non-fatal suicidal behaviour: Suicide attempts | | Multivariate | | Multivariate survival models of associations between type/number of prior DSM-IV disorders and subsequent suicidal behavior—developing countries.  Among ideators, drug abuse or dependency was associated with a plan (OR=1·7, 95% CI 1·1–2·6, p<0·05). | | Positive | |  |  |
|  |  |  |  |  |  |  |  |  |  |  |  | Non-fatal suicidal behaviour: Suicide attempts | | Multivariate | | Multivariate survival models of associations between type/number of prior DSM-IV disorders and subsequent suicidal behavior—developing countries.  Among ideators, drug abuse or dependency was not associatedwith a planned attempt (OR=1·5, 95% CI 0·9–2·8, p>0·05). | | Null | |  |  |
|  |  |  |  |  |  |  |  |  |  |  |  | Non-fatal suicidal behaviour: Suicide attempts | | Multivariate | | Multivariate survival models of associations between type/number of prior DSM-IV disorders and subsequent suicidal behavior—developing countries.  Among ideators, drug abuse or dependency was not associated with an unplanned attempt (OR=1·4, 95% CI 0·5–4). | | Null | |  |  |
|  |  | |  |  | |  | |  | |  | |  | |  | |  | |  | |  | |
| **Unspecified substance** | Multiple: Use & abuse | | Noori et al, 2013 [122] | Iran  Others | | Adults (Mean=36; SD=8) | | Cross-sectional study | | Female 100% | | Suicidal ideation | | Multivariate | | Personal history of drug use was associated with suicidal ideation among women whose partners are drug users (OR=9·22, 95% CI 1·73-49·2, p=0·009), while controlling for age, years of marriage, and physical | | Positive | | + | |
|  |  |  |  |  |  |  |  |  |  |  |  | Non-fatal suicidal behaviour: Suicide attempts | | Multivariate | | Personal history of drug use was associated with suicide attempt among women whose spouses are drug users (OR=7·24, 95% CI 1·671-31·3, p=0·008), while controlling for age and years of marriage did not. | | Positive | |  |  |
|  |  | |  |  | |  | |  | |  | |  | |  | |  | |  | |  | |
| **Unspecifiedsubstance** | Multiple: Use & abuse | | Peltzer et al, 2008 [116] | South Africa  School based | | Adolescents (14 to 19 years old) | | Cross-sectional study | | Male 358 (30.9%) & female 799 (69.1%) | | Non-fatal suicidal behaviour: Suicide risk | | Bivariate | | Most commonly used drugs being alcohol [mean=1·57 (SD=1·6) vs. mean 1·11 (SD=1·4), t=-4·01, p<0·001], tobacco [mean=0.34 (SD=0·4) vs. mean=0·21 (SD=0·1), t=-4·10, p<0·001], cannabis [mean=0·26 (SD=0·4) vs. mean=0.14 (SD=0·3), t=-4·11, p<0·001] (dagga) and, in a small percentage, hard drugs [mean=0·19 (SD=0·7) vs. mean=0·05 (SD=0·2), t=-4·34, p<0·001].  Compared to suicide risk students, however, nonsuicide risk students reported significantly less tobacco use, alcohol use, cannabis use, combined hard drug use, and fewer drug use control problems [suicide risk mean=0·88 (SD=1) vs. nonsuicide risk mean=0·56 (SD=0·8), t=-4·37, p<0·001], and drug use consequences [suicide risk mean=1·05 (SD=0·9)] vs. nonsuicide risk mean=0.46 (SD=0·7), t=-8·24, p<0·001] than did suicide risk students. | | Positive | | ++ | |
|  |  | |  |  | |  | |  | |  | |  | |  | |  | |  | |  | |

*Note:* ^*^The parameters of the World Health Organisation is used: child = 0 to 9 years old, adolescent = 10-19 years old, and adult = 20 years old and older. ^†^Study quality: high= (++), acceptable= (+), low (-).

**Appendix C: Figures to illustrate Word Bank region, World Bank income group, study setting, substance type and substance use dimension, suicide dimension, and study design**

**Figure** **S1** Number of studies by World Bank region, as per the World Bank list of Country and

Lending Groups [28].

**Figure** **S2** Number of studies by World Bank income group, as per the World Bank list of

Country and Lending Groups [28].

**Figure** **S3** Number of studies that were conducted in each type of settlement.

**Figure S4** Number of studies that collected data from each type of setting.

**Figure S5** Type of substance used, stratified by dimension of substance use.

**Figure S6** Number of studies assessing the different suicide dimensions.

**Figure S7** Number of studies that utilised each type of study design.
